# Supplementary material for: Results of a pilot feasibility randomised controlled trial exploring the use of an electronic patient-reported outcome measure in the management of UK patients with advanced chronic kidney disease
Source: BMJ Open. 2022 Mar 18;12(3):e050610. doi: 10.1136/bmjopen-2021-050610 (PMC8935185; doi:10.1136/bmjopen-2021-050610)
Supplement: Supplementary data [file bmjopen-2021-050610supp001.pdf]

SUPPLEMENTARY APPENDIX

TITLE

Results of a pilot feasibility randomised controlled trial exploring the use of an electronic patient-reported outcome measure in the management of UK patients with advanced chronic kidney disease.

AUTHORSHIP

Derek Kyte[1,2] PhD, Nicola Anderson[2,3] MSc, Jon Bishop[4] PhD, Andrew Bissell[5], Elizabeth Brettell[4] BSc, Melanie Calvert[2,6-9,] PhD, Marie Chadburn[4] PhD, Paul Cockwell[3] PhD, Mary Dutton[3] RN, Helen Eddington[3] MB ChB, Elliot Forster[3] BSc, Gabby Hadley[3] MSc, Natalie J Ives[2,4] MSc, Louise Jackson[10] PhD, Sonia O'Brien[5], Gary Price[2,5], Keeley Sharpe[5], Stephanie Stringer[3] MB ChB, Rav Verdi[5], Judi Waters[5], Adrian Wilcockson[4].

AUTHOR AFFILIATIONS

[1] School of Applied Health & Community, University of Worcester, Worcester, UK
[2] Centre for Patient Reported Outcomes Research, Institute of Applied Health Research, College of Medical and Dental Sciences, University of Birmingham, UK.
[3] University Hospitals Birmingham NHS Foundation Trust, Birmingham, UK
[4] Birmingham Clinical Trials Unit (BCTU), Institute of Applied Health Research, University of Birmingham, Birmingham, UK
[5] Patient Advisory Group, Centre for Patient-Reported Outcomes Research, Institute of Applied Health Research, University of Birmingham, Birmingham, UK
[6] Birmingham Health Partners Centre for Regulatory Science and Innovation, University of Birmingham, Birmingham, UK
[7] National Institute for Health Research (NIHR) Birmingham Biomedical Research Centre, University of Birmingham, Birmingham, UK.
[8] National Institute for Health Research (NIHR) Applied Research Collaboration (ARC) West Midlands, University of Birmingham, Birmingham, UK
[9] National Institute for Health Research (NIHR) Surgical Reconstruction and Microbiology Research Centre University Hospitals Birmingham NHS Foundation Trust and University of Birmingham, UK
[10] Health Economics Unit, Institute of Applied Health Research, University of Birmingham, Birmingham, UK

CORRESPONDING AUTHOR

Dr Derek Kyte
Senior Lecturer
School of Allied Health and Community,
University of Worcester,
St John's Campus Henwick Grove,
Worcester,
WR2 6AJ

Email: d.kyte@worc.ac.uk

Kyte D, *et al. BMJ Open* 2022; 12:e050610. doi: 10.1136/bmjopen-2021-050610

RePROM Participant Topic Guide v1.0 – 20/11/2017

RePROM Renal Electronic Patient Reported Outcome Measures

UNIVERSITY OF BIRMINGHAM

Short project title: **RePROM**

Full project title: The use of an electronic Patient-Reported Outcome Measure in the Management of Patients with Advanced Chronic Kidney Disease – The RePROM Pilot Trial.

Participant Interview Topic Guide

Guidance notes to the interviewer

Note: *If the participant becomes distressed or unwell, the interviewer will adopt the following approaches, dependent upon the participant's wishes:*

1) If the participant wishes, the interviewer will suspend or terminate the interview, and will stay with the participant until they are feeling better.

2) If the participant has another person to provide care, at the request of the participant, the interviewer will either suspend the interview and leave the room, or will terminate the interview completely.

3) If the interviewer feels it is warranted, and if the participant agrees, he will put the participant in contact with an appropriate renal clinician.

4) If the interviewer feels that there is reason to be concerned for the physical/mental health of a participant, he will inform the participant of his intention to take the appropriate action, e.g. call the GP/Consultant.

Points to discuss with the participant prior to signing the consent form

- Recap on key information in the PIS
    - I will be recording this interview, so I have something to help me remember accurately what we talk about today, the only people who will hear the recording are myself and the person producing the transcript (who will sign a confidentiality aggrement), is this ok?
    - If there is anything you find you do not wish to talk about please let me know. I will aim to follow your lead in terms of what we discuss, but if we do stray on to a topic that you are not keen to talk about, tell me straight away and we can discuss something else.

Kyte D, *et al. BMJ Open* 2022; 12:e050610. doi: 10.1136/bmjopen-2021-050610

- We can stop the interview whenever you like. If you would like to take a break, or feel upset or unwell, please let me know and we will suspend or stop the interview entirely.

Verbal consent will be taken if participant still wishes to take part. **Note:** written consent for the interview will have been taken at the outset of the participant's involvement in the RePROM study.

Introduction to Interview

Thank you for agreeing to take part in this interview. The aim of this interview is to discuss your experience of being involved in the RePROM study. There are no 'right' or 'wrong' answers, we are interested in *your* views based on your experience. I am now going to start the recording.

Begin Interview

Main body of Interview

1) Can you explain how you first heard about the RePROM study?

2) Could you tell us what you felt was good about the recruitment process and whether any aspect could be improved?

3) What made you decide to take part in the RePROM study?

4) Can you explain what happened on your first study visit? What was good about this and what could be improved?

5) For the rest of your study visits, can you outline what was good and what could be improved?

For participants randomized to the ePROM reporting group:

6) Could you tell us about your first experience using the ePROM system?

Prompts

- Ease of myHealth sign-up and system log-in?
- Mode of administration, location, duration?
- Any problems? Ease of use?

7) Could you tell us about your subsequent experiences using the ePROM system?

Prompts

- Ease of myHealth sign-up and system log-in?
- Mode of administration, location, duration?
- Any problems? Ease of use?

Kyte D, *et al. BMJ Open* 2022; 12:e050610. doi: 10.1136/bmjopen-2021-050610

- Alert experiences?

8) Could you tell us about whether/how the ePROM information you provided was discussed in your clinic appointments?

9) Could you tell us what was good about the ePROM system and what could be improved?

Post Interview – Debrief

- I have no more questions, but I'd like to give you the opportunity to say anything else about the RePROM study, your experience of completing the ePROM, or anything else we've discussed today?

- *Outline what will happen next:* (1) the recording will be typed up and annonymised, then analysed alongside all the other interviews, (2) we will send you a summary of this interview (unless you would prefer that we didn't) and will invite your comments. You do not have to comment on these results if you do not wish to.

- Finally, if you decide that you do not want what you have said today to be included in my research, you will need to tell me this within 5 working days – so by [*insert an actual day, according to timing of interview*]. After this it will be too late to withdraw as I will not be able to untangle what you have told me from what other people have told me.

- Thank you for taking part in the interview today.

RePROM Clinician and Staff Topic Guide v1.0 – 20/11/2017

RePROM Renal Electronic Patient Reported Outcome Measures

UNIVERSITY OF BIRMINGHAM

Short project title: **RePROM**

Full project title: The use of an electronic Patient-Reported Outcome Measure in the Management of Patients with Advanced Chronic Kidney Disease – The RePROM Pilot Trial.

Clinician/Staff Interview Topic Guide

Guidance notes to the interviewer

Note: *If the participant becomes distressed or unwell, the interviewer will adopt the following approaches, dependent upon the participant's wishes:*

1) If the participant wishes, the interviewer will suspend or terminate the interview, and will stay with the participant until they are feeling better.

2) If the participant has another person to provide care, at the request of the participant, the interviewer will either suspend the interview and leave the room, or will terminate the interview completely.

3) If the interviewer feels it is warranted, and if the participant agrees, he will put the participant in contact with an appropriate renal clinician.

4) If the interviewer feels that there is reason to be concerned for the physical/mental health of a participant, he will inform the participant of his intention to take the appropriate action, e.g. call the GP/Consultant.

Points to discuss with the participant prior to signing the consent form

- Recap on key information in the PIS
    - I will be recording this interview, so I have something to help me remember accurately what we talk about today, the only people who will hear the recording are myself and the person producing the transcript (who will sign a confidentiality aggrement), is this ok?
    - If there is anything you find you do not wish to talk about please let me know. I will aim to follow your lead in terms of what we discuss, but if we do stray on to a topic that you are not keen to talk about, tell me straight away and we can discuss something else.

Kyte D, *et al. BMJ Open* 2022; 12:e050610. doi: 10.1136/bmjopen-2021-050610

- We can stop the interview whenever you like. If you would like to take a break, or feel upset or unwell, please let me know and we will suspend or stop the interview entirely.

Written consent will be taken if participant still wishes to take part.

Introduction to Interview

Thank you for agreeing to take part in this interview. The aim of this interview is to discuss your experience of being involved in the RePROM study. There are no 'right' or 'wrong' answers, we are interested in *your* views based on your experience. I am now going to start the recording.

Begin Interview

Main body of Interview

1) Could you tell us what you felt was good about the recruitment process and whether any aspect could be improved?

Prompts
- Screening, eligibility check
- Approach, consent
- myHealth signup, ePROM training
- Baseline assessment

3) Could you tell us what you felt was good about the follow-up process and whether any aspect could be improved?

6) Could you tell us about your experience using the ePROM system?

Prompts
- Ease of use, usefulness of the data?
- Format of data presentation?
- Alert generation and management.
- What was good about the system and what could be improved?

7) Is there anything about the RePROM project design or implementation that we need to address/improve prior to conducting the planned RCT?

Post Interview – Debrief

- I have no more questions, but I'd like to give you the opportunity to say anything else about the RePROM study, your experience of using the ePROM system, or anything else we've discussed today?

Kyte D, *et al. BMJ Open* 2022; 12:e050610. doi: 10.1136/bmjopen-2021-050610

- *Outline what will happen next:* (1) the recording will be typed up and annonymised, then analysed alongside all the other interviews, (2) we will send you a summary of this interview (unless you would prefer that we didn't) and will invite your comments. You do not have to comment on these results if you do not wish to.

- Finally, if you decide that you do not want what you have said today to be included in my research, you will need to tell me this within 5 working days – so by [*insert an actual day, according to timing of interview*]. After this it will be too late to withdraw as I will not be able to untangle what you have told me from what other people have told me.

- Thank you for taking part in the interview today.

Kyte D, *et al. BMJ Open* 2022; 12:e050610. doi: 10.1136/bmjopen-2021-050610

Table S1. Case Report Form (CRF) returns.

Timepoint	CRF	Expected	Received (%)
Baseline	Consent	52	52 (100)
Baseline	CRF	52	52 (100)
Baseline	EQ5D-5L	52	52 (100)
3 Month	CRF	47	47 (100)
3 Month	EQ5D-5L	47	45 (96)
6 Month	CRF	41	41 (100)
6 Month	EQ5D-5L	41	41 (100)
9 Month	CRF	29	29 (100)
9 Month	EQ5D-5L	29	29 (100)
12 Month	CRF	18	18 (100)
12 Month	EQ5D-5L	18	18 (100)

EuroQol five-level five-dimension PROM, EQ5D-5L.

Kyte D, *et al. BMJ Open* 2022; 12:e050610. doi: 10.1136/bmjopen-2021-050610

Table S2. ePROM intervention: overall symptom reporting, notifications and time taken to resolve.

Total number of participants randomised to ePROM intervention	Total number of symptoms reported	Total number of symptom notifications (%)	Total number of participants triggering notifications for severe and current symptoms (%)	Median time taken to resolve in minutes (IQR)
24	579	16 (3)	5 (25)	10 (6.5-22.5)

Electronic Patient-Reported Outcome, ePROM.

Table S3. ePROM intervention: notification pattern by symptom.

	Number of notifications triggered for severe + current symptoms (%)
Itchy/Dry skin	6 (37)
Fatigue	4 (25)
Shortness of breath	2 (13)
Pain	2 (13)
Difficulty sleeping	1 (6)
Ankle swelling	1 (6)
Lack of appetite	0 (0)
Nausea	0 (0)
Problems with fistula	0 (0)
Faintness/dizziness	0 (0)
Restless legs or difficulty keeping legs still	0 (0)
Diarrhoea	0 (0)

Electronic Patient-Reported Outcome, ePROM.

Kyte D, *et al. BMJ Open* 2022; 12:e050610. doi: 10.1136/bmjopen-2021-050610

Table S4. ePROM intervention: staff response to notification.

Staff response to notification	Frequency (%)
Telephone counselling about symptom management	7 (78)
Brought clinic appointment forwards	2 (22)
Imaging/test orders	2 (22)
Medication initiation/change	1 (11)
Other	1 (11)
Referral to A&E	0 (0)
Referral to other NHS service	0 (0)

Electronic Patient-Reported Outcome, ePROM.

Kyte D, *et al. BMJ Open* 2022; 12:e050610. doi: 10.1136/bmjopen-2021-050610

Table S5. Numeric outcome measures by trial arm and data collection point.

	Monthly ePROM reports (N = 24)		Usual care (N = 28)		
	No. (expected)	Mean (95% CI)	No. (expected)	Mean (95% CI)	Adjusted Mean Difference (95% CI)
Systolic BP (mmHg)					
Baseline	24 (24)	147.58 (139.12-156.05)	28 (28)	146.04 (139.94-152.13)	0.72 (-9.51 to 10.95)
3 months	21 (21)	145.14 (138.81-151.48)	26 (26)	140.46 (134.33-146.59)	0.13 (-7.50 to 7.76)
6 months	18 (18)	147.50 (141.92-153.08)	23 (23)	140.17 (132.33-148.02)	2.76 (-6.27 to 11.79)
9 months	11 (12)	141.91 (134.63-149.19)	16 (17)	142.19 (135.14-149.23)	-5.46 (-13.10 to 2.17)
12 months	7 (7)	148.71 (142.25-155.18)	10 (11)	137.70 (126.65-148.75)	7.87 (-5.47 to 21.20)
Diastolic BP (mmHg)					
Baseline	24 (24)	78.83 (75.22-82.45)	28 (28)	77.36 (72.94-81.77)	3.32 (-2.09 to 8.72)
3 months	21 (21)	78.81 (74.72-82.90)	26 (26)	72.85 (68.69-77.01)	4.38 (-0.40 to 9.16)
6 months	18 (18)	76.94 (70.94-82.95)	23 (23)	74.04 (69.66-78.43)	1.32 (-4.87 to 7.52)
9 months	11 (12)	78.00 (70.36-85.64)	16 (17)	78.44 (71.98-84.90)	-0.77 (-9.03 to 7.50)
12 months	7 (7)	79.00 (69.04-88.96)	10 (11)	76.90 (70.44-83.36)	0.24 (-8.92 to 9.40)
Health-Related Quality of Life (EQ-5D-5L index)					
Baseline	24 (24)	0.70 (0.60-0.80)	28 (28)	0.78 (0.71-0.85)	-0.06 (-0.17 to 0.06)
3 months	20 (21)	0.67 (0.53-0.80)	24 (26)	0.76 (0.69-0.84)	-0.03 (-0.13 to 0.07)
6 months	18 (18)	0.66 (0.52-0.80)	23 (23)	0.74 (0.65-0.82)	-0.00 (-0.11 to 0.10)
9 months	12 (12)	0.55 (0.33-0.78)	17 (17)	0.74 (0.66-0.82)	-0.07 (-0.24 to 0.09)

12 months	7 (7)	0.59 (0.34-0.85)	11 (11)	0.71 (0.61-0.82)	-0.04 (-0.17 to 0.09)
2-year Tangri[1] risk of progression to kidney failure (%)					
Baseline	24 (24)	0.48 (0.40-0.57)	28 (28)	0.43 (0.34-0.51)	0.06 (-0.01 to 0.14)
3 months	21 (21)	0.46 (0.38-0.54)	26 (26)	0.47 (0.38-0.55)	-0.01 (-0.10 to 0.08)
6 months	16 (18)	0.45 (0.34-0.57)	22 (23)	0.43 (0.35-0.52)	-0.01 (-0.12 to 0.10)
9 months	11 (12)	0.46 (0.34-0.58)	16 (17)	0.50 (0.41-0.58)	-0.04 (-0.16 to 0.08)
12 months	5 (7)	0.46 (0.29-0.63)	10 (11)	0.52 (0.38-0.66)	0.01 (-0.21 to 0.22)
eGFR (mL/min/1,73 m2)					
Baseline	24 (24)	14.03 (12.52-15.55)	28 (28)	15.70 (13.93-17.47)	-1.86 (-4.18 to 0.46)
3 months	21 (21)	13.51 (11.89-15.12)	26 (26)	14.07 (12.22-15.91)	0.94 (-0.73 to 2.61)
6 months	18 (18)	13.11 (10.93-15.29)	23 (23)	14.19 (12.49-15.89)	0.28 (-1.86 to 2.43)
9 months	11 (12)	14.54 (12.38-16.70)	16 (17)	13.13 (11.35-14.92)	2.46 (0.30 to 4.63)
12 months	7 (7)	14.13 (12.14-16.12)	10 (11)	12.71 (10.78-14.64)	1.72 (-0.96 to 4.40)
Creatinine (μmol/L)					
Baseline	24 (24)	384.00 (345.84-422.16)	28 (28)	357.54 (316.29-398.78)	39.42 (-9.71 to 88.54)
3 months	21 (21)	380.81 (346.19-415.43)	26 (26)	396.08 (342.23-449.92)	-34.81 (-66.83 to -2.79)
6 months	18 (18)	408.39 (359.35-457.43)	23 (23)	375.96 (334.91-417.00)	-17.82 (-57.55 to 21.92)
9 months	11 (12)	364.45 (305.24-423.67)	16 (17)	399.50 (347.47-451.53)	-41.90 (-88.94 to 5.13)
12 months	7 (7)	370.00 (306.19-433.81)	10 (11)	409.10 (337.29-480.91)	-47.60 (-131.55 to 36.36)
Calcium (μmol/L)					
Baseline	24 (24)	2.24 (2.19-2.29)	28 (28)	2.27 (2.25-2.30)	-0.03 (-0.09 to 0.02)
3 months	21 (21)	2.28 (2.22-2.35)	26 (26)	2.29 (2.24-2.34)	0.02 (-0.04 to 0.08)

Kyte D, *et al. BMJ Open* 2022; 12:e050610. doi: 10.1136/bmjopen-2021-050610

6 months	18 (18)	2.30 (2.25-2.35)	23 (23)	2.34 (2.29-2.39)	-0.01 (-0.07 to 0.04)
9 months	11 (12)	2.37 (2.27-2.47)	16 (17)	2.40 (2.35-2.46)	-0.03 (-0.11 to 0.04)
12 months	6 (7)	2.40 (2.35-2.45)	10 (11)	2.40 (2.29-2.50)	0.01 (-0.08 to 0.10)
Bicarbonate (µmol/L)					
Baseline	24 (24)	20.83 (19.76-21.89)	28 (28)	21.25 (20.33-22.17)	-0.30 (-1.70 to 1.09)
3 months	21 (21)	21.36 (20.13-22.59)	25 (26)	21.30 (20.16-22.45)	0.19 (-1.26 to 1.64)
6 months	17 (18)	20.56 (19.14-21.99)	21 (23)	21.19 (19.97-22.41)	0.49 (-0.92 to 1.91)
9 months	11 (12)	21.82 (19.59-24.04)	15 (17)	20.73 (19.14-22.33)	1.13 (-1.32 to 3.59)
12 months	5 (7)	21.60 (18.93-24.27)	9 (11)	20.67 (17.76-23.57)	1.03 (-2.44 to 4.50)
Phosphate (µmol/L)					
Baseline	24 (24)	1.41 (1.31-1.52)	28 (28)	1.40 (1.30-1.51)	0.01 (-0.14 to 0.16)
3 months	21 (21)	1.47 (1.39-1.55)	25 (26)	1.60 (1.41-1.79)	-0.14 (-0.34 to 0.05)
6 months	17 (18)	1.52 (1.36-1.69)	21 (23)	1.38 (1.23-1.52)	0.06 (-0.12 to 0.25)
9 months	11 (12)	1.45 (1.27-1.62)	14 (17)	1.46 (1.30-1.61)	-0.03 (-0.26 to 0.21)
12 months	5 (7)	1.61 (1.28-1.93)	9 (11)	1.42 (1.25-1.60)	0.31 (0.02 to 0.59)
Albumin (g/L)					
Baseline	24 (24)	40.38 (38.20-42.55)	28 (28)	40.82 (38.98-42.66)	-0.52 (-3.34 to 2.30)
3 months	21 (21)	39.43 (37.50-41.36)	26 (26)	39.58 (37.80-41.36)	0.91 (-0.61 to 2.43)
6 months	18 (18)	37.39 (35.15-39.62)	23 (23)	37.65 (35.90-39.41)	0.24 (-1.56 to 2.04)
9 months	11 (12)	35.27 (33.12-37.42)	16 (17)	36.50 (34.52-38.48)	0.37 (-2.31 to 3.05)
12 months	7 (7)	36.86 (34.42-39.29)	10 (11)	35.10 (32.90-37.30)	1.63 (-1.38 to 4.64)
ACR (mg/mmol)					
Baseline	24 (24)	206.06 (126.92-285.20)	28 (28)	178.08 (109.73-246.43)	23.64 (-66.09 to 113.37)

Kyte D, *et al. BMJ Open* 2022; 12:e050610. doi: 10.1136/bmjopen-2021-050610

3 months	21 (21)	167.31 (101.53-233.09)	26 (26)	149.25 (108.39-190.11)	-19.60 (-63.75 to 24.56)
6 months	16 (18)	182.24 (95.65-268.83)	22 (23)	135.88 (88.78-182.98)	-3.73 (-72.53 to 65.07)
9 months	11 (12)	227.58 (117.37-337.79)	16 (17)	148.23 (97.56-198.90)	0.20 (-84.56 to 84.96)
12 months	5 (7)	175.74 (97.71-253.77)	10 (11)	161.51 (74.67-248.35)	-14.40 (-138.43 to 109.63)
Blood Glucose (mmol/L)					
Baseline	8 (9)	8.36 (6.82-9.90)	11 (12)	6.97 (5.58-8.36)	1.48 (-0.57 to 3.52)
3 months	7 (9)	9.36 (5.39-13.33)	8 (11)	8.74 (5.80-11.68)	-2.18 (-6.22 to 1.87)
6 months	5 (8)	15.88 (3.47-28.29)	5 (10)	7.22 (5.14-9.30)	-2.58 (-13.52 to 8.36)
9 months	4 (6)	8.93 (5.36-12.49)	3 (8)	6.30 (3.84-8.76)	2.12 (-1.40 to 5.64)
12 months	1 (4)	10.70[#]	2 (5)	5.10 (1.57-8.63)	-
HbA1c (mmol/mol)					
Baseline	5 (9)	57.20 (42.83-71.57)	9 (12)	53.22 (43.98-62.46)	3.18 (-12.52 to 18.87)
3 months	7 (9)	53.29 (43.78-62.79)	7 (11)	46.14 (38.80-53.48)	2.36 (-4.61 to 9.33)
6 months	7 (8)	51.14 (44.40-57.88)	8 (10)	50.63 (40.45-60.80)	-6.00 (-14.06 to 2.05)
9 months	2 (6)	59.50 (52.64-66.36)	3 (8)	52.67 (43.04-62.29)	-
12 months	2 (4)	57.00 (51.12-62.88)	3 (5)	49.33 (36.87-61.80)	-6.58 (-9.21 to -3.96)

[#]Insufficient data to calculate 95% CI. [1] Tangri N, Stevens LA, Griffith J, et al. A predictive model for progression of chronic kidney disease to kidney failure. Jama. 2011;305(15):1553-1559.[21] Electronic Patient-Reported Outcome Measure, ePROM; blood pressure, BP; EuroQol five-level five-dimension PRO, EQ5D-5L; Estimated Glomerular Filtration Rate, eGFR; Albumin Creatinine Ratio, ACR; glycated haemoglobin, HbA1c.

Table S6. Binary outcome measures by trial arm and data collection point.

	Monthly ePROM reports (N = 24)		Usual care (N = 28)		
	N[a]	Events (%, 95% CI)	N[a]	Events (%, 95% CI)	Risk Ratio (95% CI)[b]
**Death**					
Baseline to 3 months	24	0 (0, 0-14)	28	0 (0, 0-12)	-
3 to 6 months	21	1 (5, 0-24)	26	0 (0, 0-13)	-
6 to 9 months	18	0 (0, 0-19)	23	0 (0, 0-15)	-
9 to 12 months	12	0 (0, 0-26)	17	0 (0, 0-20)	-
Total		1 (4, 0-21)		0 (0, 0-12)	-
**Kidney failure**					
Baseline to 3 months	24	1 (4, 0-21)	28	4 (14, 4-33)	0.29 (0.30 to 2.44)
3 to 6 months	21	3 (14, 3-36)	26	2 (8, 1-25)	1.86 (0.34 to 10.11)
6 to 9 months	18	3 (17, 4-41)	23	0 (0, 0-15)	-
9 to 12 months	12	1 (8, 0-38)	17	3 (18, 4-43)	0.47 (0.06, 4.01)
Total		8 (33, 16-55)		9 (32, 16-52)	1.04 (0.47 to 2.26)
**Hospitalisation**					
Baseline to 3 months	24	1 (4, 0-12)	28	1 (4, 0-18)	1.17 (0.08 to 17.67)
3 to 6 months	21	2 (10, 1-30)	26	3 (6, 2-30)	0.83 (0.15 to 4.49)
6 to 9 months	19	2 (11, 1-33)	23	2 (9, 1-28)	1.21 (0.19 to 7.80)
9 to 12 months	12	0 (0, 0-26)	17	0 (0, 0-20)	-
Total		5 (21, 7-42)		5[c] (18, 6-37)	1.17 (0.38 to 3.55)

Electronic patient-reported outcome, ePROM. [a]Number of participants in the study at start of timepoint.
[b]unadjusted risk ratios are reported due to the low frequencies of events. [c]This figure denotes the number of unique individuals with at least one hospital stay during the study. Individuals can have more than one hospital stay.

Kyte D, *et al. BMJ Open* 2022; 12:e050610. doi: 10.1136/bmjopen-2021-050610

Table S7. Protocol deviations.

	Allocation	
Protocol deviation	Monthly ePROM reports (N = 24)	Usual care (N = 28)
Software error 19-Jun-2019 [resolved]	1	0
Informed Consent Form error	0	1

Electronic Patient-Reported Outcome, ePROM.

Kyte D, *et al*. *BMJ Open* 2022; 12:e050610. doi: 10.1136/bmjopen-2021-050610

Table S8. Free text comments.

If you have had any other symptoms or problems that you would like the kidney team to be aware of please outline below:
A stomach upset overnight one evening. with indigestion. Resolved by taking a couple of Bisodol tablets
Anal fistulas
Ankle and lower leg swelling since [Date Redacted]. New symptom. Goes away overnight. No new shortness of breath.
Arthritis
Arthritis. psoriasis. diabetes. high blood pressure
Arthritis/psoriasis
been very pale and colleagues have commented on a "yellow" tinge
Blocked sinus's
Breathlessness increasing. Clinic [Date Redacted] - fluid at base of right lung
constipation
constipation. which is improving
Cough productive of clear mucus
Difficulty concentrating
Difficulty concentrating and feeling cold
Difficulty concentrating. Night swears.
Dry mouth. husky voice.
During last night's sleep. I woke up in the middle of the night [Date Redacted] and found that my pyjama top was soaked in sweat. Otherwise. felt OK?
During my last visit to the Renal team. Quinine Sulfate tablets were proscribed to assist with random over night leg cramps. Just to confirm that this medication has dramatically reduced the incidence of cramps. thank you.
Excessive mucus. no cold symptoms. but caused me to vomit and retch. Slight nosebleeds. Very poor appetite. UTI. Antibiotics prescribed by renal vascular team [Date Redacted] when doing first stage fistula. Ciprox
Feel a bit light headed this afternoon
Feeling cold
Feeling cold.
Felt very tired on [Dates Redacted] plus a stomach upset. probably as a result of the proceedure carried out [Date Redacted]?

Kyte D, *et al. BMJ Open* 2022; 12:e050610. doi: 10.1136/bmjopen-2021-050610

For the last two nights I have had difficulty in sleeping after the first three hours or so. Additionally last night when I awoke in the middle of the night for a toilet break I had been sweating a very great deal. which is unusual for me.
Headaches. painful feet. like electric shocks
Increasing sleepiness. eg nodding off after meals
Inpatient [Dates redacted]
Joint swelling...pain in joints...headaches
Loss of taste
More sleepy' Prone to nod off
My bladder control is proving difficult. especially if I travel any distance. After two hours traveling. I often need to stop to empty my bladder and don't get much warning. This means I have to always be on the look out for a toilet where ever I go.
no
No
Not that I'm aware of.
No.
None
none
None
None at this time.
none known
None known
None.
Not that I am aware of
Not that I know of.
Pedal oedema - This was the presenting symptom to the team
Productive cough
Rash over upper body in small patches
Really bad cold
Severe and constant gout inflammatory knee joint

Severe headaches
Since [Date Redacted] I have had swollen ankles and legs. This goes away overnight. This is a new symptom. I have not been SOB.
Sleepiness previously reported has improved
Some nights I have been getting up three times to pass urine. However. I have just been given compression stockings by a Lymphoedema clinic to help with my swollen legs caused by taking Felodipine (mostly). This might help the problem...
Swelling in ankles due to hospitalisation. diarrhoea due to IV antibiotics for eye infection
Tending to drift off to sleep during the day more often
The Kidney team is aware and treatment is ongoing
Wheezy cough

Kyte D, *et al*. *BMJ Open* 2022; 12:e050610. doi: 10.1136/bmjopen-2021-050610

Table S9. Summary of qualitative findings regarding intervention positives/negatives and suggested system changes based on 1 HCP interview.

Theme	Subtheme	Illustrative quote
Intervention positives	Questionnaire data picked up by care team and acted upon	"I would always start the consultation with thank you for taking part, I've been looking at this, shall we look at it together, I see that here you reported this, would you like to tell me a bit about that… patients… seemed really pleased that we were looking at it and using it and it was meaningful. Because clearly it was something that they were taking time and trouble to do. And so, for them knowing that we were using it and taking it seriously was probably a really good thing." [HCP 01]
	Used free-text comments to communicate with nursing staff	"Initially I was filling the form in and putting very little additional information on. Latterly I was putting a lot more information on and I was very pleased on two occasions that when I went for my renal check-up, the points that I'd made had been noticed and were brought up… it was an additional form of communication in that if I'd got a concern or something was happening, I could put it on the form… and you could use it to answer questions then as to how you were coping, what you were doing and how you were feeling." [Patient 01]; "I think that was the good thing about the free text because it did allow people to tell us things that we hadn't particularly asked about." [HCP 01]
	Useful tool to guide consultation	"It was a nice tool to guide consultation. So normally you've just got your clinic letter from your previous visit, and that gives you a fair idea of the kind of things that you're going to talk to the patient about based on the things that you've talked to them about before and the active medicine which you've identified. But having the RePROM as well often highlighted things that were completely off the radar. And I think it's perfectly likely the patient would have mentioned it themselves anyway, it meant that you knew in advance and you were able to get straight into it, rather than it being the kind of thing that they casually mention as they're leaving the room. So, you have a bit more time to explore things in a bit more detail I think." [HCP 01]
	Would allow remote follow up post-COVD	"…now our capacity to see patients face-to-face has reduced by about 75% because of the need for social distancing. So actually, now that they're almost all phone and video consultations something like RePROM is more important than ever because that does give patients a bit more of an ability to… to contact us and tell us things that they were worried about in between their reviews." [HCP 01]

Kyte D, et al. BMJ Open 2022; 12:e050610. doi: 10.1136/bmjopen-2021-050610

Intervention negatives	Need to open up a different system precluded use in Multidisciplinary Team (MDT) meetings	"We had lots of great ideas at the beginning about how we'd look at it and the MDT when we looked forward to the next clinic but actually the MDT's are so busy and there were so many people to get through that it just a quick, look at the blurb, what are the outstanding issues, move on. And so, we didn't use it because that would have meant getting the Portal up rather than just PICS and waiting for it to load and so no, we didn't use it in the MDT." [HCP 01]
Intervention acceptability	Patient acceptance of remote follow up/ability to engage with technology	"I guess COVID has taught us a couple of things. The first thing is that we've all said, a lot of people have said, oh patients won't cope with phone consultations, and they certainly won't cope with video consultations. Patients are not very tech savvy, they won't be able to do it, they're all very elderly, a lot of them don't speak any English and it would be a complete disaster. And that's not completely been our experience, people seem to have adapted to phone consultations and video consultations really quite well." [HCP 01]
	Enhance/simplify interface	"The only thing I can think of as far as improving the system is to make it more user-friendly basically… navigating your way through the electronic system… could be made a bit easier." [Patient 08]; "I think the practical obstacle… was that patients find the interface difficult." [HCP 01]
	Incorporate questions around psychological wellbeing/mood	"I think just having that questionnaire to see how your mood is and how you can look back on it and see where, like, how you can improve and how you can change it slightly and try and move on from there…" [Patient 10]; "I'm not particularly surprised that people mentioned that [anxiety & depression], and I think that's reasonable. I think in a future iteration we probably should try and capture that." [HCP 01]
	Consider expanding use of the system to dialysis populations	"I definitely think that doing something like this in terms of the dialysis population would be massively useful… Compared to the very close supervision that they had in the year, six months before they started dialysis. A year to six months after they've started dialysis that is an entirely different experience… anecdotally a lot of patients say, oh gosh I used to come to clinic and see doctors and nurses and dieticians and now I'm at my satellite unit I see the nurses all the time and I occasionally see a dietician but it doesn't feel the same… I think they find that quite a worrying time, and maybe having something like this to support them particularly in that transition would be really useful." [HCP 01]
	Consider use of a central platform to aid roll out to other centres	"I think the difficulty when we think about rolling it out to other places is that everywhere will have a different electronic patient record type system… we'll have to think about how the IT works in each of those places..." [HCP 01]

Kyte D, *et al. BMJ Open* 2022; 12:e050610. doi: 10.1136/bmjopen-2021-050610
