# Supplementary material for: Results of a pilot feasibility randomised controlled trial exploring the use of an electronic patient-reported outcome measure in the management of UK patients with advanced chronic kidney disease
Source: BMJ Open. 2022 Mar 18;12(3):e050610. doi: 10.1136/bmjopen-2021-050610 (PMC8935185; doi:10.1136/bmjopen-2021-050610)
Supplement: Author's manuscript [file bmjopen-2021-050610.draft_revisions.pdf]

BMJ Open is committed to open peer review. As part of this commitment we make the peer review history of every article we publish publicly available.

When an article is published we post the peer reviewers' comments and the authors' responses online. We also post the versions of the paper that were used during peer review. These are the versions that the peer review comments apply to.

The versions of the paper that follow are the versions that were submitted during the peer review process. They are not the versions of record or the final published versions. They should not be cited or distributed as the published version of this manuscript.

BMJ Open is an open access journal and the full, final, typeset and author-corrected version of record of the manuscript is available on our site with no access controls, subscription charges or pay-per-view fees (http://bmjopen.bmj.com).

If you have any questions on BMJ Open's open peer review process please email info.bmjopen@bmj.com

BMJ Open

Use of an electronic patient-reported outcome measure in the management of patients with advanced chronic kidney disease: the RePROM pilot trial

Journal:	*BMJ Open*
Manuscript ID	bmjopen-2021-050610
Article Type:	Original research
Date Submitted by the Author:	24-Feb-2021
Complete List of Authors:	Kyte, Derek; University of Worcester,<br>Anderson, Nicola; University Hospitals Birmingham NHS Foundation Trust<br>Bishop, Jon; Birmingham Clinical Trials Unit (BCTU), Institute of Applied Health Research, University of Birmingham, Birmingham, UK, Medical Statistician<br>Bissell, Andrew; University of Birmingham, Patient Advisory Group, Centre for Patient-Reported Outcomes Research, Institute of Applied Health Research<br>Brettell, Elizabeth; 1. Birmingham Clinical Trials Unit (BCTU), Institute of Applied Health Research University of Birmingham, Birmingham, UK, Birmingham Clinical Trials Unit<br>Calvert , Melanie ; University of Birmingham, Centre for Patient Reported Outcomes Research and Institute of Applied Health Research<br>Chadburn, Marie; University of Birmingham, Birmingham Clinical Trials Unit, Institute of Applied Health Research<br>Cockwell, Paul; Queen Elizabeth Hospital Birmingham, Department of Renal Medicine; University of Birmingham, Division of Infection and Immunity<br>Dutton, Mary; University Hospitals Birmingham NHS Foundation Trust, Renal Medicine<br>Eddington, Helen; University Hospitals Birmingham NHS Foundation Trust, Renal Medicine<br>Forster, Elliot; University Hospitals Birmingham NHS Foundation Trust<br>Hadley, Gabby; University Hospitals Birmingham NHS Foundation Trust, Renal Medicine<br>Ives, Natalie; University of Birmingham, BCTU<br>Jackson, Louise; University of Birmingham, Health Economics Unit<br>O'Brien, Sonja; University of Birmingham, Institute of Applied Health Research<br>Price, Gary; University of Birmingham, Patient Advisory Group Member, Centre for Patient-Reported Outcomes Research (CPROR)<br>Sharpe, Keeley; University of Birmingham, Patient Advisory Group Member, Centre for Patient-Reported Outcomes Research (CPROR)<br>Stringer, Stephanie; University Hospitals Birmingham NHS Foundation Trust, Renal Medicine<br>Verdi, Rav; University of Birmingham, Patient Advisory Group Member, Centre for Patient-Reported Outcomes Research (CPROR)<br>Waters, Judi; University of Birmingham, Patient Advisory Group Member, Centre for Patient-Reported Outcomes Research (CPROR)

	Wilcockson, Adrian; University of Birmingham

Keywords:	NEPHROLOGY, End stage renal failure < NEPHROLOGY, Clinical trials < THERAPEUTICS

SCHOLARONE™
Manuscripts

I, the Submitting Author has the right to grant and does grant on behalf of all authors of the Work (as defined in the below author licence), an exclusive licence and/or a non-exclusive licence for contributions from authors who are: i) UK Crown employees; ii) where BMJ has agreed a CC-BY licence shall apply, and/or iii) in accordance with the terms applicable for US Federal Government officers or employees acting as part of their official duties; on a worldwide, perpetual, irrevocable, royalty-free basis to BMJ Publishing Group Ltd ("BMJ") its licensees and where the relevant Journal is co-owned by BMJ to the co-owners of the Journal, to publish the Work in this journal and any other BMJ products and to exploit all rights, as set out in our licence.

The Submitting Author accepts and understands that any supply made under these terms is made by BMJ to the Submitting Author unless you are acting as an employee on behalf of your employer or a postgraduate student of an affiliated institution which is paying any applicable article publishing charge ("APC") for Open Access articles. Where the Submitting Author wishes to make the Work available on an Open Access basis (and intends to pay the relevant APC), the terms of reuse of such Open Access shall be governed by a Creative Commons licence – details of these licences and which Creative Commons licence will apply to this Work are set out in our licence referred to above.

Other than as permitted in any relevant BMJ Author's Self Archiving Policies, I confirm this Work has not been accepted for publication elsewhere, is not being considered for publication elsewhere and does not duplicate material already published. I confirm all authors consent to publication of this Work and authorise the granting of this licence.

For peer review only

TITLE

Use of an electronic patient-reported outcome measure in the management of patients with advanced chronic kidney disease: the RePROM pilot trial

AUTHORSHIP

Derek Kyte[1,2] PhD, Nicola Anderson[2,4] MSc, Jon Bishop[5] PhD, Andrew Bissell[6], Elizabeth Brettell[5] BSc, Melanie Calvert[2,3] PhD, Marie Chadburn[5] PhD, Paul Cockwell[4] PhD, Mary Dutton[4] RN, Helen Eddington[4] MB ChB, Elliot Forster[4] BSc, Gabby Hadley[4] MSc, Natalie J Ives[2,5] MSc, Louise Jackson[7] PhD, Sonia O'Brien[6], Gary Price[2,6], Keeley Sharpe[6], Stephanie Stringer[4] MB ChB, Rav Verdi[6], Judi Waters[6], Adrian Wilcockson[5].

AUTHOR AFFILIATIONS

[1]School of Applied Health & Community, University of Worcester, Worcester, UK
[2] Centre for Patient-Reported Outcomes Research, Institute of Applied Health Research, University of Birmingham, Birmingham, UK
[3] NIHR Birmingham Biomedical Research Centre, University of Birmingham and University Hospitals Birmingham NHS Foundation Trust, UK
[4] University Hospitals Birmingham NHS Foundation Trust, Birmingham, UK
[5] Birmingham Clinical Trials Unit (BCTU), Institute of Applied Health Research, University of Birmingham, Birmingham, UK
[6] Patient Advisory Group, Centre for Patient-Reported Outcomes Research, Institute of Applied Health Research, University of Birmingham, Birmingham, UK
[7] Health Economics Unit, Institute of Applied Health Research, University of Birmingham, Birmingham, UK

CORRESPONDING AUTHOR

Dr Derek Kyte
Lecturer in Health Research Methods
NIHR Fellow
The Murray Learning Centre
University of Birmingham
Birmingham
B15 2TT

Telephone: 0121 415 8502
Email: d.g.kyte@bham.ac.uk

KEYWORDS

Chronic Kidney Disease, patient-reported outcomes, symptom monitoring, pilot trial, randomised controlled trial

WORDCOUNT

3887

For peer review only

ABSTRACT

Objectives

The use of routine remote follow-up of patients with chronic kidney disease (CKD) is increasing exponentially. It has been suggested that online electronic patient-reported outcome measures (ePROMs) could be used in parallel, to facilitate real-time symptom monitoring aimed at improving outcomes. We tested the feasibility of this approach in a pilot trial of ePROM symptom monitoring versus usual care in patients with advanced CKD not on dialysis.

Design

A 12-month, parallel, pilot randomised controlled trial (RCT) and qualitative sub-study.

Setting & Participants

Queen Elizabeth Hospital Birmingham, UK. Adult patients with advanced CKD (eGFR ≥6 and ≤15 mL/min/1.73m^2, or a projected risk of progression to kidney failure within 2-years ≥20%).

Intervention

Monthly online ePROM symptom reporting, including automated feedback of tailored self-management advice and triggered clinical notifications in the advent of severe symptoms. Real-time ePROM data were made available to the clinical team via the electronic medical record.

Outcomes

Feasibility (recruitment and retention rates, and acceptability/adherence to the ePROM intervention). Health-related quality of life, clinical data (e.g., measures of kidney function, kidney failure, hospitalisation, death) and healthcare utilisation.

Results

52 patients were randomised (31% of approached). Case report form returns were high (99.5%), as was retention (96%). Overall, 73% of expected ePROM questionnaires were received. Intervention adherence was high beyond 90 days (74%) and 180 days (65%); but dropped beyond 270 days (46%). Qualitative interviews supported proof of concept and intervention acceptability, but highlighted necessary changes aimed at enhancing overall functionality/scalability of the ePROM system.

Limitations

Small sample size.

Conclusions

This pilot trial demonstrates that patients are willing to be randomised to a trial assessing ePROM symptom monitoring. The intervention was considered acceptable; though measures to improve longer-term engagement are needed. A full-scale RCT is considered feasible.

Trial Registration

ISRCTN12669006

For peer review only

STRENGTHS AND LIMITATIONS OF THIS STUDY

- This is the first UK study conducted in a CKD population that has explored the feasibility of ePROM capture/feedback with real-time integration within the electronic medical record.
- This study is reported in accordance with the Consolidated Standards of Reporting Trials (CONSORT) checklist for reporting a pilot/feasibility trial.
- As this was a pilot study, no inferences can be made about the intervention's therapeutic efficacy. Our findings will instead help guide the design of a future randomised controlled trial aimed at exploring efficacy and cost effectiveness.

For peer review only

BACKGROUND

Patients with advanced chronic kidney disease (CKD) commonly have a high symptom burden; increasingly so as they progress towards kidney failure.[1 2] Uncontrolled symptomology can be a particular source of anxiety and can have a detrimental impact on patient's health-related quality of life and outcomes.[1-3]

Timely detection of symptomatic deterioration is a key component of effective disease management during this period.[3] It can be challenging, however, to identify an unexpected decline in kidney function between scheduled clinic appointments, unless a patient self-refers. Unfortunately, some patients self-refer too late because they have difficulty identifying the point at which they may require assistance. Without prompt recognition of advanced symptoms, such patients are at high risk of severe illness, emergency hospitalisation, progression to unplanned kidney replacement therapy and significantly poorer long-term outcomes, including increased mortality.[4-6]

Routine systematic capture of symptom data using electronic patient-reported outcome (ePROM) measures has been suggested as a low-cost method of supporting symptom monitoring and control.[7] ePROM platforms provide patients with access to short online questionnaires that allow them to share self-reported symptom data with their clinical team, often in real time, to help guide care.[8] Systems may be configured to provide patients with tailored self-management advice and to trigger clinical notifications in the advent of sudden deterioration and/or severe symptomology.[9-11]

In studies involving patients with cancer, ePROM symptom monitoring is associated with enhanced patient-clinician communication; improved patient education and self-efficacy; better symptom control; earlier detection of adverse events; improved patient quality of life; reduced use of accident and emergency services; fewer inpatient hospital episodes; and improved survival; even for 'computer-inexperienced' patients.[9-17]

The efficacy of ePROM symptom monitoring for patients with advanced CKD, has not been investigated within a randomised controlled trial (RCT); nor has the feasibility of undertaking such a trial been established. This single-center pilot study aimed to assess the feasibility of undertaking a RCT investigating the use of monthly ePROM reporting compared with usual care in patients with advanced CKD not on dialysis.

For peer review only

METHODS

Reporting
This study is reported in accordance with the Consolidated Standards of Reporting Trials (CONSORT) checklist for reporting a pilot/feasibility trial.[18]

Study Design
RePROM (Renal electronic patient-reported outcome measure) was a single-centre, open-label, two-arm randomised controlled pilot/feasibility trial and qualitative sub-study. The trial was registered with ISRCTN (ISRCTN12669006) and the UK NIHR Portfolio (CPMS ID: 36497); and the protocol has been published.[19] This study was approved by the West Midlands Edgbaston Research Ethics Committee (Ref: 18/WM/0013) on 23rd February 2018 (ePROM finalisation and pilot trial).

Study changes
Owing to changes in clinical practice at the host research site, made in response to the COVID-19 pandemic, the study received approval from the Health Research Authority for early closure of follow up (02/04/2020). This meant that follow up was truncated for some participants and that recruitment of health care professionals (HCPs) to the qualitative sub-study had to be suspended.

Study setting
The trial was undertaken within the Birmingham Clinical Trials Unit (BCTU) and Centre for Patient-Reported Outcomes Research at the University of Birmingham and the Queen Elizabeth Hospital Birmingham (QEHB) within the UK National Health Service (NHS) University Hospitals Birmingham Foundation Trust.

Patient and public involvement
Development of the study design was informed by a series of meetings held with our Patient Advisory Group (AB, SO, GP, KS, RV, JW), established in 2016, which included people with lived experience of CKD. Members were also involved in the ePROM intervention co-design group[20] and trial management group.

Study oversight
An independent steering committee was convened to provide guidance to the trial management group and to review feasibility data during the trial.

Study population
Eligible participants were adult (≥18 years old) patients under the care of the kidney services at QEHB, who met the trial definition of advanced CKD (estimated Glomerular Filtration Rate (eGFR) ≥6 and ≤15 mL/min/1.73m^2, or a projected risk of progression to kidney failure within 2-years ≥20% using the 4-variable Tangri renal risk equation[21]). Participants were excluded if they met any of the following criteria: patients unwilling to use the ePROM intervention; patients who, in the opinion of the consenting professional, could not speak, read or write English sufficiently well to complete the ePROM unaided; an episode of acute kidney injury (defined in accordance with international guidelines[22]) within the last 3 months; patients meeting the trial definition of kidney failure (receiving dialysis, or scheduled to start, in the next 2 weeks, had received (or had a scheduled date to receive) a kidney transplant; or an eGFR ≤5ml/min/1.73m2); patients with a terminal illness that, in the opinion of the

For peer review only - http://bmjopen.bmj.com/site/about/guidelines.xhtml

clinician assessing eligibility, was likely to lead to the death of the patient within 6 months of starting participation in the study.

Recruitment and randomisation

Members of the kidney research team at QEHB screened for potentially eligible study participants using the inclusion/exclusion criteria. Those considered eligible were provided with a patient information sheet and given the opportunity to consider participation. For patients wishing to take part in the pilot trial (and optional qualitative sub-study), consent, enrolment and baseline data collection was conducted face-to-face in clinic. Randomisation was provided via a web-based system developed by BCTU. Participants were randomised at the level of the individual in a 1:1 ratio to usual care (control arm) or usual care supplemented with monthly online symptom reporting using the ePROM system (experimental arm). Minimisation was used to achieve balance between: 2-year risk of progression to kidney failure ($<40\%$, versus $\geq 40\%$, based on the 4-variable Tangri renal risk equation[21]); self-reported computer experience (regular use of a computer, tablet or smartphone at least weekly, versus less than weekly); and patient-reported ethnicity ('white' versus 'non-white').

Intervention

Participants allocated to the ePROM intervention arm were asked to complete and submit monthly symptom questionnaires using an online system and received an automated reminder to do so. In addition, patients were allowed to submit any number of additional 'ad-hoc' questionnaires at any time outside of the scheduled monthly reporting dates. Development and functionality of the ePROM system has been described in detail elsewhere.[20] In summary, upon questionnaire submission, automated self-management advice was provided to patients based on their responses; questionnaire data was integrated into the QEHB electronic medical record and made available to HCPs in real-time; and a system algorithm triggered an automated notification which was sent to both the patient and the clinical team in the event of a severe and current symptom report. Participants allocated to the control arm received usual care. It was not possible to blind clinicians or participants due to the nature of the intervention.

Outcomes

As this was a pilot trial there was no single primary outcome measure. The primary aims of the study were to pilot the trial protocol and assess the feasibility of undertaking a full-scale RCT exploring the use of ePROMs in the management of advanced CKD. The feasibility outcomes included the following: the proportion of eligible participants approached to take part in the trial; the proportion of eligible participants who took part in the trial; recruitment rate: the proportion of participants randomised / screened; the proportion of participants randomised / approached; the proportion of participants who completed the trial (retention); and the proportion of participants who adhered to the ePROM intervention.

This pilot trial was not powered to detect differences in outcome measures, but provided an opportunity to ensure that there were no issues with completion of the outcome data and proposed outcome measures for the main RCT. The following outcome data were collected:

- Health-related quality of life, using the paper version of the EuroQol five-dimension, five level, questionnaire (EQ-5D-5L). The EQ-5D-5L is a reliable/validated generic

For peer review only - http://bmjopen.bmj.com/site/about/guidelines.xhtml

measure of health status/utility commonly used internationally in cost-effectiveness and ePROM research.[10 23]

- Clinical data, including serum creatinine, calcium, phosphate, bicarbonate, albumin, eGFR, albumin-to-creatinine ratio (ACR), blood pressure, and, for participants with diabetes: glucose and glycated haemoglobin (HbA1c).
- Event data: progression to kidney failure, contacts with healthcare professionals in secondary care (outpatient clinics and accident and emergency), inpatient hospitalisation, death.
- Additional healthcare resource use data was also collected at each study visit.

All data were collected at baseline and 3, 6, 9 and 12 months (assessment window ±3 weeks).

Sample size
As this was a pilot trial, no formal sample size calculation was performed. Following recommendations for pilot studies, 30 patients or more are typically required to obtain estimates of the parameters needed for sample size estimation.[24 25] To allow for a 10% drop-out and loss to follow-up, this pilot trial aimed to recruit at least 33 participants in each group, a total of 66 participants. This would allow the recruitment and retention rates to be estimated with 95% confidence interval maximum widths of 20% and 25% respectively.

Statistical analysis
Analysis of feasibility and clinical outcomes was based on all participants screened and recruited. For each binary outcome, the number and percentage are reported along with an exact binomial 95% confidence interval. Estimates of differences between groups are presented as relative risks obtained from log-binomial regression models. These estimates were unadjusted due to the low number of observed events. For continuous outcomes, the means and 95% confidence intervals are reported. Estimates of differences between groups are presented as differences in means adjusted for minimisation variables and, for longitudinal outcomes, the corresponding baseline values. All estimates of differences are presented with 95% confidence intervals. No p-values are reported as no hypothesis testing was performed. Analysis was conducted using SPSS software, v26 (IBM) and SAS software, version 9.4 (SAS Institute). Participants were analysed in the intervention group to which they were randomised, and all participants were included whether or not they received the allocated intervention (intention-to-treat). The study dataset and statistical analysis plan are available on request.

Qualitative sub-study
The qualitative sub-study explored patient and HCP thoughts/experiences regarding the RePROM trial processes and intervention. Semi-structured interviews were conducted by the lead author according to pre-defined topic guides (Supplementary Appendix), but there was sufficient scope to explore novel themes where appropriate. All interviews were digitally recorded, professionally transcribed and the transcripts anonymised. Transcript data were entered into a specialist software package (Dedoose, v8.3.35) to aid organisation and analysis of the data. All data were analysed by the lead author using conventional content analysis.[26]

For peer review only

RESULTS

Patients and follow up

Recruitment was conducted at QEHB over 12 months from October 2019. The last follow-up was conducted in April 2020, which was truncated for 14 participants due to the COVID-19 pandemic. In total, 721 patients were screened, of which 452 (63%, 95%CI 59-66) were eligible, and 166 were approached to take part in the trial (37% of eligible, 95%CI 32-41). Fifty-two patients were randomised (Figure 1) (consent rate (of approached) = 31%, 95%CI 24-39; consent rate (of eligible) = 12%, 95%CI 9-15), representing 79% of the recruitment target sample size (recruitment rate (of approached) = 31%, 95%CI 24-39; recruitment rate (of screened) = 7%, 95%CI 5-9; average monthly recruitment rate = 4.3). The minimisation algorithm provided appropriate balance over 2-year risk of progression to kidney failure, however an error in the algorithm led to an imbalance in patient-reported ethnicity between groups. All participants self-reported as regular computer users.

Average follow up was 8.0 months (SD 3.8). In total, n=2 patients withdrew from the trial during follow up after moving geographical region (both withdrew from the intervention and 1 from all follow-up) (retention = 96%, 95%CI 87-100). During the study, n=17 patients met the trial definition of kidney failure (the study protocol mandated exit at this point) and there was n=1 death. No patients were excluded from the analysis. Case report form return rates were excellent throughout (99.5% of all expected forms received) (Supplementary Appendix, Table S1).

[Figure 1 near here]

The main reason for non-approach of screened and eligible individuals was that patients had not registered to use the existing hospital patient portal 'MyHealth' (90% of those not approached). For patients that were approached, but who were not willing to take part, reported reasons included: 'no internet access/computer inexperienced' (45%); 'not interested in research' (22%); 'too burdensome (completing ePROMs)' (11%); 'too burdensome (general)' (11%); 'issues with myHealth patient portal sign-up' (9%); 'unwell/health-related reasons' (2%); 'too burdensome (travel/trial visits)' (2%).

The average age of participants was 57 years (range 25-86), 29% were female, 37% reported 'non-white' ethnicity, 96% reported secondary level education or greater and 100% reported regular use of a computer, tablet or smartphone at least weekly. Mean baseline eGFR was 15.2, the average 2-year Tangri risk of progression to kidney failure was 43%, and the average EQ-5D index was 0.74 (Table 1).

For peer review only

Table 1. Baseline characteristics.

		Monthly ePROM reports (N = 24)	Usual care (N = 28)	Overall (N = 52)
**Minimisation variables**				
Risk progression	<40%	11 (46%)	14 (50%)	25 (48%)
	≥40%	13 (54%)	14 (50%)	27 (52%)
Self-reported computer experience*	'Yes'	24 (100%)	28 (100%)	52 (100%)
	'No'	0 (0%)	0 (0%)	0 (0%)
Ethnicity	'white'	18 (75%)	15 (54%)	33 (63%)
	'non-white'	6 (25%)	13 (46%)	19 (37%)
**Demographic and other baseline variables**				
Age, years	Mean (95% CI)	58 (51-65)	56 (50-61)	57 (52-61)
Gender	Female	7 (29%)	8 (29%)	15 (29%)
	Male	17 (71%)	20 (71%)	37 (71%)
Highest level of education	Higher education (e.g., Bachelors/ Masters/Professional degree/ PhD)	9 (38%)	9 (32%)	18 (35%)
	Further education (e.g., A-Levels / Vocational training)	9 (38%)	7 (25%)	16 (31%)
	Secondary education (e.g., GCSEs/O-levels)	6 (25%)	10 (36%)	16 (31%)
	Primary education	0 (0%)	0 (0%)	0 (0%)
	No qualifications	0 (0%)	2 (7%)	2 (4%)
	Not known	0 (0%)	0 (0%)	0 (0%)
Baseline medical history	Hypertension	17 (71%)	25 (89%)	42 (81%)
	Atrial Fibrillation	1 (4%)	1 (4%)	2 (4%)
	Ischaemic Heart Disease	2 (8%)	4 (14%)	6 (12%)
	Peripheral Vascular Disease	0 (0%)	3 (11%)	3 (6%)
	Diabetes (Type I)	2 (8%)	4 (14%)	6 (12%)
	Diabetes (Type II)	7 (29%)	8 (29%)	15 (29%)
	Cerebrovascular Disease	0 (0%)	0 (0%)	0 (0%)
	Chronic Respiratory Disorder	2 (8%)	2 (7%)	4 (8%)
	Thyroid Disease	0 (0%)	0 (0%)	0 (0%)
	Rheumatoid Arthritis	0 (0%)	1 (4%)	1 (2%)

For peer review only - http://bmjopen.bmj.com/site/about/guidelines.xhtml

	Anxiety/Depression	0 (0%)	2 (7%)	2 (4%)
	Cancer	6 (25%)	1 (4%)	7 (13%)
Systolic BP (mmHg)	Mean (95% CI)	147.6 (139.1-156.0)	146.0 (139.9-152.1)	146.8 (141.7-151.8)
Diastolic BP (mmHg)	Mean (95% CI)	78.8 (75.2-82.4)	77.4 (72.9-81.8)	78.0 (75.2-80.9)
Health-Related Quality of Life (EQ-5D-5L index)	Mean (95% CI)	0.70 (0.60-0.80)	0.78 (0.71-0.85)	0.74 (0.68-0.80)
2-year Tangri[1] risk of progression to kidney failure	Mean (95% CI)	0.48 (0.40-0.57)	0.43 (0.34-0.51)	0.45 (0.39-0.51)
eGFR (mL/min/1,73 m^2)	Mean (95% CI)	14.0 (12.5-15.6)	15.7 (13.9-17.5)	14.9 (13.7-16.1)
Creatinine (µmol/L)	Mean (95% CI)	384.0 (345.8-422.2)	357.5 (316.3-398.8)	369.8 (341.4-398.1)
Calcium (µmol/L)	Mean (95% CI)	2.2 (2.2-2.3)	2.3 (2.2-2.3)	2.3 (2.2-2.3)
Bicarbonate (µmol/L)	Mean (95% CI)	20.8 (19.8-21.9)	21.3 (20.3-22.2)	21.1 (20.4-21.7)
Phosphate (µmol/L)	Mean (95% CI)	1.4 (1.3-1.5)	1.4 (1.3-1.5)	1.4 (1.3-1.5)
Albumin (g/L)	Mean (95% CI)	40.4 (38.2-42.6)	40.8 (39.0-42.7)	40.6 (39.2-42.0)
ACR (mg/mmol)	Median (IQR)	206.1 (126.9-285.2)	178.1 (109.7-246.4)	191.0 (139.5-242.5)
Blood Glucose (mmol/L)#	Mean (95% CI)	8.4 (6.8-9.9)	7.0 (5.6-8.4)	7.6 (6.5-8.6)
	Missing	1 (2%)	1 (2%)	2 (4%)
HbA1c (mmol/mol)#	Mean (95% CI)	57.2 (42.8-71.6)	53.2 (44.0-62.5)	54.6 (47.1-62.2)
	Missing	4 (8%)	3 (6%)	7 (14%)

*defined as regular use of a computer, tablet or smartphone at least weekly; #for diabetic participants. [1] Tangri N, Stevens LA, Griffith J, et al. A predictive model for progression of chronic kidney disease to kidney failure. Jama. 2011;305(15):1553-1559.[21] Electronic patient-reported Outcome, ePROM; Inter Quartile Range, IQR; blood pressure, BP; EuroQol five-level five-dimension PRO, EQ5D-5L; estimated Glomerular Filtration Rate, eGFR; Albumin Creatinine Ratio, ACR; glycated haemoglobin, HbA1c.

For peer review only

ePROM Intervention adherence and reporting patterns

Overall, 73% (95%CI 67-79) of expected ePROM questionnaires were received during the trial (Table 2). However, only 31% (95%CI 25-37) were received within our *a priori* agreed compliance window (72-hours either side of the scheduled reminder date). Patients submitted 98 'ad-hoc' questionnaires outside of this compliance window: an average of 4 per participant. Compliance over time was good, with a high proportion of participants submitting at least one scheduled questionnaire beyond 90 days post-randomisation (74%, 95%CI 52-90) and after 180 days (65%, 95%CI 41-65) but this proportion dropped beyond 270 days (46%, 95%CI 19-75).

For peer review only

Table 2. ePROM compliance.

Total number of expected ePROM questionnaires*	Total received (%, 95%CI))	Total number submitted in compliance window** (%, 95%CI)	Total number of ad-hoc ePROM questionnaire submissions	Mean number of ad-hoc submissions per patient	Number of patients on trial >90 days	Proportion of patients submitting ePROM questionnaires >90 days (95%CI)	Number of patients on trial >180 days	Proportion of patients submitting ePROM questionnaires >180 days (95%CI)	Number of patients on trial >270 days	Proportion of patients submitting ePROM questionnaires >270 days (95%CI)
230	169 (73, 67-79)	71 (31, 25-37)	98	4	23	74% (52-90)	20	65% (41-85)	13	46% (19-75)

*accounting for questionnaire allocation date and loss to follow-up/withdrawals/death/progression to kidney failure; **questionnaires received within a +/- 72-hour time window. electronic patient-reported Outcome, ePROM.

For peer review only

Patients reported 579 symptoms, the most prevalent of which included fatigue, shortness of breath, itchy/dry skin and pain (Table 3, n=20 patients reported symptoms during the trial, n=4 did not report any symptoms). Most symptoms reported were mild (60%). There were 16 severe and current symptom reports (across 13 questionnaires), generated by 5 patients, representing 3% of the total number of symptoms reported across the trial (for full details around system notifications see Supplementary Appendix, Tables S2, S3 and S4). The symptoms driving these notifications were itchy/dry skin (37% of notifications), fatigue (25%), shortness of breath (13%), pain (13%), difficulty sleeping (6%) and ankle swelling (6%). The median time taken by staff to resolve patient notifications was 10 minutes (IQR 6.5-22.5) and actions included: 'telephone counselling about symptom management' (78%); and 'brought clinic appointment forwards' (22%); 'imaging/test orders' (22%); 'medication initiation/change' (11%); 'other' (11%), where more than one type of action could be recorded for each notification (see Supplementary Appendix Table S4).

For peer review only

Table 3. ePROM intervention: reporting pattern by symptom.

		Number of symptoms reported			
	Number of times reported	Mild (%)	Moderate (%)	Severe (%)	Proportion of total symptoms reported (N = 579)
Fatigue	135	69 (51)	60 (44)	6 (4)	23%
Shortness of breath	109	88 (81)	17 (16)	4 (4)	19%
Itchy/dry skin	102	53 (52)	42 (41)	7 (7)	18%
Pain	87	54 (62)	29 (33)	4 (5)	15%
Lack of appetite	57	35 (61)	22 (39)	0 (0)	10%
Ankle swelling	21	11 (52)	9 (43)	1 (5)	4%
Nausea	20	13 (65)	7 (35)	0 (0)	3%
Difficulty sleeping	17	7 (41)	9 (53)	1 (6)	3%
Faintness/dizziness	11	6 (55)	5 (45)	0 (0)	2%
Restless legs or difficulty keeping legs still	10	7 (70)	3 (30)	0 (0)	2%
Diarrhoea	10	5 (50)	5 (50)	0 (0)	2%
Problems with fistula	0	0 (0)	0 (0)	0 (0)	0%
TOTALS	579	348 (60)	208 (36)	23 (4)	

Electronic patient-reported outcome, ePROM.

For peer review only

Clinical and patient-reported outcomes

At 12 months, eGFR was higher (14.13, 95%CI 12.14-16.12, versus 12.71, 95%CI 10.78-14.64, adjusted mean difference 1.72, 95% CI -0.96-4.40), but the EQ-5D index was lower (0.59, 95%CI 0.34-0.85, versus 0.71, 95%CI 0.61-0.82, adjusted mean difference -0.04 95%CI -0.17-0.09). At 12 months, Tangri 2-year risk of progression to kidney failure was lower in the intervention arm than in the usual care arm (0.46, 95%CI 0.29-0.63, versus 0.52 95%CI 0.38-0.66), however after adjusting for minimisation factors and baseline Tangri risk score, the adjusted mean difference was 0.01 95%CI -0.21-0.22 (Table 4). Clinical event rates were similar between arms (Table 5). As expected, there were high levels of uncertainty around all point estimates given the limited size of the sample.

For peer review only

Table 4. Numeric outcome measures by trial arm and data collection point.

	Monthly ePROM reports (N = 24)		Usual care (N = 28)		
	No. (expected)	Mean (95% CI)	No. (expected)	Mean (95% CI)	Adjusted Mean Difference (95% CI)
Systolic BP (mmHg)					
Baseline	24 (24)	147.58 (139.12-156.05)	28 (28)	146.04 (139.94-152.13)	0.72 (-9.51 to 10.95)
3 months	21 (21)	145.14 (138.81-151.48)	26 (26)	140.46 (134.33-146.59)	0.13 (-7.50 to 7.76)
6 months	18 (18)	147.50 (141.92-153.08)	23 (23)	140.17 (132.33-148.02)	2.76 (-6.27 to 11.79)
9 months	11 (12)	141.91 (134.63-149.19)	16 (17)	142.19 (135.14-149.23)	-5.46 (-13.10 to 2.17)
12 months	7 (7)	148.71 (142.25-155.18)	10 (11)	137.70 (126.65-148.75)	7.87 (-5.47 to 21.20)
Diastolic BP (mmHg)					
Baseline	24 (24)	78.83 (75.22-82.45)	28 (28)	77.36 (72.94-81.77)	3.32 (-2.09 to 8.72)
3 months	21 (21)	78.81 (74.72-82.90)	26 (26)	72.85 (68.69-77.01)	4.38 (-0.40 to 9.16)
6 months	18 (18)	76.94 (70.94-82.95)	23 (23)	74.04 (69.66-78.43)	1.32 (-4.87 to 7.52)
9 months	11 (12)	78.00 (70.36-85.64)	16 (17)	78.44 (71.98-84.90)	-0.77 (-9.03 to 7.50)
12 months	7 (7)	79.00 (69.04-88.96)	10 (11)	76.90 (70.44-83.36)	0.24 (-8.92 to 9.40)
Health-Related Quality of Life (EQ-5D-5L index)					
Baseline	24 (24)	0.70 (0.60-0.80)	28 (28)	0.78 (0.71-0.85)	-0.06 (-0.17 to 0.06)
3 months	20 (21)	0.67 (0.53-0.80)	24 (26)	0.76 (0.69-0.84)	-0.03 (-0.13 to 0.07)
6 months	18 (18)	0.66 (0.52-0.80)	23 (23)	0.74 (0.65-0.82)	-0.00 (-0.11 to 0.10)
9 months	12 (12)	0.55 (0.33-0.78)	17 (17)	0.74 (0.66-0.82)	-0.07 (-0.24 to 0.09)

For peer review only

12 months	7 (7)	0.59 (0.34-0.85)	11 (11)	0.71 (0.61-0.82)	-0.04 (-0.17 to 0.09)
2-year Tangri[1] risk of progression to kidney failure (%)					
Baseline	24 (24)	0.48 (0.40-0.57)	28 (28)	0.43 (0.34-0.51)	0.06 (-0.01 to 0.14)
3 months	21 (21)	0.46 (0.38-0.54)	26 (26)	0.47 (0.38-0.55)	-0.01 (-0.10 to 0.08)
6 months	16 (18)	0.45 (0.34-0.57)	22 (23)	0.43 (0.35-0.52)	-0.01 (-0.12 to 0.10)
9 months	11 (12)	0.46 (0.34-0.58)	16 (17)	0.50 (0.41-0.58)	-0.04 (-0.16 to 0.08)
12 months	5 (7)	0.46 (0.29-0.63)	10 (11)	0.52 (0.38-0.66)	0.01 (-0.21 to 0.22)
eGFR (mL/min/1,73 m2)					
Baseline	24 (24)	14.03 (12.52-15.55)	28 (28)	15.70 (13.93-17.47)	-1.86 (-4.18 to 0.46)
3 months	21 (21)	13.51 (11.89-15.12)	26 (26)	14.07 (12.22-15.91)	0.94 (-0.73 to 2.61)
6 months	18 (18)	13.11 (10.93-15.29)	23 (23)	14.19 (12.49-15.89)	0.28 (-1.86 to 2.43)
9 months	11 (12)	14.54 (12.38-16.70)	16 (17)	13.13 (11.35-14.92)	2.46 (0.30 to 4.63)
12 months	7 (7)	14.13 (12.14-16.12)	10 (11)	12.71 (10.78-14.64)	1.72 (-0.96 to 4.40)
Creatinine (µmol/L)					
Baseline	24 (24)	384.00 (345.84-422.16)	28 (28)	357.54 (316.29-398.78)	39.42 (-9.71 to 88.54)
3 months	21 (21)	380.81 (346.19-415.43)	26 (26)	396.08 (342.23-449.92)	-34.81 (-66.83 to -2.79)
6 months	18 (18)	408.39 (359.35-457.43)	23 (23)	375.96 (334.91-417.00)	-17.82 (-57.55 to 21.92)
9 months	11 (12)	364.45 (305.24-423.67)	16 (17)	399.50 (347.47-451.53)	-41.90 (-88.94 to 5.13)
12 months	7 (7)	370.00 (306.19-433.81)	10 (11)	409.10 (337.29-480.91)	-47.60 (-131.55 to 36.36)
Calcium (µmol/L)					
Baseline	24 (24)	2.24 (2.19-2.29)	28 (28)	2.27 (2.25-2.30)	-0.03 (-0.09 to 0.02)
3 months	21 (21)	2.28 (2.22-2.35)	26 (26)	2.29 (2.24-2.34)	0.02 (-0.04 to 0.08)
6 months	18 (18)	2.30 (2.25-2.35)	23 (23)	2.34 (2.29-2.39)	-0.01 (-0.07 to 0.04)

For peer review only

9 months	11 (12)	2.37 (2.27-2.47)	16 (17)	2.40 (2.35-2.46)	-0.03 (-0.11 to 0.04)
12 months	6 (7)	2.40 (2.35-2.45)	10 (11)	2.40 (2.29-2.50)	0.01 (-0.08 to 0.10)
Bicarbonate (μmol/L)					
Baseline	24 (24)	20.83 (19.76-21.89)	28 (28)	21.25 (20.33-22.17)	-0.30 (-1.70 to 1.09)
3 months	21 (21)	21.36 (20.13-22.59)	25 (26)	21.30 (20.16-22.45)	0.19 (-1.26 to 1.64)
6 months	17 (18)	20.56 (19.14-21.99)	21 (23)	21.19 (19.97-22.41)	0.49 (-0.92 to 1.91)
9 months	11 (12)	21.82 (19.59-24.04)	15 (17)	20.73 (19.14-22.33)	1.13 (-1.32 to 3.59)
12 months	5 (7)	21.60 (18.93-24.27)	9 (11)	20.67 (17.76-23.57)	1.03 (-2.44 to 4.50)
Phosphate (μmol/L)					
Baseline	24 (24)	1.41 (1.31-1.52)	28 (28)	1.40 (1.30-1.51)	0.01 (-0.14 to 0.16)
3 months	21 (21)	1.47 (1.39-1.55)	25 (26)	1.60 (1.41-1.79)	-0.14 (-0.34 to 0.05)
6 months	17 (18)	1.52 (1.36-1.69)	21 (23)	1.38 (1.23-1.52)	0.06 (-0.12 to 0.25)
9 months	11 (12)	1.45 (1.27-1.62)	14 (17)	1.46 (1.30-1.61)	-0.03 (-0.26 to 0.21)
12 months	5 (7)	1.61 (1.28-1.93)	9 (11)	1.42 (1.25-1.60)	0.31 (0.02 to 0.59)
Albumin (g/L)					
Baseline	24 (24)	40.38 (38.20-42.55)	28 (28)	40.82 (38.98-42.66)	-0.52 (-3.34 to 2.30)
3 months	21 (21)	39.43 (37.50-41.36)	26 (26)	39.58 (37.80-41.36)	0.91 (-0.61 to 2.43)
6 months	18 (18)	37.39 (35.15-39.62)	23 (23)	37.65 (35.90-39.41)	0.24 (-1.56 to 2.04)
9 months	11 (12)	35.27 (33.12-37.42)	16 (17)	36.50 (34.52-38.48)	0.37 (-2.31 to 3.05)
12 months	7 (7)	36.86 (34.42-39.29)	10 (11)	35.10 (32.90-37.30)	1.63 (-1.38 to 4.64)
ACR (mg/mmol)					
Baseline	24 (24)	206.06 (126.92-285.20)	28 (28)	178.08 (109.73-246.43)	23.64 (-66.09 to 113.37)
3 months	21 (21)	167.31 (101.53-233.09)	26 (26)	149.25 (108.39-190.11)	-19.60 (-63.75 to 24.56)

For peer review only - http://bmjopen.bmj.com/site/about/guidelines.xhtml

6 months	16 (18)	182.24 (95.65-268.83)	22 (23)	135.88 (88.78-182.98)	-3.73 (-72.53 to 65.07)
9 months	11 (12)	227.58 (117.37-337.79)	16 (17)	148.23 (97.56-198.90)	0.20 (-84.56 to 84.96)
12 months	5 (7)	175.74 (97.71-253.77)	10 (11)	161.51 (74.67-248.35)	-14.40 (-138.43 to 109.63)
Blood Glucose (mmol/L)					
Baseline	8 (9)	8.36 (6.82-9.90)	11 (12)	6.97 (5.58-8.36)	1.48 (-0.57 to 3.52)
3 months	7 (9)	9.36 (5.39-13.33)	8 (11)	8.74 (5.80-11.68)	-2.18 (-6.22 to 1.87)
6 months	5 (8)	15.88 (3.47-28.29)	5 (10)	7.22 (5.14-9.30)	-2.58 (-13.52 to 8.36)
9 months	4 (6)	8.93 (5.36-12.49)	3 (8)	6.30 (3.84-8.76)	2.12 (-1.40 to 5.64)
12 months	1 (4)	10.70#	2 (5)	5.10 (1.57-8.63)	-
HbA1c (mmol/mol)					
Baseline	5 (9)	57.20 (42.83-71.57)	9 (12)	53.22 (43.98-62.46)	3.18 (-12.52 to 18.87)
3 months	7 (9)	53.29 (43.78-62.79)	7 (11)	46.14 (38.80-53.48)	2.36 (-4.61 to 9.33)
6 months	7 (8)	51.14 (44.40-57.88)	8 (10)	50.63 (40.45-60.80)	-6.00 (-14.06 to 2.05)
9 months	2 (6)	59.50 (52.64-66.36)	3 (8)	52.67 (43.04-62.29)	-
12 months	2 (4)	57.00 (51.12-62.88)	3 (5)	49.33 (36.87-61.80)	-6.58 (-9.21 to -3.96)

#Insufficient data to calculate 95% CI. [1] Tangri N, Stevens LA, Griffith J, et al. A predictive model for progression of chronic kidney disease to kidney failure. Jama. 2011;305(15):1553-1559.[21] Electronic Patient-Reported Outcome Measure, ePROM; blood pressure, BP; EuroQol five-level five-dimension PRO, EQ5D-5L; Estimated Glomerular Filtration Rate, eGFR; Albumin Creatinine Ratio, ACR; glycated haemoglobin, HbA1c.

For peer review only

Table 5. Binary outcome measures by trial arm and data collection point.

	Monthly ePROM reports (N = 24)		Usual care (N = 28)		
	N[a]	Events (%, 95% CI)	N[a]	Events (%, 95% CI)	Risk Ratio (95% CI)[b]
**Death**					
Baseline to 3 months	24	0 (0, 0-14)	28	0 (0, 0-12)	-
3 to 6 months	21	1 (5, 0-24)	26	0 (0, 0-13)	-
6 to 9 months	18	0 (0, 0-19)	23	0 (0, 0-15)	-
9 to 12 months	12	0 (0, 0-26)	17	0 (0, 0-20)	-
Total		1 (4, 0-21)		0 (0, 0-12)	-
**Kidney failure**					
Baseline to 3 months	24	1 (4, 0-21)	28	4 (14, 4-33)	0.29 (0.30 to 2.44)
3 to 6 months	21	3 (14, 3-36)	26	2 (8, 1-25)	1.86 (0.34 to 10.11)
6 to 9 months	18	3 (17, 4-41)	23	0 (0, 0-15)	-
9 to 12 months	12	1 (8, 0-38)	17	3 (18, 4-43)	0.47 (0.06, 4.01)
Total		8 (33, 16-55)		9 (32, 16-52)	1.04 (0.47 to 2.26)
**Hospitalisation**					
Baseline to 3 months	24	1 (4, 0-12)	28	1 (4, 0-18)	1.17 (0.08 to 17.67)
3 to 6 months	21	2 (10, 1-30)	26	3 (6, 2-30)	0.83 (0.15 to 4.49)
6 to 9 months	19	2 (11, 1-33)	23	2 (9, 1-28)	1.21 (0.19 to 7.80)
9 to 12 months	12	0 (0, 0-26)	17	0 (0, 0-20)	-
Total		5 (21, 7-42)		5[c] (18, 6-37)	1.17 (0.38 to 3.55)

Electronic patient-reported outcome, ePROM. [a]Number of participants in the study at start of timepoint.
[b]unadjusted risk ratios are reported due to the low frequencies of events. [c]This figure denotes the number of unique individuals with at least one hospital stay during the study. Individuals can have more than one hospital stay.

For peer review only - http://bmjopen.bmj.com/site/about/guidelines.xhtml

Healthcare utilisation

Patients in the intervention arm reported 97 fewer episodes of healthcare utilisation than those in the usual care arm (Table 6) (mean number of episodes per patient: intervention arm = 10.3, usual care arm = 12.3; intervention arm 0.11 fewer mean episodes per month on trial), which included 54 fewer CKD-related specialist kidney clinic visits (mean per patient: intervention arm = 5.4, usual care arm = 6.5; intervention arm 0.07 fewer episodes per month on trial). Hospital inpatient stay was similar in both arms. Again, this exploratory data should be treated with caution owing to the small sample size.

For peer review only

For peer review only - http://bmjopen.bmj.com/site/about/guidelines.xhtml

Table 6. Summary of healthcare utilisation.

	CKD-Related				Not CKD-Related				CKD relationship unknown			
	Intervention (N = 24)		Usual care (N = 28)		Intervention (N = 24)		Usual care (N = 28)		Intervention (N = 24)		Usual care (N = 28)	
NHS service category	Episodes	NHS hospital inpatient stay (days)	Episodes	NHS hospital inpatient stay (days)	Episodes	NHS hospital inpatient stay (days)	Episodes	NHS hospital inpatient stay (days)	Episodes	NHS hospital inpatient stay (days)	Episodes	NHS hospital inpatient stay (days)
GP appointment	1		4 (n=2)		14 (n=9)		23 (n=15)		0		4 (n=2)	
GP out of hours service	0		0		0		1		0		0	
Specialist kidney clinic	129 (n=22)		183 (n=26)		1		0		0		0	
NHS outpatient clinic (other than specialist kidney clinic)	10 (n=6)		15 (n=12)		41 (n=13)		74 (n=17)		1		1	
NHS walk-in centre	0		0		1		0		0		0	
NHS 111/NHS direct telephone call	0		0		1		1		0		0	
A&E	1		0		2 (n=2)		5 (n=3)		1		1	
NHS hospital inpatient stay	4 (n=3)	7	2 (n=2)	2	2 (n=2)	7	2 (n=2)	8	0		2 (n=2)	2
Other:	9 (n=5)		19 (n=13)		27 (n=4)		8 (n=3)		2 (n=2)		0	
Imaging	3		6		2		1		1		0	
Home visit	2		5		0		0		0		0	
Phlebotomy	1		1		0		0		0		0	
Health education/roadshow/open day	1		1		0		0		0		0	
Chemotherapy	0		0		8		0		0		0	
Ophthalmology procedure	0		0		1		0		0		0	
Other (NHS)	2		5		1		7		1		0	
Other (private)	0		0		15		0		0		0	
TOTALS	154 (n=22)	7	222 (n=26)		89 (n=14)		114 (n=23)		4 (n=2)		8 (n=4)	

General practice, GP; National Health Service, NHS; Accident and Emergency, A&E.

For peer review only

Safety, protocol deviations

There was 1 serious adverse event (n=1 death) reported during the trial. Two protocol deviations were recorded, 1 software error (resolved) and 1 informed consent form error (missing initial) (Supplementary Appendix, Table S5).

For peer review only

Qualitative sub-study

Semi-structured interviews were conducted with 24 trial participants (intervention arm n=14; usual care arm n=10) and 1 HCP. Interviewee responses supported proof of concept and acceptability and indicated that the system had met our four-fold remit[20]:

1. To allow patients with advanced CKD to remotely self-report their symptoms using a simple and secure online platform.
2. To provide appropriate self-management advice to patients whose ePROM scores highlighted one or more mild/moderate/severe symptoms.
3. To allow monitoring of real-time patient ePROM symptom data and subsequent automated notification of both the patient and the clinical team in the advent of a severe symptom.
4. To incorporate longitudinal ePROM symptom data in the electronic patient record to help inform clinical consultations and support shared understanding/decision-making.

A summary of qualitative findings regarding intervention positives/negatives and suggested system changes is presented in Table 7. Patients highlighted benefits around login security; questionnaire structure, clarity and coverage; and felt reassurance that their questionnaire data, including their free text comments (Supplementary Appendix Table S6), were being monitored and responded to promptly and/or discussed in clinic. They also reported that the advice around symptoms and self-management was useful and helped alleviate anxiety around the symptoms they were experiencing. The HCP reported that the system provided a useful tool to guide the consultation, allowing more time for the discussion of patient-important issues, and felt that it might be particularly beneficial in supporting the widespread remote follow up implemented in the UK in response to the COVID-19 pandemic.

The main system shortfalls, identified across the whole sample, included: failures of the reminder process meaning some patients did not receive reminder emails; a lack of clarity for some patients around which questionnaire they should complete at which timepoint and confusion around how to view self-management advice; difficulty navigating/scrolling through sections; occasional problems for some patients when submitting the questionnaire; and the need for clinicians to open up a separate system within the electronic patient record to access the ePROM intervention, thus preventing use in busy multidisciplinary team meetings. Interviewees suggested a range of changes aimed at addressing these shortfalls and enhancing the overall functionality of the intervention.

For peer review only

Table 7. Summary of qualitative findings regarding intervention positives/negatives and suggested system changes.

Theme	Subtheme	Illustrative quote
Intervention positives	Questionnaire data picked up by care team and acted upon	"On a few occasions I was very impressed that what I had put on the form, obviously had been noticed and had been picked up. And was discussed with me at clinic and I thought that was one of the big positives of the form itself." [Patient 01] "I would always start the consultation with thank you for taking part, I've been looking at this, shall we look at it together, I see that here you reported this, would you like to tell me a bit about that… patients… seemed really pleased that we were looking at it and using it and it was meaningful. Because clearly it was something that they were taking time and trouble to do. And so, for them knowing that we were using it and taking it seriously was probably a really good thing." [HCP 01]
	Provided reassurance when symptoms 'normal'	"…it does give you some reassurance if you can be told, well that's normal for the problems you've got." [Patient 02]
	Quick to complete	"The first one probably took me quarter of an hour because I read through it very carefully and double checked what I was saying as I went along. But once I'd done a couple then it was sort of less than ten minutes… I sort of answered the questions as I felt at the time… But it was a breeze once I got used to it that was fine it was easy to fill in." [Patient 03]
	Alleviated anxiety	"I found it positive. I think it takes worries away to be honest with you… You have the advice that was given, so you didn't feel as if you're the only person that ever-had itchiness before. It was obviously something that was very common. So, I would have said it alleviated any anxiety, for me." [Patient 01]
	Questionnaire structure/Questions clear/Questions appropriate	"I think the questions, they're quite clear and quite precise." [Patient 04]; "…my symptoms was more headaches, itchy skin, swelling which it covered, tiredness which it covered… I think it covered everything from my point of view." [Patient 05]
	System provided guidance around when it was appropriate to contact the clinical team	"Just sort of prompted you to, if the symptoms were a bit… it prompted you to give the QE a ring and discuss it, you know what I mean… you know like feeling worse and feeling tired or whatever, just to ring up and speak to somebody cause sometimes you don't… you just don't do that… you just carry on, you just carry on till your next appointment. So, it made you think about it." [Patient 06]

For peer review only - http://bmjopen.bmj.com/site/about/guidelines.xhtml

	Immediate clinical assistance available upon questionnaire submission	"…it's nice to know that, you know… if anything is going wrong then I can get help more or less straightaway." [Patient 07]
	Used free-text comments to communicate with nursing staff	"Initially I was filling the form in and putting very little additional information on. Latterly I was putting a lot more information on and I was very pleased on two occasions that when I went for my renal check-up, the points that I'd made had been noticed and were brought up… it was an additional form of communication in that if I'd got a concern or something was happening, I could put it on the form… and you could use it to answer questions then as to how you were coping, what you were doing and how you were feeling." [Patient 01]; "I think that was the good thing about the free text because it did allow people to tell us things that we hadn't particularly asked about." [HCP 01]
	Provided self-management advice	"…very useful because as a lay person not understanding the functions of the body, not that well if you see what I mean, it's useful sometimes to get a bit of guidance as to where you need to go." [Patient 03]
	Login security	"…I think the security of, if you like, the double tier I think is very, very good indeed." [Patient 08]
	System simple to use/user-friendly	"I think it's quite simple and user friendly." [Patient 04]
	Useful tool to guide consultation	"It was a nice tool to guide consultation. So normally you've just got your clinic letter from your previous visit, and that gives you a fair idea of the kind of things that you're going to talk to the patient about based on the things that you've talked to them about before and the active medicine which you've identified. But having the RePROM as well often highlighted things that were completely off the radar. And I think it's perfectly likely the patient would have mentioned it themselves anyway, it meant that you knew in advance and you were able to get straight into it, rather than it being the kind of thing that they casually mention as they're leaving the room. So, you have a bit more time to explore things in a bit more detail I think." [HCP 01]
	Would allow remote follow up post-COVD	"…now our capacity to see patients face-to-face has reduced by about 75% because of the need for social distancing. So actually, now that they're almost all phone and video consultations something like RePROM is more important than ever because that does give patients a bit more of an ability to… to contact us and tell us things that they were worried about in between their reviews." [HCP 01]

For peer review only

Intervention negatives	Not receiving reminders	"…some of the time it didn't come through on my daughter's iPhone and then it would come through the next month but miss a month… Seemed to be hit and miss sometimes." [Patient 07]
	Confusion around which questionnaire to complete	"The complicated bit, which I did struggle with, was trying to get up the latest questionnaire, which needed to be completed…" [Patient 1461]; "I would actually number the questionnaires so you can tell which ones you've done and completed… sometimes I didn't know which ones I'd done and which ones I hadn't done…" [Patient 05]
	Prominence of next steps and self-management advice	"Yeah, I don't remember seeing too much of that [information] at the end of it to be honest." [Patient 1156]
	Difficulty navigating through multiple sections within the system	"…actually, sort of navigating your way through the electronic system, that all could be made a bit easier." [Patient 08]; "When I first started with the system probably about the first three, maybe four months, everything was fine. Then for some reason one of the sections within a section, if you understand what I mean, you've got the outer bar would work so I could scroll down but the inner bar I couldn't scroll down completely and you'd, there were like 10 questions, maybe 12 questions, and you could get down to question eight, but I couldn't get down to the last two or four, whichever it was…" [Patient 09]
	Difficulty submitting the questionnaire	"…on two separate occasions we did try and fill it out but then the problem is there was never a finish or a continuation of the questionnaire, so we couldn't exactly finish it..." [Patient 10]
	Need to open up a different system precluded use in Multidisciplinary Team (MDT) meetings	"We had lots of great ideas at the beginning about how we'd look at it and the MDT when we looked forward to the next clinic but actually the MDT's are so busy and there were so many people to get through that it just a quick, look at the blurb, what are the outstanding issues, move on. And so, we didn't use it because that would have meant getting the Portal up rather than just PICS and waiting for it to load and so no, we didn't use it in the MDT." [HCP 01]
Intervention acceptability	Patient acceptance of remote follow up/ability to engage with technology	"I guess COVID has taught us a couple of things. The first thing is that we've all said, a lot of people have said, oh patients won't cope with phone consultations, and they certainly won't cope with video consultations. Patients are not very tech savvy, they won't be able to do it, they're all very elderly, a lot of them don't speak any English and it would be a complete disaster. And that's not completely been

		our experience, people seem to have adapted to phone consultations and video consultations really quite well." [HCP 01]
Suggested changes to intervention system	Improve reminders	"…perhaps like my daughter found that, you know, it was hit and miss when the questionnaire [reminders] came through. That could be improved on…" [Patient 07]
	Enhance/simplify interface	"The only thing I can think of as far as improving the system is to make it more user-friendly basically… navigating your way through the electronic system… could be made a bit easier." [Patient 08]; "I think the practical obstacle… was that patients find the interface difficult." [HCP 01]
	Incorporate dietary advice	"…my major one really, which I've been surprised at, was the lack of information regarding, you know, diet…" [Patient 11]
	Incorporate questions around psychological wellbeing/mood	"I think just having that questionnaire to see how your mood is and how you can look back on it and see where, like, how you can improve and how you can change it slightly and try and move on from there…" [Patient 10]; "I'm not particularly surprised that people mentioned that [anxiety & depression], and I think that's reasonable. I think in a future iteration we probably should try and capture that." [HCP 01]
	Consider timing of questionnaire completion in relation to clinical encounter/receiving results	"I'm getting the results sometimes before I answer the questionnaire, and I think that possibly can end in user bias 'cause if my results are not very good then sometimes that can translate into feeling bad, you know, rather than the other way round, if you know what I mean?" [Patient 12]
	Incorporate other symptom questions	"I think its worthwhile [adding]… leg cramps… it's just when you're in bed at night and lying down. It'll be like absolutely agonising, just like really painful… it is one of the key symptoms, yeah." [Patient 04]
	Immediate display of self-management advice as each question is answered in addition to the end-of-questionnaire summary	"…or maybe even both, straight away and at the end when it produces a report of your answers. I say have both options really…" [Patient 04]
	Add a tick-box option to prompt contact with the clinical team	"I'd perhaps have the tick box at the end of the questions… to say 'could somebody ring you' would be a good idea… for someone to give you that reassurance with a phone call… of how to ease the symptoms." [Patient 05]

For peer review only - http://bmjopen.bmj.com/site/about/guidelines.xhtml

	Simplify the questionnaire submission process	"I found a little bit of confusion on the last page where you, they showed you your answers, what you'd put, there's submit button on that page. I had to come back a page to submit it, that caused confusion a couple of times." [Patient 01]
	Make data available to GPs	"…the GP side of things in the UK isn't necessarily that well linked into the hospital system… I just get the feeling that there isn't too much of an interface between the [hospital] and the GP unless the hospital contacts the GP… this cross-border thing doesn't really make a lot of sense to someone like myself, you know, with the technology that we have these days you'd think that it would be sensible to have the GP on if you like a version of 'MyHealth' so they can see exactly what the hospital are seeing, obviously within the rules of confidentiality… I think the more integrated it is the better it will work" [Patient 03]
	Combine questionnaire data with other clinical/lifestyle information collected at home	"…it was just my wondering whether there was another level perhaps… whether blood pressure something like that…things like the blood pressure and weight I have to record every day anyway…" [Patient 13]
	Consider flexibility in setting notification thresholds for different symptoms	"Have the same system as the failsafe system but don't have it as severe. Maybe say level three, make it to level two or level one." [Patient 14]
	Consider expanding use of the system to dialysis populations	"I definitely think that doing something like this in terms of the dialysis population would be massively useful… Compared to the very close supervision that they had in the year, six months before they started dialysis. A year to six months after they've started dialysis that is an entirely different experience… anecdotally a lot of patients say, oh gosh I used to come to clinic and see doctors and nurses and dieticians and now I'm at my satellite unit I see the nurses all the time and I occasionally see a dietician but it doesn't feel the same… I think they find that quite a worrying time, and maybe having something like this to support them particularly in that transition would be really useful." [HCP 01]
	Consider use of a central platform to aid roll out to other centres	"I think the difficulty when we think about rolling it out to other places is that everywhere will have a different electronic patient record type system… we'll have to think about how the IT works in each of those places..." [HCP 01]

For peer review only

For peer review only - http://bmjopen.bmj.com/site/about/guidelines.xhtml

DISCUSSION

In this single centre open-label randomised study, we examined the feasibility of randomising patients with advanced CKD to monthly ePROM reporting with real-time feedback of data or to usual care. We found that the majority of study indicators supported the feasibility of a full-scale RCT: patient eligibility rate (proportion of screened patients eligible) 63%; recruitment rate (of patients approached) 31%; case report form returns 99.5%; and retention 96%. In total, 52 patients were randomised (monthly recruitment rate = 4.3), representing 79% of the recruitment target sample size (N = 66). This level of recruitment would position the study in the top quartile of performance based on a review of recruitment and retention across 151 RCTs funded by the UK Health Technology Assessment Programme.[27] Moreover, overall adherence to the intervention was good, with patients returning 73% of expected ePROM questionnaires, although not always in the specified time windows. We have therefore demonstrated that it is possible to randomise and follow up patients with high levels of data completion up to 12 months, and that a RCT is feasible.

Within our study, we found the observed pattern of ePROM reporting did not correspond with our *a priori* expectations. Relatively few patients submitted their questionnaires within our pre-specified compliance window (72-hours either side of the scheduled submission date). Triangulation with qualitative data suggested that it was unlikely that this observation was related to issues around acceptability of the intervention: all participants indicated positive engagement with the system. Moreover, overall questionnaire return rates were high. A number of patients reported a failure to receive email reminders, or that emails were sent to junk folders, which may have contributed to out-of-window submissions: where patients relied on memory, rather than external prompts. Several patients suggested adding a mobile text reminder option, which they felt would be more reliable. It was our initial intention to include such an option, unfortunately, this was not possible within the existing patient portal framework. This feature will be made available as a priority within the next iteration of the system.

Our overall findings around feasibility align with similar research conducted in oncology. The feasibility of trial-based exploration of ePROM efficacy in this area has been well established and a number of trials successfully completed internationally, in the US[10], France[11] and in the UK.[28] Within kidney research, whilst the feasibility of routine collection of ePROMs in clinical practice has been supported[29 30], there has been relatively little research around trial feasibility until recently. The 'symptom monitoring with feedback trial' (SWIFT), is a registry-based pilot cluster randomised controlled trial among Australian and New Zealand adults with end-stage kidney disease managed on haemodialysis; due for completion in 2020/21.[31] Early findings from the pilot study suggest feasibility and acceptability when implementing ePROMs with feedback to clinicians in Australian haemodialysis centres, supporting progress to a follow-on multicentre RCT.[32]

Previous ePROM trials have commonly included a primary outcome based around health-related quality of life, for example, measured using the EQ-5D.[10] Based on our study population data, it would require a total of 348 participants to detect a clinically meaningful 0.07 reduction in EQ-5D-5L index[33] (SD=0.18, p=0.05, 90% power, adjusting for 20% attrition). This sample size appears achievable based on the successful implementation of previous UK-led kidney trials with similar (or greater) sample size requirements.[34 35]

Whilst the study intervention was well received by patients and demonstrated proof of concept, there were a number of suggested improvements that may enhance longer-term

For peer review only - http://bmjopen.bmj.com/site/about/guidelines.xhtml

engagement with the system, for example: simplification the interface and, in particular, improvements to the reminder functionality; incorporation of automated dietary advice; and the inclusion of additional questionnaire items around the psychological impacts associated with CKD. In addition, it was suggested that use of the intervention within a multi-centre trial may necessitate system-level modifications to ensure compatibility with different IT infrastructures at other hospitals. Work conducted within a UK oncology setting has shown that it is possible to integrate a single ePROM system across multiple NHS trusts, each with unique IT platforms, but that repeated integration at each separate site often takes considerable time and resources.[9] Our own experience of linking an ePROM to an existing hospital-based patient portal was mixed. Positives included the in-built security aspects, which some patients particularly valued, and also the ability to share data within the electronic medical record relatively easily. Negatives included functionality issues around the interface and the lack of some important features, e.g., text reminders and smartphone compatibility. In addition, issues with sign-up to the patient portal for some patients meant that study staff could not approach them to take part in the trial without first arranging access to the patient portal, which created a substantial barrier to recruitment.

Looking ahead to the roll-out of an ePROM system within a multicentre trial, and also considering future potential implementation in clinical practice, the use of a single hospital patient portal as the foundation platform may hinder effective scale-up. Any ePROM system would ideally require full integration with each site electronic healthcare record, and also a unified interface, to maximise the likelihood of success and utility. In a recent renal stakeholder summit aimed at developing a UK ePROM roadmap – involving patients, HCPs, academics and funders/renal organisations (including the Renal Association, British Renal Society, Kidney Care UK, National Kidney Federation, Kidney Research UK) – the development of a single online ePROM gateway/dashboard was identified as a key priority.[36] Such a dashboard would provide patients with a simple and consistent point of entry and allow them the flexibility to configure the platform to their liking, for example, around how reminders were configured/delivered, how their data and clinical advice were presented, or which primary/secondary care providers would have permissions to access their symptom information. Back-end development of APIs (application programming interfaces) would then allow permitted providers to securely 'pull' appropriate data into their electronic medical record, regardless of their underlying system architecture.

Strengths and limitations

This is the first UK study conducted in a CKD population that has explored the feasibility of ePROM capture/feedback with real-time integration within the electronic medical record. Our findings will help guide the design of a future RCT aimed at exploring efficacy and cost effectiveness. As this was a pilot study, no inferences can be made about the intervention's therapeutic efficacy. Nevertheless, clinical data around eGFR, risk of progression to kidney failure and health care utilisation show trends towards improvement in the intervention arm, suggesting further research is warranted.

The attrition rate for this study was larger than expected, owing to a higher proportion of patients progressing to kidney failure than anticipated (38% of patients randomised, versus 20% predicted). Whilst this demonstrated the effectiveness of our recruitment strategy, which targeted patients with advanced CKD at risk of progression, the sample size for a future trial may need to be adjusted accordingly to account for this observation depending on the exact nature of the primary outcome.

For peer review only - http://bmjopen.bmj.com/site/about/guidelines.xhtml

Finally, a sizable proportion of patients who were approached during study recruitment declined participation owing to concerns around internet access/computer inexperience. Whilst, anecdotally, reports suggest that patients have become much more comfortable with the use of digital healthcare necessitated during the COVID-19 pandemic, any future RCT should focus on broadening study accessibility and reducing the possibility of digital exclusion by: (i) ensuring the use of a simple user-friendly platform, with adequate training/support in place at the outset; and (ii) potentially providing an offline, e.g., paper-based, PRO option.

CONCLUSIONS

This single-centre, open-label, randomised controlled pilot study has demonstrated that it is feasible to conduct a trial incorporating online ePROM symptom reporting, with high rates of data completion. Based on patient/HCP feedback and system data, improvements to our ePROM intervention should be implemented to enhance functionality, long-term engagement and scalability prior to a multi-centre RCT.

For peer review only

DECLARATIONS

Consent for publication
Not applicable.

Availability of data and materials
The datasets used and/or analysed during the current study are available from the Birmingham Clinical Trials Unit upon reasonable request via the corresponding author.

Competing interests
EB, MCh, NI and JB report grants from NIHR. MCa is a NIHR Senior Investigator and receives funding from the NIHR Birmingham Biomedical Research Centre, the NIHR Surgical Reconstruction and Microbiology Research Centre and NIHR Applied Research Collaboration West Midlands at the University of Birmingham and University Hospitals Birmingham NHS Foundation Trust, Health Data Research UK, Innovate UK (part of UK Research and Innovation), Macmillan Cancer Support, UCB Pharma and GSK. MC has received personal fees from Astellas, Takeda, Merck, Daiichi Sankyo, Glaukos, GSK and the Patient-Centered Outcomes Research Institute (PCORI) outside the submitted work. DK reports grants from Macmillan Cancer Support, Innovate UK, the NIHR, NIHR Birmingham Biomedical Research Centre, and NIHR SRMRC at the University of Birmingham and University Hospitals Birmingham NHS Foundation Trust, and personal fees from Merck outside the submitted work.

Funding
This paper presents independent research funded by the National Institute for Health Research (NIHR) Post-Doctoral Fellowship Scheme, grant number PDF-2016-09-009.

Disclaimer
The views expressed are those of the author(s) and not necessarily those of the NHS, the NIHR or the Department of Health. The study sponsor and funders had no role in the study design, including collection, management, analysis and interpretation of data; writing of the report and the decision to submit the report for publication.

Author Contributions
DK is the chief investigator and takes final responsibility for study design, conduct and decision to submit for publication. DK prepared the first draft of the manuscript with approval from all authors. All investigators provided advice and critical input on the study design. All authors critically revised the manuscript for its important intellectual content. All authors read and approved the final manuscript.

Acknowledgements
The authors would like to thank all participants in the study. We would also like to thank the kidney research team and kidney care team at Queen Elizabeth Hospital Birmingham and the Birmingham Clinical Trials Unit for helping to run and deliver the trial. We would like to acknowledge Anita Walker for her administrative support and the RePROM Patient Advisory Group for their input into the design of the study. We thank all members of the Trial Steering Committee (Dr Andrew Mooney, Adult Renal Services, Lincoln Wing, St James University Hospital, Leeds, UK; Dr Kirstie Haywood, Warwick Research in Nursing, Warwick Medical School, University of Warwick, UK; Dr Mark Jesky, Department of Nephrology, Nottingham

For peer review only

University Hospitals NHS Trust, Nottingham, UK) for their advice and support. We would also like to thank Profs Ethan Basch (University of North Carolina, United States), Niels Hjöllund (Arhuus University, Denmark) and Galina Velikova (Patient Outcomes Group, University of Leeds, United Kingdom) for their support and design input.

Abbreviations

Albumin Creatinine Ratio: ACR
Blood pressure: BP
Chronic kidney disease: CKD
Electronic patient-reported outcome: ePROM
Estimated Glomerular Filtration Rate: eGFR
EuroQol five-level five-dimension PROM: EQ5D-5L
Glycated haemoglobin: HbA1c
Health Care Professionals: HCPs
Health-Related Quality of life: HRQoL
Inter Quartile Range: IQR
Queen Elizabeth Hospital Birmingham: QEHB
National Health Service: NHS
Randomised controlled trial: RCT

For peer review only

REFERENCES

1. Lockwood MB, Chung S, Puzantian H, et al. Symptom cluster science in chronic kidney disease: A literature review. *Western journal of nursing research* 2019;41(7):1056-91.
2. Almutary H, Bonner A, Douglas C. Symptom burden in chronic kidney disease: a review of recent literature. *J Ren Care* 2013;39(3):140-50. doi: 10.1111/j.1755-6686.2013.12022.x [published Online First: 2013/07/06]
3. Cabrera VJ, Hansson J, Kliger AS, et al. Symptom management of the patient with CKD: the role of dialysis. *Clinical Journal of the American Society of Nephrology* 2017;12(4):687-93.
4. Hassan R, Akbari A, Brown PA, et al. Risk factors for unplanned dialysis initiation: a systematic review of the literature. *Canadian journal of kidney health and disease* 2019;6:2054358119831684.
5. Arulkumaran N, Navaratnarajah A, Pillay C, et al. Causes and risk factors for acute dialysis initiation among patients with end-stage kidney disease-a large retrospective observational cohort study. *Clin Kidney J* 2019;12(4):550-58. doi: 10.1093/ckj/sfy118 [published Online First: 2019/08/07]
6. Mendelssohn DC, Curtis B, Yeates K, et al. Suboptimal initiation of dialysis with and without early referral to a nephrologist. *Nephrology Dialysis Transplantation* 2011;26(9):2959-65.
7. Calvert M, Kyte D, Price G, et al. Maximising the impact of patient reported outcome assessment for patients and society. *BMJ* 2019;364:k5267. doi: 10.1136/bmj.k5267 [published Online First: 2019/01/27]
8. Holch P, Warrington L, Bamforth L, et al. Development of an integrated electronic platform for patient self-report and management of adverse events during cancer treatment. *Annals of Oncology* 2017;28(9):2305-11.
9. Velikova G, Absolom K, Warrington L, et al. Phase III randomised controlled trial of eRAPID (electronic patient self-Reporting of Adverse-events: Patient Information and advice)—An eHealth intervention during chemotherapy. *Journal of Clinical Oncology* 2020;38(15_suppl):7002-02. doi: 10.1200/JCO.2020.38.15_suppl.7002
10. Basch E, Deal A, Kris M, et al. Symptom Monitoring With Patient-Reported Outcomes During Routine Cancer Treatment: A Randomised Controlled Trial. *Journal of Clinical Oncology* 2015;10.1200/JCO.2015.63.0830
11. Denis F, Lethrosne C, Pourel N, et al. Randomised trial comparing a web-mediated follow-up with routine surveillance in lung cancer patients. *JNCI: Journal of the National Cancer Institute* 2017;109(9)
12. Velikova G, Brown JM, Smith AB, et al. Computer-based quality of life questionnaires may contribute to doctor-patient interactions in oncology. *Br J Cancer* 2002;86(1):51-9. doi: 10.1038/sj.bjc.6600001 [published Online First: 2002/02/22]
13. Detmar SB, Muller MJ, Schornagel JH, et al. Health-related quality-of-life assessments and patient-physician communication: a randomised controlled trial. *JAMA* 2002;288(23):3027-34.
14. McCann L, Maguire R, Miller M, et al. Patients' perceptions and experiences of using a mobile phone-based advanced symptom management system (ASyMS©) to monitor and manage chemotherapy related toxicity. *European journal of cancer care* 2009;18(2):156-64.
15. Velikova G, Booth L, Smith AB, et al. Measuring quality of life in routine oncology practice improves communication and patient well-being: a randomised controlled trial. *Journal of Clinical Oncology* 2004;22(4):714-24.
16. Basch E, Deal AM, Dueck AC, et al. Overall survival results of a trial assessing patient-reported outcomes for symptom monitoring during routine cancer treatment. *Jama* 2017;318(2):197-98.
17. Denis F, Basch E, Septans A-L, et al. Two-year survival comparing web-based symptom monitoring vs routine surveillance following treatment for lung cancer. *Jama* 2019;321(3):306-07.
18. Eldridge SM, Chan CL, Campbell MJ, et al. CONSORT 2010 statement: extension to randomised pilot and feasibility trials. *bmj* 2016;355:i5239.
19. Kyte D, Bishop J, Brettell E, et al. Use of an electronic patient-reported outcome measure in the management of patients with advanced chronic kidney disease: the RePROM pilot trial

For peer review only - http://bmjopen.bmj.com/site/about/guidelines.xhtml

protocol. *BMJ Open* 2018;8(10):e026080. doi: 10.1136/bmjopen-2018-024617 [published Online First: 2019/02/21]
20. Kyte D, Anderson N, Auti R, et al. Development of an electronic patient-reported outcome measure (ePROM) system to aid the management of patients with advanced chronic kidney disease. *Journal of patient-reported outcomes* 2020;4(1):1-9.
21. Tangri N, Stevens LA, Griffith J, et al. A predictive model for progression of chronic kidney disease to kidney failure. *Jama* 2011;305(15):1553-59.
22. Kellum JA, Lameire N, Aspelin P, et al. Kidney disease: improving global outcomes (KDIGO) acute kidney injury work group. KDIGO clinical practice guideline for acute kidney injury. *Kidney international supplements* 2012;2(1):1-138.
23. Devlin NJ, Krabbe PF. The development of new research methods for the valuation of EQ-5D-5L. *The European Journal of Health Economics* 2013;14(1):1-3.
24. Lancaster GA, Dodd S, Williamson PR. Design and analysis of pilot studies: recommendations for good practice. *Journal of evaluation in clinical practice* 2004;10(2):307-12.
25. Browne RH. On the use of a pilot sample for sample size determination. *Statistics in medicine* 1995;14(17):1933-40.
26. Hsieh HF, Shannon SE. Three approaches to qualitative content analysis. *Qualitative health research* 2005;15(9):1277-88. doi: 10.1177/1049732305276687 [published Online First: 2005/10/06]
27. Walters SJ, dos Anjos Henriques-Cadby IB, Bortolami O, et al. Recruitment and retention of participants in randomised controlled trials: a review of trials funded and published by the United Kingdom Health Technology Assessment Programme. *BMJ open* 2017;7(3):e015276.
28. Absolom K, Warrington L, Hudson E, et al. Phase III Randomised Controlled Trial of eRAPID: eHealth Intervention During Chemotherapy. *Journal of Clinical Oncology* 2021:JCO. 20.02015.
29. Schick-Makaroff K, Molzahn AE. Evaluation of real-time use of electronic patient-reported outcome data by nurses with patients in home dialysis clinics. *BMC health services research* 2017;17(1):439.
30. Pittman ZC, John SG, McIntyre CW. Collection of daily patient reported outcomes is feasible and demonstrates differential patient experience in chronic kidney disease. *Hemodialysis International* 2017;21(2):265-73.
31. Morton R, Jose M, Brown C, et al. The symptom monitoring with feedback trial (swift): a novel registry-based cluster randomised controlled trial among australian and new zealand adults with end-stage kidney disease managed on haemodialysis. *Nephrology Dialysis Transplantation* 2019;34(Supplement_1):gfz096. FO31.
32. Symptom Monitoring With Feedback Trial (SWIFT): and ANZDATA registry-based cluster randomised trial. Electronic Patient-Reported Outcomes (ePROs) for the kidney patient community; 2020. UK Renal Assciation.
33. Walters SJ, Brazier JE. Comparison of the minimally important difference for two health state utility measures: EQ-5D and SF-6D. *Qual Life Res* 2005;14(6):1523-32. doi: 10.1007/s11136-004-7713-0 [published Online First: 2005/08/23]
34. Baigent C, Landray MJ, Reith C, et al. The effects of lowering LDL cholesterol with simvastatin plus ezetimibe in patients with chronic kidney disease (Study of Heart and Renal Protection): a randomised placebo-controlled trial. *Lancet* 2011;377(9784):2181-92. doi: 10.1016/s0140-6736(11)60739-3 [published Online First: 2011/06/15]
35. Bhandari S, Ives N, Brettell EA, et al. Multicentre randomised controlled trial of angiotensin-converting enzyme inhibitor/angiotensin receptor blocker withdrawal in advanced renal disease: the STOP-ACEi trial. *Nephrol Dial Transplant* 2016;31(2):255-61. doi: 10.1093/ndt/gfv346 [published Online First: 2015/10/03]
36. Electronic Patient-Reported Outcomes (ePROs) for the kidney patient community. Electronic Patient-Reported Outcomes (ePROs) for the kidney patient community Avaiable at: https://wwwyoutubecom/watch?v=JgG61Vouctk&feature=youtube and https://wwwyoutubecom/watch?v=iQ0mOVee_dg&feature=youtube[Accesed Jan 2021]; 2020. UK Renal Assciation.

For peer review only

FIGURE LEGENDS

Figure 1. Flow of participants through the trial

For peer review only

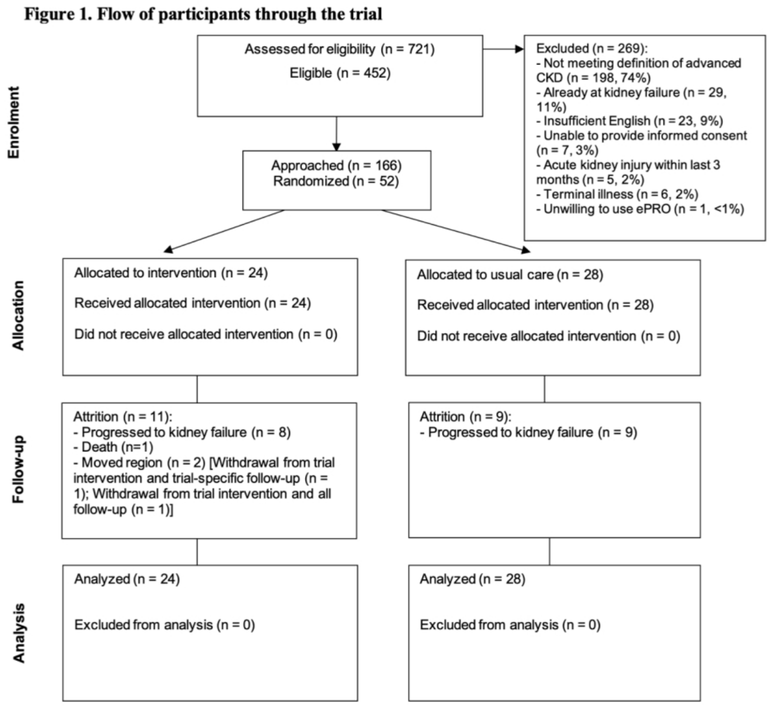

Figure 1. Flow of participants through the trial

594x419mm (72 x 72 DPI)

SUPPLEMENTARY APPENDIX

TITLE

Use of an electronic patient-reported outcome measure in the management of patients with advanced chronic kidney disease: the RePROM pilot trial

AUTHORSHIP

Derek Kyte[1,2] PhD, Nicola Anderson[2,4] MSc, Jon Bishop[5] PhD, Andrew Bissell[6], Elizabeth Brettell[5] BSc, Melanie Calvert[2,3] PhD, Marie Chadburn[5] PhD, Paul Cockwell[4] PhD, Mary Dutton[4] RN, Helen Eddington[4] MB ChB, Elliot Forster[4] BSc, Gabby Hadley[4] MSc, Natalie J Ives[2,5] MSc, Louise Jackson[7] PhD, Sonia O'Brien[6], Gary Price[2,6], Keeley Sharpe[6], Stephanie Stringer[4] MB ChB, Rav Verdi[6], Judi Waters[6], Adrian Wilcockson[5].

AUTHOR AFFILIATIONS

[1]School of Applied Health & Community, University of Worcester, Worcester, UK
[2] Centre for Patient-Reported Outcomes Research, Institute of Applied Health Research, University of Birmingham, Birmingham, UK
[3] NIHR Birmingham Biomedical Research Centre, University of Birmingham and University Hospitals Birmingham NHS Foundation Trust, UK
[4] University Hospitals Birmingham NHS Foundation Trust, Birmingham, UK
[5] Birmingham Clinical Trials Unit (BCTU), Institute of Applied Health Research, University of Birmingham, Birmingham, UK
[6] Patient Advisory Group, Centre for Patient-Reported Outcomes Research, Institute of Applied Health Research, University of Birmingham, Birmingham, UK
[7] Health Economics Unit, Institute of Applied Health Research, University of Birmingham, Birmingham, UK

CORRESPONDING AUTHOR

Dr Derek Kyte
Lecturer in Health Research Methods
NIHR Fellow
The Murray Learning Centre
University of Birmingham
Birmingham
B15 2TT

Telephone: 0121 415 8502
Email: d.g.kyte@bham.ac.uk

For peer review only

For peer review only - http://bmjopen.bmj.com/site/about/guidelines.xhtml

RePROM Participant Topic Guide v1.0 – 20/11/2017

Short project title: **RePROM**

Full project title: The use of an electronic Patient-Reported Outcome Measure in the Management of Patients with Advanced Chronic Kidney Disease – The RePROM Pilot Trial.

For peer review only

Participant Interview Topic Guide

Guidance notes to the interviewer

Note: *If the participant becomes distressed or unwell, the interviewer will adopt the following approaches, dependent upon the participant's wishes:*

1) If the participant wishes, the interviewer will suspend or terminate the interview, and will stay with the participant until they are feeling better.

2) If the participant has another person to provide care, at the request of the participant, the interviewer will either suspend the interview and leave the room, or will terminate the interview completely.

3) If the interviewer feels it is warranted, and if the participant agrees, he will put the participant in contact with an appropriate renal clinician.

4) If the interviewer feels that there is reason to be concerned for the physical/mental health of a participant, he will inform the participant of his intention to take the appropriate action, e.g. call the GP/Consultant.

Points to discuss with the participant prior to signing the consent form

- Recap on key information in the PIS
    - I will be recording this interview, so I have something to help me remember accurately what we talk about today, the only people who will hear the recording are myself and the person producing the transcript (who will sign a confidentiality aggrement), is this ok?
    - If there is anything you find you do not wish to talk about please let me know. I will aim to follow your lead in terms of what we discuss, but if we do stray on to a topic that you are not keen to talk about, tell me straight away and we can discuss something else.
    - We can stop the interview whenever you like. If you would like to take a break, or feel upset or unwell, please let me know and we will suspend or stop the interview entirely.

For peer review only - http://bmjopen.bmj.com/site/about/guidelines.xhtml

Verbal consent will be taken if participant still wishes to take part. **Note:** written consent for the interview will have been taken at the outset of the participant's involvement in the RePROM study.

Introduction to Interview

Thank you for agreeing to take part in this interview. The aim of this interview is to discuss your experience of being involved in the RePROM study. There are no 'right' or 'wrong' answers, we are interested in *your* views based on your experience. I am now going to start the recording.

Begin Interview

Main body of Interview

1) Can you explain how you first heard about the RePROM study?

2) Could you tell us what you felt was good about the recruitment process and whether any aspect could be improved?

3) What made you decide to take part in the RePROM study?

4) Can you explain what happened on your first study visit? What was good about this and what could be improved?

5) For the rest of your study visits, can you outline what was good and what could be improved?

For participants randomized to the ePROM reporting group:

6) Could you tell us about your first experience using the ePROM system?

Prompts
- Ease of myHealth sign-up and system log-in?
- Mode of administration, location, duration?
- Any problems? Ease of use?

7) Could you tell us about your subsequent experiences using the ePROM system?

Prompts
- Ease of myHealth sign-up and system log-in?
- Mode of administration, location, duration?
- Any problems? Ease of use?
- Alert experiences?

8) Could you tell us about whether/how the ePROM information you provided was discussed in your clinic appointments?

9) Could you tell us what was good about the ePROM system and what could be improved?

For peer review only - http://bmjopen.bmj.com/site/about/guidelines.xhtml

Post Interview – Debrief

- I have no more questions, but I'd like to give you the opportunity to say anything else about the RePROM study, your experience of completing the ePROM, or anything else we've discussed today?

- *Outline what will happen next:* (1) the recording will be typed up and annonymised, then analysed alongside all the other interviews, (2) we will send you a summary of this interview (unless you would prefer that we didn't) and will invite your comments. You do not have to comment on these results if you do not wish to.

- Finally, if you decide that you do not want what you have said today to be included in my research, you will need to tell me this within 5 working days – so by [*insert an actual day, according to timing of interview*]. After this it will be too late to withdraw as I will not be able to untangle what you have told me from what other people have told me.

- Thank you for taking part in the interview today.

For peer review only

For peer review only - http://bmjopen.bmj.com/site/about/guidelines.xhtml

RePROM Clinician and Staff Topic Guide v1.0 – 20/11/2017

RePROM Renal Electronic Patient Reported Outcome Measures

UNIVERSITY OF BIRMINGHAM

Short project title: **RePROM**

Full project title: The use of an electronic Patient-Reported Outcome Measure in the Management of Patients with Advanced Chronic Kidney Disease – The RePROM Pilot Trial.

Clinician/Staff Interview Topic Guide

Guidance notes to the interviewer

Note: *If the participant becomes distressed or unwell, the interviewer will adopt the following approaches, dependent upon the participant's wishes:*

1) If the participant wishes, the interviewer will suspend or terminate the interview, and will stay with the participant until they are feeling better.

2) If the participant has another person to provide care, at the request of the participant, the interviewer will either suspend the interview and leave the room, or will terminate the interview completely.

3) If the interviewer feels it is warranted, and if the participant agrees, he will put the participant in contact with an appropriate renal clinician.

4) If the interviewer feels that there is reason to be concerned for the physical/mental health of a participant, he will inform the participant of his intention to take the appropriate action, e.g. call the GP/Consultant.

Points to discuss with the participant prior to signing the consent form

- Recap on key information in the PIS
  - I will be recording this interview, so I have something to help me remember accurately what we talk about today, the only people who will hear the recording are myself and the person producing the transcript (who will sign a confidentiality aggrement), is this ok?
  - If there is anything you find you do not wish to talk about please let me know. I will aim to follow your lead in terms of what we discuss, but if we do stray on to a topic that you are not keen to talk about, tell me straight away and we can discuss something else.
  - We can stop the interview whenever you like. If you would like to take a break, or feel upset or unwell, please let me know and we will suspend or stop the interview entirely.

For peer review only - http://bmjopen.bmj.com/site/about/guidelines.xhtml

Written consent will be taken if participant still wishes to take part.

<u>Introduction to Interview</u>

Thank you for agreeing to take part in this interview. The aim of this interview is to discuss your experience of being involved in the RePROM study. There are no 'right' or 'wrong' answers, we are interested in *your* views based on your experience. I am now going to start the recording.

Begin Interview

Main body of Interview

1) Could you tell us what you felt was good about the recruitment process and whether any aspect could be improved?

Prompts
- Screening, eligibility check
- Approach, consent
- myHealth signup, ePROM training
- Baseline assessment

3) Could you tell us what you felt was good about the follow-up process and whether any aspect could be improved?

6) Could you tell us about your experience using the ePROM system?

Prompts
- Ease of use, usefulness of the data?
- Format of data presentation?
- Alert generation and management.
- What was good about the system and what could be improved?

7) Is there anything about the RePROM project design or implementation that we need to address/improve prior to conducting the planned RCT?

Post Interview – Debrief

- I have no more questions, but I'd like to give you the opportunity to say anything else about the RePROM study, your experience of using the ePROM system, or anything else we've discussed today?

- *Outline what will happen next:* (1) the recording will be typed up and annonymised, then analysed alongside all the other interviews, (2) we will send you a summary of this interview (unless you would prefer that we didn't) and will invite your comments. You do not have to comment on these results if you do not wish to.

For peer review only - http://bmjopen.bmj.com/site/about/guidelines.xhtml

- Finally, if you decide that you do not want what you have said today to be included in my research, you will need to tell me this within 5 working days – so by [*insert an actual day, according to timing of interview*]. After this it will be too late to withdraw as I will not be able to untangle what you have told me from what other people have told me.

- Thank you for taking part in the interview today.

For peer review only

For peer review only - http://bmjopen.bmj.com/site/about/guidelines.xhtml

Table S1. Case Report Form (CRF) returns.

Timepoint	CRF	Expected	Received (%)
Baseline	Consent	52	52 (100)
Baseline	CRF	52	52 (100)
Baseline	EQ5D-5L	52	52 (100)
3 Month	CRF	47	47 (100)
3 Month	EQ5D-5L	47	45 (96)
6 Month	CRF	41	41 (100)
6 Month	EQ5D-5L	41	41 (100)
9 Month	CRF	29	29 (100)
9 Month	EQ5D-5L	29	29 (100)
12 Month	CRF	18	18 (100)
12 Month	EQ5D-5L	18	18 (100)

EuroQol five-level five-dimension PROM, EQ5D-5L.

For peer review only

For peer review only - http://bmjopen.bmj.com/site/about/guidelines.xhtml

Table S2. ePROM intervention: overall symptom reporting, notifications and time taken to resolve.

Total number of participants randomised to ePROM intervention	Total number of symptoms reported	Total number of symptom notifications (%)	Total number of participants triggering notifications for severe and current symptoms (%)	Median time taken to resolve in minutes (IQR)
24	579	16 (3)	5 (25)	10 (6.5-22.5)

Electronic Patient-Reported Outcome, ePROM.

For peer review only

Table S3. ePROM intervention: notification pattern by symptom.

	Number of notifications triggered for severe + current symptoms (%)
Itchy/Dry skin	6 (37)
Fatigue	4 (25)
Shortness of breath	2 (13)
Pain	2 (13)
Difficulty sleeping	1 (6)
Ankle swelling	1 (6)
Lack of appetite	0 (0)
Nausea	0 (0)
Problems with fistula	0 (0)
Faintness/dizziness	0 (0)
Restless legs or difficulty keeping legs still	0 (0)
Diarrhoea	0 (0)

Electronic Patient-Reported Outcome, ePROM.

For peer review only - http://bmjopen.bmj.com/site/about/guidelines.xhtml

Table S4. ePROM intervention: staff response to notification.

Staff response to notification	Frequency (%)
Telephone counselling about symptom management	7 (78)
Brought clinic appointment forwards	2 (22)
Imaging/test orders	2 (22)
Medication initiation/change	1 (11)
Other	1 (11)
Referral to A&E	0 (0)
Referral to other NHS service	0 (0)

Electronic Patient-Reported Outcome, ePROM.

For peer review only

For peer review only - http://bmjopen.bmj.com/site/about/guidelines.xhtml

Table S5. Protocol deviations.

	Allocation	
Protocol deviation	Monthly ePROM reports (N = 24)	Usual care (N = 28)
Software error 19-Jun-2019 [resolved]	1	0
Informed Consent Form error	0	1

Electronic Patient-Reported Outcome, ePROM.

For peer review only

For peer review only - http://bmjopen.bmj.com/site/about/guidelines.xhtml

Table S6. Free text comments.

If you have had any other symptoms or problems that you would like the kidney team to be aware of please outline below:
A stomach upset overnight one evening. with indigestion. Resolved by taking a couple of Bisodol tablets
Anal fistulas
Ankle and lower leg swelling since [Date Redacted]. New symptom. Goes away overnight. No new shortness of breath.
Arthritis
Arthritis. psoriasis. diabetes. high blood pressure
Arthritis/psoriasis
been very pale and colleagues have commented on a "yellow" tinge
Blocked sinus's
Breathlessness increasing. Clinic [Date Redacted] - fluid at base of right lung
constipation
constipation. which is improving
Cough productive of clear mucus
Difficulty concentrating
Difficulty concentrating and feeling cold
Difficulty concentrating. Night swears.
Dry mouth. husky voice.
During last night's sleep. I woke up in the middle of the night [Date Redacted] and found that my pyjama top was soaked in sweat. Otherwise. felt OK?
During my last visit to the Renal team. Quinine Sulfate tablets were proscribed to assist with random over night leg cramps. Just to confirm that this medication has dramatically reduced the incidence of cramps. thank you.
Excessive mucus. no cold symptoms. but caused me to vomit and retch. Slight nosebleeds. Very poor appetite. UTI. Antibiotics prescribed by renal vascular team [Date Redacted] when doing first stage fistula. Ciprox
Feel a bit light headed this afternoon
Feeling cold
Feeling cold.
Felt very tired on [Dates Redacted] plus a stomach upset. probably as a result of the proceedure carried out [Date Redacted]?

For peer review only - http://bmjopen.bmj.com/site/about/guidelines.xhtml

For the last two nights I have had difficulty in sleeping after the first three hours or so. Additionally last night when I awoke in the middle of the night for a toilet break I had been sweating a very great deal. which is unusual for me.
Headaches. painful feet. like electric shocks
Increasing sleepiness. eg nodding off after meals
Inpatient [Dates redacted]
Joint swelling...pain in joints...headaches
Loss of taste
More sleepy' Prone to nod off
My bladder control is proving difficult. especially if I travel any distance. After two hours traveling. I often need to stop to empty my bladder and don't get much warning. This means I have to always be on the look out for a toilet where ever I go.
no
No
Not that I'm aware of.
No.
None
none
None
None at this time.
none known
None known
None.
Not that I am aware of
Not that I know of.
Pedal oedema - This was the presenting symptom to the team
Productive cough
Rash over upper body in small patches
Really bad cold
Severe and constant gout inflammatory knee joint

For peer review only - http://bmjopen.bmj.com/site/about/guidelines.xhtml

Severe headaches
Since [Date Redacted] I have had swollen ankles and legs. This goes away overnight. This is a new symptom. I have not been SOB.
Sleepiness previously reported has improved
Some nights I have been getting up three times to pass urine. However. I have just been given compression stockings by a Lymphoedema clinic to help with my swollen legs caused by taking Felodipine (mostly). This might help the problem...
Swelling in ankles due to hospitalisation. diarrhoea due to IV antibiotics for eye infection
Tending to drift off to sleep during the day more often
The Kidney team is aware and treatment is ongoing
Wheezy cough

For peer review only

For peer review only - http://bmjopen.bmj.com/site/about/guidelines.xhtml

CONSORT 2010 checklist of information to include when reporting a pilot or feasibility trial*

Section/Topic	Item No	Checklist item	Reported on page No
**Title and abstract**			
	1a	Identification as a pilot or feasibility randomised trial in the title	1
	1b	Structured summary of pilot trial design, methods, results, and conclusions (for specific guidance see CONSORT abstract extension for pilot trials)	2
**Introduction**			
Background and objectives	2a	Scientific background and explanation of rationale for future definitive trial, and reasons for randomised pilot trial	4
	2b	Specific objectives or research questions for pilot trial	4
**Methods**			
Trial design	3a	Description of pilot trial design (such as parallel, factorial) including allocation ratio	5-7
	3b	Important changes to methods after pilot trial commencement (such as eligibility criteria), with reasons	5
Participants	4a	Eligibility criteria for participants	5
	4b	Settings and locations where the data were collected	5
	4c	How participants were identified and consented	5
Interventions	5	The interventions for each group with sufficient details to allow replication, including how and when they were actually administered	5
Outcomes	6a	Completely defined prespecified assessments or measurements to address each pilot trial objective specified in 2b, including how and when they were assessed	6-7
	6b	Any changes to pilot trial assessments or measurements after the pilot trial commenced, with reasons	n/a
	6c	If applicable, prespecified criteria used to judge whether, or how, to proceed with future definitive trial	n/a
Sample size	7a	Rationale for numbers in the pilot trial	7
	7b	When applicable, explanation of any interim analyses and stopping guidelines	n/a
Randomisation:			
Sequence generation	8a	Method used to generate the random allocation sequence	6
	8b	Type of randomisation(s); details of any restriction (such as blocking and block size)	6
Allocation concealment mechanism	9	Mechanism used to implement the random allocation sequence (such as sequentially numbered containers), describing any steps taken to conceal the sequence until interventions were assigned	6

For peer review only

Implementation	10	Who generated the random allocation sequence, who enrolled participants, and who assigned participants to interventions	6
Blinding	11a	If done, who was blinded after assignment to interventions (for example, participants, care providers, those assessing outcomes) and how	6
	11b	If relevant, description of the similarity of interventions	n/a
Statistical methods	12	Methods used to address each pilot trial objective whether qualitative or quantitative	7
**Results**			
Participant flow (a diagram is strongly recommended)	13a	For each group, the numbers of participants who were approached and/or assessed for eligibility, randomly assigned, received intended treatment, and were assessed for each objective	8, Fig. 1
	13b	For each group, losses and exclusions after randomisation, together with reasons	8, Fig. 1
Recruitment	14a	Dates defining the periods of recruitment and follow-up	8
	14b	Why the pilot trial ended or was stopped	8
Baseline data	15	A table showing baseline demographic and clinical characteristics for each group	Table 1
Numbers analysed	16	For each objective, number of participants (denominator) included in each analysis. If relevant, these numbers should be by randomised group	Tables 2-6
Outcomes and estimation	17	For each objective, results including expressions of uncertainty (such as 95% confidence interval) for any estimates. If relevant, these results should be by randomised group	8-29, Tables 2-6
Ancillary analyses	18	Results of any other analyses performed that could be used to inform the future definitive trial	8--29
Harms	19	All important harms or unintended effects in each group (for specific guidance see CONSORT for harms)	8
	19a	If relevant, other important unintended consequences	n/a
**Discussion**			
Limitations	20	Pilot trial limitations, addressing sources of potential bias and remaining uncertainty about feasibility	31-32
Generalisability	21	Generalisability (applicability) of pilot trial methods and findings to future definitive trial and other studies	30-32
Interpretation	22	Interpretation consistent with pilot trial objectives and findings, balancing potential benefits and harms, and considering other relevant evidence	30-32
	22a	Implications for progression from pilot to future definitive trial, including any proposed amendments	30-32
**Other information**			
Registration	23	Registration number for pilot trial and name of trial registry	5
Protocol	24	Where the pilot trial protocol can be accessed, if available	5
Funding	25	Sources of funding and other support (such as supply of drugs), role of funders	33
	26	Ethical approval or approval by research review committee, confirmed with reference number	5

For peer review only

Citation: Eldridge SM, Chan CL, Campbell MJ, Bond CM, Hopewell S, Thabane L, et al. CONSORT 2010 statement: extension to randomised pilot and feasibility trials. BMJ. 2016;355.
*We strongly recommend reading this statement in conjunction with the CONSORT 2010, extension to randomised pilot and feasibility trials, Explanation and Elaboration for important clarifications on all the items. If relevant, we also recommend reading CONSORT extensions for cluster randomised trials, non-inferiority and equivalence trials, non-pharmacological treatments, herbal interventions, and pragmatic trials. Additional extensions are forthcoming: for those and for up to date references relevant to this checklist, see www.consort-statement.org.

For peer review only

BMJ Open

Results of a pilot feasibility randomised controlled trial exploring the use of an electronic patient-reported outcome measure in the management of UK patients with advanced chronic kidney disease.

Journal:	*BMJ Open*
Manuscript ID	bmjopen-2021-050610.R1
Article Type:	Original research
Date Submitted by the Author:	15-Nov-2021

Complete List of Authors:	Kyte, Derek; University of Worcester,<br>Anderson, Nicola; University Hospitals Birmingham NHS Foundation Trust<br>Bishop, Jon; Birmingham Clinical Trials Unit (BCTU), Institute of Applied Health Research, University of Birmingham, Birmingham, UK, Medical Statistician<br>Bissell, Andrew; University of Birmingham, Patient Advisory Group, Centre for Patient-Reported Outcomes Research, Institute of Applied Health Research<br>Brettell, Elizabeth; 1. Birmingham Clinical Trials Unit (BCTU), Institute of Applied Health Research University of Birmingham, Birmingham, UK, Birmingham Clinical Trials Unit<br>Calvert , Melanie ; University of Birmingham, Centre for Patient Reported Outcomes Research and Institute of Applied Health Research<br>Chadburn, Marie; University of Birmingham, Birmingham Clinical Trials Unit, Institute of Applied Health Research<br>Cockwell, Paul; Queen Elizabeth Hospital Birmingham, Department of Renal Medicine; University of Birmingham, Division of Infection and Immunity<br>Dutton, Mary; University Hospitals Birmingham NHS Foundation Trust, Renal Medicine<br>Eddington, Helen; University Hospitals Birmingham NHS Foundation Trust, Renal Medicine<br>Forster, Elliot; University Hospitals Birmingham NHS Foundation Trust<br>Hadley, Gabby; University Hospitals Birmingham NHS Foundation Trust, Renal Medicine<br>Ives, Natalie; University of Birmingham, BCTU<br>Jackson, Louise; University of Birmingham, Health Economics Unit<br>O'Brien, Sonia; Patient Advisory Group, Centre for Patient-Reported Outcomes Research, Institute of Applied Health Research, University of Birmingham, Birmingham, UK<br>Price, Gary; University of Birmingham, Patient Advisory Group Member, Centre for Patient-Reported Outcomes Research (CPROR)<br>Sharpe, Keeley; University of Birmingham, Patient Advisory Group Member, Centre for Patient-Reported Outcomes Research (CPROR)<br>Stringer, Stephanie; University Hospitals Birmingham NHS Foundation Trust, Renal Medicine<br>Verdi, Rav; University of Birmingham, Patient Advisory Group Member, Centre for Patient-Reported Outcomes Research (CPROR)

	Waters, Judi; University of Birmingham, Patient Advisory Group Member, Centre for Patient-Reported Outcomes Research (CPROR)<br>Wilcockson, Adrian; University of Birmingham
**Primary Subject Heading**:	Renal medicine
Secondary Subject Heading:	Patient-centred medicine
Keywords:	NEPHROLOGY, End stage renal failure < NEPHROLOGY, Clinical trials < THERAPEUTICS

I, the Submitting Author has the right to grant and does grant on behalf of all authors of the Work (as defined in the below author licence), an exclusive licence and/or a non-exclusive licence for contributions from authors who are: i) UK Crown employees; ii) where BMJ has agreed a CC-BY licence shall apply, and/or iii) in accordance with the terms applicable for US Federal Government officers or employees acting as part of their official duties; on a worldwide, perpetual, irrevocable, royalty-free basis to BMJ Publishing Group Ltd ("BMJ") its licensees and where the relevant Journal is co-owned by BMJ to the co-owners of the Journal, to publish the Work in this journal and any other BMJ products and to exploit all rights, as set out in our licence.

The Submitting Author accepts and understands that any supply made under these terms is made by BMJ to the Submitting Author unless you are acting as an employee on behalf of your employer or a postgraduate student of an affiliated institution which is paying any applicable article publishing charge ("APC") for Open Access articles. Where the Submitting Author wishes to make the Work available on an Open Access basis (and intends to pay the relevant APC), the terms of reuse of such Open Access shall be governed by a Creative Commons licence – details of these licences and which Creative Commons licence will apply to this Work are set out in our licence referred to above.

Other than as permitted in any relevant BMJ Author's Self Archiving Policies, I confirm this Work has not been accepted for publication elsewhere, is not being considered for publication elsewhere and does not duplicate material already published. I confirm all authors consent to publication of this Work and authorise the granting of this licence.

For peer review only

TITLE

Results of a pilot feasibility randomised controlled trial exploring the use of an electronic patient-reported outcome measure in the management of UK patients with advanced chronic kidney disease.

AUTHORSHIP

Derek Kyte[1,2] PhD, Nicola Anderson[2,3] MSc, Jon Bishop[4] PhD, Andrew Bissell[5], Elizabeth Brettell[4] BSc, Melanie Calvert[2,6-9,] PhD, Marie Chadburn[4] PhD, Paul Cockwell[3] PhD, Mary Dutton[3] RN, Helen Eddington[3] MB ChB, Elliot Forster[3] BSc, Gabby Hadley[3] MSc, Natalie J Ives[2,4] MSc, Louise Jackson[10] PhD, Sonia O'Brien[5], Gary Price[2,5], Keeley Sharpe[5], Stephanie Stringer[3] MB ChB, Rav Verdi[5], Judi Waters[5], Adrian Wilcockson[4].

AUTHOR AFFILIATIONS

[1] School of Applied Health & Community, University of Worcester, Worcester, UK

[2] Centre for Patient Reported Outcomes Research, Institute of Applied Health Research, College of Medical and Dental Sciences, University of Birmingham, UK.

[3] University Hospitals Birmingham NHS Foundation Trust, Birmingham, UK

[4] Birmingham Clinical Trials Unit (BCTU), Institute of Applied Health Research, University of Birmingham, Birmingham, UK

[5] Patient Advisory Group, Centre for Patient-Reported Outcomes Research, Institute of Applied Health Research, University of Birmingham, Birmingham, UK

[6] Birmingham Health Partners Centre for Regulatory Science and Innovation, University of Birmingham, Birmingham, UK

[7] National Institute for Health Research (NIHR) Birmingham Biomedical Research Centre, University of Birmingham, Birmingham, UK.

[8] National Institute for Health Research (NIHR) Applied Research Collaboration (ARC) West Midlands, University of Birmingham, Birmingham, UK

[9] National Institute for Health Research (NIHR) Surgical Reconstruction and Microbiology Research Centre University Hospitals Birmingham NHS Foundation Trust and University of Birmingham, UK

[10] Health Economics Unit, Institute of Applied Health Research, University of Birmingham, Birmingham, UK

CORRESPONDING AUTHOR

Dr Derek Kyte
Senior Lecturer
School of Allied Health and Community,
University of Worcester,
St John's Campus Henwick Grove,
Worcester,
WR2 6AJ

Email: d.kyte@worc.ac.uk

KEYWORDS

Chronic Kidney Disease, patient-reported outcomes, symptom monitoring, pilot trial, randomised controlled trial

WORDCOUNT

3887

For peer review only

ABSTRACT

Objectives
The use of routine remote follow-up of patients with chronic kidney disease (CKD) is increasing exponentially. It has been suggested that online electronic patient-reported outcome measures (ePROMs) could be used in parallel, to facilitate real-time symptom monitoring aimed at improving outcomes. We tested the feasibility of this approach in a pilot trial of ePROM symptom monitoring versus usual care in patients with advanced CKD not on dialysis.

Design
A 12-month, parallel, pilot randomised controlled trial (RCT) and qualitative sub-study.

Setting & Participants
Queen Elizabeth Hospital Birmingham, UK. Adult patients with advanced CKD (eGFR ≥6 and ≤15 mL/min/1.73m^2, or a projected risk of progression to kidney failure within 2-years ≥20%).

Intervention
Monthly online ePROM symptom reporting, including automated feedback of tailored self-management advice and triggered clinical notifications in the advent of severe symptoms. Real-time ePROM data were made available to the clinical team via the electronic medical record.

Outcomes
Feasibility (recruitment and retention rates, and acceptability/adherence to the ePROM intervention). Health-related quality of life, clinical data (e.g., measures of kidney function, kidney failure, hospitalisation, death) and healthcare utilisation.

Results
52 patients were randomised (31% of approached). Case report form returns were high (99.5%), as was retention (96%). Overall, 73% of expected ePROM questionnaires were received. Intervention adherence was high beyond 90 days (74%) and 180 days (65%); but dropped beyond 270 days (46%). Qualitative interviews supported proof of concept and intervention acceptability, but highlighted necessary changes aimed at enhancing overall functionality/scalability of the ePROM system.

Limitations
Small sample size.

Conclusions
This pilot trial demonstrates that patients are willing to be randomised to a trial assessing ePROM symptom monitoring. The intervention was considered acceptable; though measures to improve longer-term engagement are needed. A full-scale RCT is considered feasible.

Trial Registration
ISRCTN12669006

For peer review only

STRENGTHS AND LIMITATIONS OF THIS STUDY

- This is the first study to examine the feasibility of a clinical trial of ePROM use in a UK CKD population.
- Development of the study design was overseen by a patient advisory group, which included people with lived experience of CKD.
- The ePROM intervention was configured to allow real-time integration of participant's symptom data within the electronic medical record.
- As this was a pilot study, no inferences can be made about the intervention's therapeutic efficacy.
- Our findings will help guide the design of a future randomised controlled trial aimed at exploring efficacy and cost effectiveness.

For peer review only

BACKGROUND

Patients with advanced chronic kidney disease (CKD) commonly have a high symptom burden; increasingly so as they progress towards kidney failure.[1 2] Uncontrolled symptomology can be a particular source of anxiety and can have a detrimental impact on patient's health-related quality of life and outcomes.[1-3]

Timely detection of symptomatic deterioration is a key component of effective disease management during this period.[3] It can be challenging, however, to identify an unexpected decline in kidney function between scheduled clinic appointments, unless a patient self-refers. Unfortunately, some patients self-refer too late because they have difficulty identifying the point at which they may require assistance. Without prompt recognition of advanced symptoms, such patients are at high risk of severe illness, emergency hospitalisation, progression to unplanned kidney replacement therapy and significantly poorer long-term outcomes, including increased mortality.[4-6]

Routine systematic capture of symptom data using electronic patient-reported outcome (ePROM) measures has been suggested as a low-cost method of supporting symptom monitoring and control.[7] ePROM platforms provide patients with access to short online questionnaires that allow them to share self-reported symptom data with their clinical team, often in real time, to help guide care.[8] Systems may be configured to provide patients with tailored self-management advice and to trigger clinical notifications in the advent of sudden deterioration and/or severe symptomology.[9-11]

In studies involving patients with cancer, ePROM symptom monitoring is associated with enhanced patient-clinician communication; improved patient education and self-efficacy; better symptom control; earlier detection of adverse events; improved patient quality of life; reduced use of accident and emergency services; fewer inpatient hospital episodes; and improved survival; even for 'computer-inexperienced' patients.[9-17]

The efficacy of ePROM symptom monitoring for patients with advanced CKD, has not been investigated within a randomised controlled trial (RCT); nor has the feasibility of undertaking such a trial been established. This single-center pilot study aimed to assess the feasibility of undertaking a RCT investigating the use of monthly ePROM reporting compared with usual care in patients with advanced CKD not on dialysis.

For peer review only

METHODS

Reporting
This study is reported in accordance with the Consolidated Standards of Reporting Trials (CONSORT) checklist for reporting a pilot/feasibility trial.[18]

Study Design
RePROM (Renal electronic patient-reported outcome measure) was a single-centre, open-label, two-arm randomised controlled pilot/feasibility trial and qualitative sub-study. The trial was registered with ISRCTN (ISRCTN12669006) and the UK NIHR Portfolio (CPMS ID: 36497); and the protocol has been published.[19]

Study changes
Owing to changes in clinical practice at the host research site, made in response to the COVID-19 pandemic, the study received approval from the Health Research Authority for early closure of follow up (02/04/2020). This meant that follow up was truncated for some participants and that recruitment of health care professionals (HCPs) to the qualitative sub-study had to be suspended.

Study setting
The trial was undertaken within the Birmingham Clinical Trials Unit (BCTU) and Centre for Patient-Reported Outcomes Research at the University of Birmingham and the Queen Elizabeth Hospital Birmingham (QEHB) within the UK National Health Service (NHS) University Hospitals Birmingham Foundation Trust.

Patient and public involvement
Development of the study design was informed by a series of meetings held with our Patient Advisory Group (AB, SO, GP, KS, RV, JW), established in 2016, which included people with lived experience of CKD. Members were also involved in the ePROM intervention co-design group[20] and trial management group.

Study oversight
An independent steering committee was convened to provide guidance to the trial management group and to review feasibility data during the trial.

Study population
Eligible participants were adult (≥18 years old) patients under the care of the kidney services at QEHB, who met the trial definition of advanced CKD (estimated Glomerular Filtration Rate (eGFR) ≥6 and ≤15 mL/min/1.73m^2, or a projected risk of progression to kidney failure within 2-years ≥20% using the 4-variable Tangri renal risk equation[21]). Participants were excluded if they met any of the following criteria: patients unwilling to use the ePROM intervention; patients who, in the opinion of the consenting professional, could not speak, read or write English sufficiently well to complete the ePROM unaided; an episode of acute kidney injury (defined in accordance with international guidelines[22]) within the last 3 months; patients meeting the trial definition of kidney failure (receiving dialysis, or scheduled to start, in the next 2 weeks, had received (or had a scheduled date to receive) a kidney transplant; or an eGFR ≤5ml/min/1.73m^2); patients with a terminal illness that, in the opinion of the clinician assessing eligibility, was likely to lead to the death of the patient within 6 months of starting participation in the study.

Recruitment and randomisation

For peer review only - http://bmjopen.bmj.com/site/about/guidelines.xhtml

Members of the kidney research team at QEHB screened for potentially eligible study participants using the inclusion/exclusion criteria. Those considered eligible were provided with a patient information sheet and given the opportunity to consider participation. For patients wishing to take part in the pilot trial (and optional qualitative sub-study), consent, enrolment and baseline data collection was conducted face-to-face in clinic. Randomisation was provided via a web-based system developed by BCTU. Participants were randomised at the level of the individual in a 1:1 ratio to usual care (control arm) or usual care supplemented with monthly online symptom reporting using the ePROM system (experimental arm). Minimisation was used to achieve balance between: 2-year risk of progression to kidney failure (<40%, versus ≥40%, based on the 4-variable Tangri renal risk equation[21]); self-reported computer experience (regular use of a computer, tablet or smartphone at least weekly, versus less than weekly); and patient-reported ethnicity ('white' versus 'non-white').

Intervention

Participants allocated to the ePROM intervention arm were asked to complete and submit monthly symptom questionnaires using an online system and received an automated reminder to do so. In addition, patients were allowed to submit any number of additional 'ad-hoc' questionnaires at any time outside of the scheduled monthly reporting dates. Development and functionality of the ePROM system has been described in detail elsewhere.[20] In summary, upon questionnaire submission, automated self-management advice was provided to patients based on their responses; questionnaire data was integrated into the QEHB electronic medical record and made available to HCPs in real-time; and a system algorithm triggered an automated notification which was sent to both the patient and the clinical team in the event of a severe and current symptom report. Participants allocated to the control arm received usual care. It was not possible to blind clinicians or participants due to the nature of the intervention.

Outcomes

As this was a pilot trial there was no single primary outcome measure. The primary aims of the study were to pilot the trial protocol and assess the feasibility of undertaking a full-scale RCT exploring the use of ePROMs in the management of advanced CKD. The feasibility outcomes included the following: the proportion of eligible participants approached to take part in the trial; the proportion of eligible participants who took part in the trial; recruitment rate: the proportion of participants randomised / screened; the proportion of participants randomised / approached; the proportion of participants who completed the trial (retention); and the proportion of participants who adhered to the ePROM intervention.

This pilot trial was not powered to detect differences in outcome measures, but provided an opportunity to ensure that there were no issues with completion of the outcome data and proposed outcome measures for the main RCT. The following outcome data were collected:

- Health-related quality of life, using the paper version of the EuroQol five-dimension, five level, questionnaire (EQ-5D-5L). The EQ-5D-5L is a reliable/validated generic measure of health status/utility commonly used internationally in cost-effectiveness and ePROM research.[10 23]
- Clinical data, including serum creatinine, calcium, phosphate, bicarbonate, albumin, eGFR, albumin-to-creatinine ratio (ACR), blood pressure, and, for participants with diabetes: glucose and glycated haemoglobin (HbA1c).
- Event data: progression to kidney failure, contacts with healthcare professionals in secondary care (outpatient clinics and accident and emergency), inpatient hospitalisation, death.
- Additional healthcare resource use data was also collected at each study visit.

For peer review only - http://bmjopen.bmj.com/site/about/guidelines.xhtml

All data were collected at baseline and 3, 6, 9 and 12 months (assessment window ±3 weeks).

Sample size

As this was a pilot trial, no formal sample size calculation was performed. Following recommendations for pilot studies, 30 patients or more are typically required to obtain estimates of the parameters needed for sample size estimation.[24 25] To allow for a 10% drop-out and loss to follow-up, this pilot trial aimed to recruit at least 33 participants in each group, a total of 66 participants. This would allow the recruitment and retention rates to be estimated with 95% confidence interval maximum widths of 20% and 25% respectively.

Statistical analysis

Analysis of feasibility and clinical outcomes was based on all participants screened and recruited. For each binary outcome, the number and percentage are reported along with an exact binomial 95% confidence interval. Estimates of differences between groups are presented as relative risks obtained from log-binomial regression models. These estimates were unadjusted due to the low number of observed events. For continuous outcomes, the means and 95% confidence intervals are reported. Estimates of differences between groups are presented as differences in means adjusted for minimisation variables and, for longitudinal outcomes, the corresponding baseline values. All estimates of differences are presented with 95% confidence intervals. No p-values are reported as no hypothesis testing was performed. Analysis was conducted using SPSS software, v26 (IBM) and SAS software, version 9.4 (SAS Institute). Participants were analysed in the intervention group to which they were randomised, and all participants were included whether or not they received the allocated intervention (intention-to-treat). The study dataset and statistical analysis plan are available on request.

Qualitative sub-study

The qualitative sub-study aimed to explore patient and HCP thoughts/experiences regarding the RePROM trial processes and intervention. Semi-structured interviews were conducted by the lead author according to pre-defined topic guides (Supplementary Appendix), but there was sufficient scope to explore novel themes where appropriate. All interviews were digitally recorded, professionally transcribed and the transcripts anonymised. Transcript data were entered into a specialist software package (Dedoose, v8.3.35) to aid organisation and analysis of the data. All data were analysed by the lead author using conventional content analysis.[26] Interview transcripts were examined in depth by DK, prior to first cycle coding, in which content was coded around positive and negative perceptions regarding the intervention, as well as suggested system changes.

For peer review only

RESULTS

Patients and follow up

Recruitment was conducted at QEHB over 12 months from October 2019. The last follow-up was conducted in April 2020, which was truncated for 14 participants due to the COVID-19 pandemic. In total, 721 patients were screened, of which 452 (63%, 95%CI 59-66) were eligible, and 166 were approached to take part in the trial (37% of eligible, 95%CI 32-41). Fifty-two patients were randomised (Figure 1) (consent rate (of approached) = 31%, 95%CI 24-39; consent rate (of eligible) = 12%, 95%CI 9-15), representing 79% of the recruitment target sample size (recruitment rate (of approached) = 31%, 95%CI 24-39; recruitment rate (of screened) = 7%, 95%CI 5-9; average monthly recruitment rate = 4.3). The minimisation algorithm provided appropriate balance over 2-year risk of progression to kidney failure, however an error in the algorithm led to an imbalance in patient-reported ethnicity between groups. All participants self-reported as regular computer users.

Average follow up was 8.0 months (SD 3.8). In total, n=2 patients withdrew from the trial during follow up after moving geographical region (both withdrew from the intervention and 1 from all follow-up) (retention = 96%, 95%CI 87-100). During the study, n=17 patients met the trial definition of kidney failure (the study protocol mandated exit at this point) and there was n=1 death. No patients were excluded from the analysis. Case report form return rates were excellent throughout (99.5% of all expected forms received) (Supplementary Appendix, Table S1).

[Figure 1 near here]

The main reason for non-approach of screened and eligible individuals was that patients had not registered to use the existing hospital patient portal 'MyHealth' (90% of those not approached). For patients that were approached, but who were not willing to take part, reported reasons included: 'no internet access/computer inexperienced' (45%); 'not interested in research' (22%); 'too burdensome (completing ePROMs)' (11%); 'too burdensome (general)' (11%); 'issues with myHealth patient portal sign-up' (9%); 'unwell/health-related reasons' (2%); 'too burdensome (travel/trial visits)' (2%).

The average age of participants was 57 years (range 25-86), 29% were female, 37% reported 'non-white' ethnicity, 96% reported secondary level education or greater and 100% reported regular use of a computer, tablet or smartphone at least weekly. Mean baseline eGFR was 15.2, the average 2-year Tangri risk of progression to kidney failure was 43%, and the average EQ-5D index was 0.74 (Table 1).

For peer review only - http://bmjopen.bmj.com/site/about/guidelines.xhtml

Table 1. Baseline characteristics.

		Monthly ePROM reports (N = 24)	Usual care (N = 28)	Overall (N = 52)
**Minimisation variables**				
Risk progression	<40%	11 (46%)	14 (50%)	25 (48%)
	≥40%	13 (54%)	14 (50%)	27 (52%)
Self-reported computer experience*	'Yes'	24 (100%)	28 (100%)	52 (100%)
	'No'	0 (0%)	0 (0%)	0 (0%)
Ethnicity	'white'	18 (75%)	15 (54%)	33 (63%)
	'non-white'	6 (25%)	13 (46%)	19 (37%)
**Demographic and other baseline variables**				
Age, years	Mean (95% CI)	58 (51-65)	56 (50-61)	57 (52-61)
Gender	Female	7 (29%)	8 (29%)	15 (29%)
	Male	17 (71%)	20 (71%)	37 (71%)
Highest level of education	Higher education (e.g., Bachelors/ Masters/Professional degree/ PhD)	9 (38%)	9 (32%)	18 (35%)
	Further education (e.g., A-Levels / Vocational training)	9 (38%)	7 (25%)	16 (31%)
	Secondary education (e.g., GCSEs/O-levels)	6 (25%)	10 (36%)	16 (31%)
	Primary education	0 (0%)	0 (0%)	0 (0%)
	No qualifications	0 (0%)	2 (7%)	2 (4%)
	Not known	0 (0%)	0 (0%)	0 (0%)
Baseline medical history	Hypertension	17 (71%)	25 (89%)	42 (81%)
	Atrial Fibrillation	1 (4%)	1 (4%)	2 (4%)
	Ischaemic Heart Disease	2 (8%)	4 (14%)	6 (12%)
	Peripheral Vascular Disease	0 (0%)	3 (11%)	3 (6%)

For peer review only

	Diabetes (Type I)	2 (8%)	4 (14%)	6 (12%)
	Diabetes (Type II)	7 (29%)	8 (29%)	15 (29%)
	Cerebrovascular Disease	0 (0%)	0 (0%)	0 (0%)
	Chronic Respiratory Disorder	2 (8%)	2 (7%)	4 (8%)
	Thyroid Disease	0 (0%)	0 (0%)	0 (0%)
	Rheumatoid Arthritis	0 (0%)	1 (4%)	1 (2%)
	Anxiety/Depression	0 (0%)	2 (7%)	2 (4%)
	Cancer	6 (25%)	1 (4%)	7 (13%)
Systolic BP (mmHg)	Mean (95% CI)	147.6 (139.1-156.0)	146.0 (139.9-152.1)	146.8 (141.7-151.8)
Diastolic BP (mmHg)	Mean (95% CI)	78.8 (75.2-82.4)	77.4 (72.9-81.8)	78.0 (75.2-80.9)
Health-Related Quality of Life (EQ-5D-5L index)	Mean (95% CI)	0.70 (0.60-0.80)	0.78 (0.71-0.85)	0.74 (0.68-0.80)
2-year Tangri[1] risk of progression to kidney failure	Mean (95% CI)	0.48 (0.40-0.57)	0.43 (0.34-0.51)	0.45 (0.39-0.51)
eGFR (mL/min/1,73 m^2)	Mean (95% CI)	14.0 (12.5-15.6)	15.7 (13.9-17.5)	14.9 (13.7-16.1)
Creatinine (µmol/L)	Mean (95% CI)	384.0 (345.8-422.2)	357.5 (316.3-398.8)	369.8 (341.4-398.1)
Calcium (µmol/L)	Mean (95% CI)	2.2 (2.2-2.3)	2.3 (2.2-2.3)	2.3 (2.2-2.3)
Bicarbonate (µmol/L)	Mean (95% CI)	20.8 (19.8-21.9)	21.3 (20.3-22.2)	21.1 (20.4-21.7)
Phosphate (µmol/L)	Mean (95% CI)	1.4 (1.3-1.5)	1.4 (1.3-1.5)	1.4 (1.3-1.5)
Albumin (g/L)	Mean (95% CI)	40.4 (38.2-42.6)	40.8 (39.0-42.7)	40.6 (39.2-42.0)
ACR (mg/mmol)	Median (IQR)	206.1 (126.9-285.2)	178.1 (109.7-246.4)	191.0 (139.5-242.5)
Blood Glucose (mmol/L)#	Mean (95% CI)	8.4 (6.8-9.9)	7.0 (5.6-8.4)	7.6 (6.5-8.6)
	Missing	1 (2%)	1 (2%)	2 (4%)
HbA1c (mmol/mol)#	Mean (95% CI)	57.2 (42.8-71.6)	53.2 (44.0-62.5)	54.6 (47.1-62.2)
	Missing	4 (8%)	3 (6%)	7 (14%)

*defined as regular use of a computer, tablet or smartphone at least weekly; #for diabetic participants. [1] Tangri N, Stevens LA, Griffith J, et al. A predictive model for progression of chronic kidney disease to kidney failure. Jama. 2011;305(15):1553-1559.[21] Electronic patient-reported Outcome, ePROM; Inter Quartile Range, IQR; blood pressure, BP; EuroQol five-level five-dimension PRO, EQ5D-5L; estimated Glomerular Filtration Rate, eGFR; Albumin Creatinine Ratio, ACR; glycated haemoglobin, HbA1c.

For peer review only - http://bmjopen.bmj.com/site/about/guidelines.xhtml

ePROM Intervention adherence and reporting patterns

Overall, 73% (95%CI 67-79) of expected ePROM questionnaires were received during the trial (Table 2). However, only 31% (95%CI 25-37) were received within our *a priori* agreed compliance window (72-hours either side of the scheduled reminder date). Patients submitted 98 'ad-hoc' questionnaires outside of this compliance window: an average of 4 per participant. Compliance over time was good, with a high proportion of participants submitting at least one scheduled questionnaire beyond 90 days post-randomisation (74%, 95%CI 52-90) and after 180 days (65%, 95%CI 41-85) but this proportion dropped beyond 270 days (46%, 95%CI 19-75).

For peer review only

Table 2. ePROM compliance.

Total number of expected ePROM questionnaires*	Total received (%, 95%CI))	Total number submitted in compliance window** (%, 95%CI)	Total number of ad-hoc ePROM questionnaire submissions	Mean number of ad-hoc submissions per patient	Number of patients on trial >90 days	Proportion of patients submitting ePROM questionnaires >90 days (95%CI)	Number of patients on trial >180 days	Proportion of patients submitting ePROM questionnaires >180 days (95%CI)	Number of patients on trial >270 days	Proportion of patients submitting ePROM questionnaires >270 days (95%CI)
230	169 (73, 67-79)	71 (31, 25-37)	98	4	23	74% (52-90)	20	65% (41-85)	13	46% (19-75)

*accounting for questionnaire allocation date and loss to follow-up/withdrawals/death/progression to kidney failure; **questionnaires received within a +/- 72-hour time window. electronic patient-reported Outcome, ePROM.

For peer review only

Patients reported 579 symptoms, the most prevalent of which included fatigue, shortness of breath, itchy/dry skin and pain (Table 3, n=20 patients reported symptoms during the trial, n=4 did not report any symptoms). Most symptoms reported were mild (60%). There were 16 severe and current symptom reports (across 13 questionnaires), generated by 5 patients, representing 3% of the total number of symptoms reported across the trial (for full details around system notifications see Supplementary Appendix, Tables S2, S3 and S4). The symptoms driving these notifications were itchy/dry skin (37% of notifications), fatigue (25%), shortness of breath (13%), pain (13%), difficulty sleeping (6%) and ankle swelling (6%). The median time taken by staff to resolve patient notifications was 10 minutes (IQR 6.5-22.5) and actions included: ‘telephone counselling about symptom management’ (78%); and ‘brought clinic appointment forwards’ (22%); ‘imaging/test orders’ (22%); ‘medication initiation/change’ (11%); ‘other’ (11%), more than one type of action could be recorded for each notification (see Supplementary Appendix Table S4).

For peer review only

Table 3. ePROM intervention: reporting pattern by symptom.

		Number of symptoms reported			
	Number of times reported	Mild (%)	Moderate (%)	Severe (%)	Proportion of total symptoms reported (N = 579)
Fatigue	135	69 (51)	60 (44)	6 (4)	23%
Shortness of breath	109	88 (81)	17 (16)	4 (4)	19%
Itchy/dry skin	102	53 (52)	42 (41)	7 (7)	18%
Pain	87	54 (62)	29 (33)	4 (5)	15%
Lack of appetite	57	35 (61)	22 (39)	0 (0)	10%
Ankle swelling	21	11 (52)	9 (43)	1 (5)	4%
Nausea	20	13 (65)	7 (35)	0 (0)	3%
Difficulty sleeping	17	7 (41)	9 (53)	1 (6)	3%
Faintness/dizziness	11	6 (55)	5 (45)	0 (0)	2%
Restless legs or difficulty keeping legs still	10	7 (70)	3 (30)	0 (0)	2%
Diarrhoea	10	5 (50)	5 (50)	0 (0)	2%
Problems with fistula	0	0 (0)	0 (0)	0 (0)	0%
TOTALS	579	348 (60)	208 (36)	23 (4)	

Electronic patient-reported outcome, ePROM.

For peer review only

Clinical outcomes, patient-reported outcomes and healthcare utilisation

Clinical and patient-reported outcome data are available in the supplementary appendix (Tables S5 and S6). As expected, there were high levels of uncertainty around all point estimates given the limited size of the sample.

Healthcare utilisation data appears in Table 4. In summary, patients in the intervention arm reported 97 fewer episodes of healthcare utilisation than those in the usual care arm (mean number of episodes per patient: intervention arm = 10.3, usual care arm = 12.3; intervention arm 0.11 fewer mean episodes per month on trial), which included 54 fewer CKD-related specialist kidney clinic visits (mean per patient: intervention arm = 5.4, usual care arm = 6.5; intervention arm 0.07 fewer episodes per month on trial). Hospital inpatient stay was similar in both arms. Again, this exploratory data should be treated with caution owing to the small sample size.

For peer review only

Table 4. Summary of healthcare utilisation.

	CKD-Related				Not CKD-Related				CKD relationship unknown			
	Intervention (N = 24)		Usual care (N = 28)		Intervention (N = 24)		Usual care (N = 28)		Intervention (N = 24)		Usual care (N = 28)	
NHS service category	Episodes	NHS hospital inpatient stay (days)	Episodes	NHS hospital inpatient stay (days)	Episodes	NHS hospital inpatient stay (days)	Episodes	NHS hospital inpatient stay (days)	Episodes	NHS hospital inpatient stay (days)	Episodes	NHS hospital inpatient stay (days)
GP appointment	1		4 (n=2)		14 (n=9)		23 (n=15)		0		4 (n=2)	
GP out of hours service	0		0		0		1		0		0	
Specialist kidney clinic	129 (n=22)		183 (n=26)		1		0		0		0	
NHS outpatient clinic (other than specialist kidney clinic)	10 (n=6)		15 (n=12)		41 (n=13)		74 (n=17)		1		1	
NHS walk-in centre	0		0		1		0		0		0	
NHS 111/NHS direct telephone call	0		0		1		1		0		0	
A&E	1		0		2 (n=2)		5 (n=3)		1		1	
NHS hospital inpatient stay	4 (n=3)	7	2 (n=2)	2	2 (n=2)	7	2 (n=2)	8	0		2 (n=2)	2
Other:	9 (n=5)		19 (n=13)		27 (n=4)		8 (n=3)		2 (n=2)		0	
Imaging	3		6		2		1		1		0	
Home visit	2		5		0		0		0		0	
Phlebotomy	1		1		0		0		0		0	

For peer review only - http://bmjopen.bmj.com/site/about/guidelines.xhtml

Health education/roadshow/open day	1		1		0		0		0		0	
Chemotherapy	0		0		8		0		0		0	
Ophthalmology procedure	0		0		1		0		0		0	
Other (NHS)	2		5		1		7		1		0	
Other (private)	0		0		15		0		0		0	
TOTALS	154 (n=22)	7	222 (n=26)		89 (n=14)		114 (n=23)		4 (n=2)		8 (n=4)	

General practice, GP; National Health Service, NHS; Accident and Emergency, A&E.

For peer review only

Safety, protocol deviations

There was 1 serious adverse event (n=1 death) reported during the trial. Two protocol deviations were recorded, 1 software error (resolved) and 1 informed consent form error (missing initial) (Supplementary Appendix, Table S7).

For peer review only

Qualitative sub-study

Semi-structured interviews were conducted with 24 trial participants (intervention arm n=14; usual care arm n=10). Interviewee responses supported proof of concept and acceptability and indicated that the system had met our four-fold remit[20]:

1. To allow patients with advanced CKD to remotely self-report their symptoms using a simple and secure online platform.
2. To provide appropriate self-management advice to patients whose ePROM scores highlighted one or more mild/moderate/severe symptoms.
3. To allow monitoring of real-time patient ePROM symptom data and subsequent automated notification of both the patient and the clinical team in the advent of a severe symptom.
4. To incorporate longitudinal ePROM symptom data in the electronic patient record to help inform clinical consultations and support shared understanding/decision-making.

A summary of qualitative findings regarding intervention positives/negatives and suggested system changes is presented in Table 5. Patients highlighted benefits around login security; questionnaire structure, clarity and coverage; and felt reassurance that their questionnaire data, including their free text comments (Supplementary Appendix Table S8), were being monitored and responded to promptly and/or discussed in clinic. They also reported that the advice around symptoms and self-management was useful and helped alleviate anxiety around the symptoms they were experiencing.

The main system shortfalls, identified across the whole sample, included: failures of the reminder process meaning some patients did not receive reminder emails; a lack of clarity for some patients around which questionnaire they should complete at which timepoint and confusion around how to view self-management advice; difficulty navigating/scrolling through sections; occasional problems for some patients when submitting the questionnaire. Interviewees suggested a range of changes aimed at addressing these shortfalls and enhancing the overall functionality of the system.

We experienced HCP recruitment challenges owing to healthcare pressures secondary to the COVID-19 pandemic. This meant that only 1 HCP interview was completed, precluding robust thematic analysis. We present the summary data in the supplementary appendix (Table S9) for completeness.

For peer review only

Table 5. Summary of qualitative findings regarding intervention positives/negatives and suggested system changes.

**Theme** *Subtheme*	Illustrative quote(s)
**Intervention positives**	
Questionnaire data acted upon	"On a few occasions I was very impressed that what I had put on the form, obviously had been noticed and had been picked up. And was discussed with me at clinic and I thought that was one of the big positives of the form itself." [Patient 01]
Provided reassurance	"…it does give you some reassurance if you can be told, well that's normal for the problems you've got." [Patient 02]
Quick to complete	"The first one probably took me quarter of an hour because I read through it very carefully and double checked what I was saying as I went along. But once I'd done a couple then it was sort of less than ten minutes… I sort of answered the questions as I felt at the time… But it was a breeze once I got used to it that was fine it was easy to fill in." [Patient 03]
Alleviated anxiety	"I found it positive. I think it takes worries away to be honest with you… You have the advice that was given, so you didn't feel as if you're the only person that ever-had itchiness before. It was obviously something that was very common. So, I would have said it alleviated any anxiety, for me." [Patient 01]
Questionnaire structure/content	"I think the questions, they're quite clear and quite precise." [Patient 04]; "…my symptoms… headaches, itchy skin, swelling which it covered, tiredness which it covered… I think it covered everything from my point of view." [Patient 05]
Provision of guidance	"…it prompted you to give the QE a ring and discuss it, you know what I mean… you know like feeling worse and feeling tired or whatever, just to ring up and speak to somebody cause sometimes you don't… you just don't do that… you just carry on, you just carry on till your next appointment. So, it made you think about it." [Patient 06]
Immediate clinical assistance	"…it's nice to know that, you know… if anything is going wrong then I can get help more or less straightaway." [Patient 07]
Free-text comments	"Initially I was filling the form in and putting very little additional information on. Latterly I was putting a lot more information on and I was very pleased on two occasions that when I went for my renal check-up, the points that I'd made had been noticed and were brought up… it was an additional form of communication in that if I'd got a concern or something was happening, I could put it on the form… and you could use it to answer questions then as to how you were coping, what you were doing and how you were feeling." [Patient 01]
Self-management advice	"…very useful because as a lay person not understanding the functions of the body, not that well if you see what I mean, it's useful sometimes to get a bit of guidance as to where you need to go." [Patient 03]
Login security	"…I think the security of, if you like, the double tier I think is very, very good indeed." [Patient 08]
User-friendly	"I think it's quite simple and user friendly." [Patient 04]
**Intervention negatives**	
Reminder failures	"…some of the time it didn't come through on my daughter's iPhone and then it would come through the next month but miss a month… Seemed to be hit and miss sometimes." [Patient 07]
Questionnaire completion	"The complicated bit, which I did struggle with, was trying to get up the latest questionnaire, which needed to be completed…" [Patient 08]; "I would actually number the questionnaires so you can tell which ones you've done and completed… sometimes I didn't know which ones I'd done and which ones I hadn't done…" [Patient 05]
Prominence of next steps and self-management advice	"Yeah, I don't remember seeing too much of that [information] at the end of it to be honest." [Patient 15]

For peer review only - http://bmjopen.bmj.com/site/about/guidelines.xhtml

Difficulty navigating through multiple sections within the system	"…for some reason one of the sections within a section… I could scroll down but the inner bar I couldn't scroll down completely… there were like 10 questions, maybe 12 questions, and you could get down to question eight, but I couldn't get down to the last two…" [Patient 09]
Difficulty submitting the questionnaire	"…on two separate occasions we did try and fill it out but then the problem is there was never a finish or a continuation of the questionnaire, so we couldn't exactly finish it..." [Patient 10]
**Suggested system changes**	
Improve reminders	"…perhaps like my daughter found that, you know, it was hit and miss when the questionnaire [reminders] came through. That could be improved on…" [Patient 07]
Enhance/simplify interface	"…navigating your way through the electronic system… could be made a bit easier." [Patient 08]
Incorporate dietary advice	"…my major one really, which I've been surprised at, was the lack of information regarding, you know, diet…" [Patient 11]
Incorporate questions around psychological wellbeing/mood	"I think just having that questionnaire to see how your mood is and how you can look back on it and see where, like, how you can improve and how you can change it slightly and try and move on from there…" [Patient 10]
Timing of questionnaire completion related to clinical encounter/receiving results	"I'm getting the [clinic] results sometimes before I answer the questionnaire, and I think that possibly can end in user bias 'cause if my results are not very good then sometimes that can translate into feeling bad, you know, rather than the other way round, if you know what I mean?" [Patient 12]
Incorporate other symptom questions	"I think it's worthwhile [adding]… leg cramps… it's just when you're in bed at night and lying down. It'll be like absolutely agonising, just like really painful… it is one of the key symptoms, yeah." [Patient 04]
Tick-box option to prompt contact with the clinical team	"I'd perhaps have the tick box at the end of the questions… to say 'could somebody ring you' would be a good idea… for someone to give you that reassurance with a phone call… of how to ease the symptoms." [Patient 05]
Simplify the questionnaire submission process	"I found a little bit of confusion on the last page where you, they showed you your answers, what you'd put, there's submit button on that page. I had to come back a page to submit it, that caused confusion a couple of times." [Patient 01]
Make data available to GPs	"…the GP side of things in the UK isn't necessarily that well linked into the hospital system… with the technology that we have these days you'd think that it would be sensible to have the GP on if you like a version of 'MyHealth' so they can see exactly what the hospital are seeing, obviously within the rules of confidentiality… I think the more integrated it is the better it will work" [Patient 03]
Combine questionnaire data with other clinical/lifestyle information collected at home	"…it was just my wondering whether there was another level perhaps… whether blood pressure something like that…things like the blood pressure and weight I have to record every day anyway…" [Patient 13]
Consider flexibility in setting notification thresholds for different symptoms	"Have the same system as the failsafe system but don't have it as severe. Maybe say level three, make it to level two or level one." [Patient 14]

For peer review only

DISCUSSION

In this single centre open-label randomised study, we examined the feasibility of randomising patients with advanced CKD to monthly ePROM reporting with real-time feedback of data or to usual care. We found that the majority of study indicators supported the feasibility of a full-scale RCT: patient eligibility rate (proportion of screened patients eligible) 63%; recruitment rate (of patients approached) 31%; case report form returns 99.5%; and retention 96%. In total, 52 patients were randomised (monthly recruitment rate = 4.3), representing 79% of the recruitment target sample size (N = 66). This level of recruitment would position the study in the top quartile of performance based on a review of recruitment and retention across 151 RCTs funded by the UK Health Technology Assessment Programme.[27] Moreover, overall adherence to the intervention was good, with patients returning 73% of expected ePROM questionnaires, although not always in the specified time windows. We have therefore demonstrated that it is possible to randomise and follow up patients with high levels of data completion through to 12 months, and that a RCT is feasible.

Within our study, we found the observed pattern of ePROM reporting did not correspond with our *a priori* expectations. Relatively few patients submitted their questionnaires within our pre-specified compliance window (72-hours either side of the scheduled submission date). Triangulation with qualitative data suggested that it was unlikely that this observation was related to issues around acceptability of the intervention: all participants indicated positive engagement with the system. Moreover, overall questionnaire return rates were high. A number of patients reported a failure to receive email reminders, or that emails were sent to junk folders, which may have contributed to out-of-window submissions: where patients relied on memory, rather than external prompts. Several patients suggested adding a mobile text reminder option, which they felt would be more reliable. It was our initial intention to include such an option, unfortunately, this was not possible within the existing patient portal framework. This feature will be made available as a priority within the next iteration of the system.

Our overall findings around feasibility align with similar research conducted in oncology. The feasibility of trial-based exploration of ePROM efficacy in this area has been well established and a number of trials successfully completed internationally, in the US[10], France[11] and in the UK.[28] Within kidney research, whilst the feasibility of routine collection of ePROMs in clinical practice has been supported[29 30], there has been relatively little research around trial feasibility until recently. The 'symptom monitoring with feedback trial' (SWIFT), is a registry-based pilot cluster randomised controlled trial among Australian and New Zealand adults with end-stage kidney disease managed on haemodialysis; due for completion in 2020/21.[31] Early findings from the pilot study suggest feasibility and acceptability when implementing ePROMs with feedback to clinicians in Australian haemodialysis centres, supporting progress to a follow-on multicentre RCT.[32]

Previous ePROM trials have commonly included a primary outcome based around health-related quality of life, for example, measured using the EQ-5D.[10] Based on our study population data, it would require a total of 348 participants to detect a clinically meaningful 0.07 reduction in EQ-5D-5L index[33] (SD=0.18, p=0.05, 90% power, adjusting for 20% attrition). This sample size appears achievable based on the successful implementation of previous UK-led kidney trials with similar (or greater) sample size requirements.[34 35]

Whilst the study intervention was well received by patients and demonstrated proof of concept, there were a number of suggested improvements that may enhance longer-term engagement with the system, for example: simplification the interface and, in particular, improvements to the reminder functionality; incorporation of automated dietary advice; and the inclusion of additional questionnaire items around the psychological impacts associated with CKD. In addition, it was suggested that use of the intervention within a multi-centre trial may necessitate system-level

For peer review only - http://bmjopen.bmj.com/site/about/guidelines.xhtml

modifications to ensure compatibility with different IT infrastructures at other hospitals. Work conducted within a UK oncology setting has shown that it is possible to integrate a single ePROM system across multiple NHS trusts, each with unique IT platforms, but that repeated integration at each separate site often takes considerable time and resources.[9] Our own experience of linking an ePROM to an existing hospital-based patient portal was mixed. Positives included the in-built security aspects, which some patients particularly valued, and also the ability to share data within the electronic medical record relatively easily. Negatives included functionality issues around the interface and the lack of some important features, e.g., text reminders and smartphone compatibility. In addition, issues with sign-up to the patient portal for some patients meant that study staff could not approach them to take part in the trial without first arranging access to the patient portal, which created a substantial barrier to recruitment.

Looking ahead to the roll-out of an ePROM system within a multicentre trial, and also considering future potential implementation in clinical practice, the use of a single hospital patient portal as the foundation platform may hinder effective scale-up. Any ePROM system would ideally require full integration with the electronic healthcare record at each site, and also a unified interface, to maximise the likelihood of success and utility. In a recent renal stakeholder summit aimed at developing a UK ePROM roadmap – involving patients, HCPs, academics and funders/renal organisations (including the Renal Association, British Renal Society, Kidney Care UK, National Kidney Federation, Kidney Research UK) – the development of a single online ePROM gateway/dashboard was identified as a key priority.[36] Such a dashboard would provide patients with a simple and consistent point of entry and allow them the flexibility to configure the platform to their liking, for example, around how reminders were configured/delivered, how their data and clinical advice were presented, or which primary/secondary care providers would have permissions to access their symptom information. Back-end development of APIs (application programming interfaces) would then allow permitted healthcare providers to securely 'pull' appropriate data into their electronic medical record, regardless of their underlying system architecture.

Strengths and limitations

This is the first UK study conducted in a CKD population that has explored the feasibility of ePROM capture/feedback with real-time integration within the electronic medical record. Our findings will help guide the design of a future RCT aimed at exploring efficacy and cost effectiveness. As this was a pilot study, no inferences can be made about the intervention's therapeutic efficacy. Nevertheless, clinical data around eGFR, risk of progression to kidney failure and health care utilisation show trends towards improvement in the intervention arm, suggesting further research is warranted.

The attrition rate for this study was larger than expected, owing to a higher proportion of patients progressing to kidney failure than anticipated (38% of patients randomised, versus 20% predicted). Whilst this demonstrated the effectiveness of our recruitment strategy, which targeted patients with advanced CKD at risk of progression, the sample size for a future trial may need to be adjusted accordingly to account for this observation depending on the exact nature of the primary outcome.

During the qualitative process analysis, it was not possible to conduct dual-coding or triangulation, this should be taken into account when interpreting the findings.

The pre-specified data analysis plan for this pilot study did not stipulate capture of the reason for starting dialysis, only the start date and type of dialysis was recorded.

Finally, a sizable proportion of patients who were approached during study recruitment declined participation owing to concerns around internet access/computer inexperience. Whilst, anecdotally, reports suggest that patients have become much more comfortable with the use of digital

For peer review only - http://bmjopen.bmj.com/site/about/guidelines.xhtml

healthcare necessitated during the COVID-19 pandemic, any future RCT should focus on broadening study accessibility and reducing the possibility of digital exclusion by: (i) ensuring the use of a simple user-friendly platform, with adequate training/support in place at the outset; and (ii) potentially providing an offline, e.g., paper-based, PRO option.

CONCLUSIONS

This UK single-centre, open-label, randomised controlled pilot study has demonstrated that it is feasible to conduct a trial incorporating online ePROM symptom reporting, with high rates of data completion. Based on patient feedback and system data, improvements to our ePROM intervention should be implemented to enhance functionality, long-term engagement and scalability prior to a multi-centre RCT.

For peer review only

DECLARATIONS

Consent for publication
Not applicable.

Availability of data and materials
The datasets used and/or analysed during the current study are available from the Birmingham Clinical Trials Unit upon reasonable request via the corresponding author.

Competing interests
EB, MCh, NI and JB report grants from NIHR. MCa is a NIHR Senior Investigator and receives funding from the NIHR Birmingham Biomedical Research Centre, the NIHR Surgical Reconstruction and Microbiology Research Centre and NIHR Applied Research Collaboration West Midlands at the University of Birmingham and University Hospitals Birmingham NHS Foundation Trust, Health Data Research UK, Innovate UK (part of UK Research and Innovation), Macmillan Cancer Support, UCB Pharma and GSK. MC has received personal fees from Astellas, Takeda, Merck, Daiichi Sankyo, Glaukos, GSK and the Patient-Centered Outcomes Research Institute (PCORI) outside the submitted work. DK reports grants from Macmillan Cancer Support, Innovate UK, the NIHR, NIHR Birmingham Biomedical Research Centre, and NIHR SRMRC at the University of Birmingham and University Hospitals Birmingham NHS Foundation Trust, and personal fees from Merck outside the submitted work.

Funding
This paper presents independent research funded by the National Institute for Health Research (NIHR) Post-Doctoral Fellowship Scheme, grant number PDF-2016-09-009.

Disclaimer
The views expressed are those of the author(s) and not necessarily those of the NHS, the NIHR or the Department of Health and social care. The study sponsor and funders had no role in the study design, including collection, management, analysis and interpretation of data; writing of the report and the decision to submit the report for publication.

Author Contributions
DK is the chief investigator and takes final responsibility for study design, conduct and decision to submit for publication. DK led the study design process with input from NA, JB, AB, EB, MCa, MCh, PC, MD, HE, GH, NI, LJ, SO, GP, KS, SS, RV and JW. DK, NA, EB, MCh, PC, MD, HE, EF, GH, SS and AW were involved in the acquisition of data. DK and JB conducted the analysis with support from NI. DK prepared the first draft of the manuscript with approval from all authors. All investigators (DK, NA, JB, AB, EB, MCa, MCh, PC, MD, HE, GH, NI, LJ, SO, GP, KS, SS, RV, JW, EF, AW) provided critical input regarding the interpretation of findings, were involved in revising the manuscript for its important intellectual content and read and approved the final manuscript.

Ethics Statement
This study was approved by the West Midlands Edgbaston Research Ethics Committee (Ref: 18/WM/0013) on 23rd February 2018 (ePROM finalisation and pilot trial).

Acknowledgements
The authors would like to thank all participants in the study. We would also like to thank the kidney research team and kidney care team at Queen Elizabeth Hospital Birmingham and the Birmingham Clinical Trials Unit for helping to run and deliver the trial. We would like to acknowledge Anita

For peer review only - http://bmjopen.bmj.com/site/about/guidelines.xhtml

Walker for her administrative support and the RePROM Patient Advisory Group for their input into the design of the study. We thank all members of the Trial Steering Committee (Dr Andrew Mooney, Adult Renal Services, Lincoln Wing, St James University Hospital, Leeds, UK; Dr Kirstie Haywood, Warwick Research in Nursing, Warwick Medical School, University of Warwick, UK; Dr Mark Jesky, Department of Nephrology, Nottingham University Hospitals NHS Trust, Nottingham, UK) for their advice and support. We would also like to thank Profs Ethan Basch (University of North Carolina, United States), Niels Hjöllund (Arhuus University, Denmark) and Galina Velikova (Patient Outcomes Group, University of Leeds, United Kingdom) for their support and design input.

Abbreviations

Albumin Creatinine Ratio: ACR
Blood pressure: BP
Chronic kidney disease: CKD
Electronic patient-reported outcome: ePROM
Estimated Glomerular Filtration Rate: eGFR
EuroQol five-level five-dimension PROM: EQ5D-5L
Glycated haemoglobin: HbA1c
Health Care Professionals: HCPs
Health-Related Quality of life: HRQoL
Inter Quartile Range: IQR
Queen Elizabeth Hospital Birmingham: QEHB
National Health Service: NHS
Randomised controlled trial: RCT

For peer review only

REFERENCES

1. Lockwood MB, Chung S, Puzantian H, et al. Symptom cluster science in chronic kidney disease: A literature review. *Western journal of nursing research* 2019;41(7):1056-91.
2. Almutary H, Bonner A, Douglas C. Symptom burden in chronic kidney disease: a review of recent literature. *J Ren Care* 2013;39(3):140-50. doi: 10.1111/j.1755-6686.2013.12022.x [published Online First: 2013/07/06]
3. Cabrera VJ, Hansson J, Kliger AS, et al. Symptom management of the patient with CKD: the role of dialysis. *Clinical Journal of the American Society of Nephrology* 2017;12(4):687-93.
4. Hassan R, Akbari A, Brown PA, et al. Risk factors for unplanned dialysis initiation: a systematic review of the literature. *Canadian journal of kidney health and disease* 2019;6:2054358119831684.
5. Arulkumaran N, Navaratnarajah A, Pillay C, et al. Causes and risk factors for acute dialysis initiation among patients with end-stage kidney disease-a large retrospective observational cohort study. *Clin Kidney J* 2019;12(4):550-58. doi: 10.1093/ckj/sfy118 [published Online First: 2019/08/07]
6. Mendelssohn DC, Curtis B, Yeates K, et al. Suboptimal initiation of dialysis with and without early referral to a nephrologist. *Nephrology Dialysis Transplantation* 2011;26(9):2959-65.
7. Calvert M, Kyte D, Price G, et al. Maximising the impact of patient reported outcome assessment for patients and society. *BMJ* 2019;364:k5267. doi: 10.1136/bmj.k5267 [published Online First: 2019/01/27]
8. Holch P, Warrington L, Bamforth L, et al. Development of an integrated electronic platform for patient self-report and management of adverse events during cancer treatment. *Annals of Oncology* 2017;28(9):2305-11.
9. Velikova G, Absolom K, Warrington L, et al. Phase III randomised controlled trial of eRAPID (electronic patient self-Reporting of Adverse-events: Patient Information and advice)—An eHealth intervention during chemotherapy. *Journal of Clinical Oncology* 2020;38(15_suppl):7002-02. doi: 10.1200/JCO.2020.38.15_suppl.7002
10. Basch E, Deal A, Kris M, et al. Symptom Monitoring With Patient-Reported Outcomes During Routine Cancer Treatment: A Randomised Controlled Trial. *Journal of Clinical Oncology* 2015;10.1200/JCO.2015.63.0830
11. Denis F, Lethrosne C, Pourel N, et al. Randomised trial comparing a web-mediated follow-up with routine surveillance in lung cancer patients. *JNCI: Journal of the National Cancer Institute* 2017;109(9)
12. Velikova G, Brown JM, Smith AB, et al. Computer-based quality of life questionnaires may contribute to doctor-patient interactions in oncology. *Br J Cancer* 2002;86(1):51-9. doi: 10.1038/sj.bjc.6600001 [published Online First: 2002/02/22]
13. Detmar SB, Muller MJ, Schornagel JH, et al. Health-related quality-of-life assessments and patient-physician communication: a randomised controlled trial. *JAMA* 2002;288(23):3027-34.
14. McCann L, Maguire R, Miller M, et al. Patients' perceptions and experiences of using a mobile phone-based advanced symptom management system (ASyMS©) to monitor and manage chemotherapy related toxicity. *European journal of cancer care* 2009;18(2):156-64.
15. Velikova G, Booth L, Smith AB, et al. Measuring quality of life in routine oncology practice improves communication and patient well-being: a randomised controlled trial. *Journal of Clinical Oncology* 2004;22(4):714-24.
16. Basch E, Deal AM, Dueck AC, et al. Overall survival results of a trial assessing patient-reported outcomes for symptom monitoring during routine cancer treatment. *Jama* 2017;318(2):197-98.
17. Denis F, Basch E, Septans A-L, et al. Two-year survival comparing web-based symptom monitoring vs routine surveillance following treatment for lung cancer. *Jama* 2019;321(3):306-07.

For peer review only - http://bmjopen.bmj.com/site/about/guidelines.xhtml

18. Eldridge SM, Chan CL, Campbell MJ, et al. CONSORT 2010 statement: extension to randomised pilot and feasibility trials. *bmj* 2016;355:i5239.
19. Kyte D, Bishop J, Brettell E, et al. Use of an electronic patient-reported outcome measure in the management of patients with advanced chronic kidney disease: the RePROM pilot trial protocol. *BMJ Open* 2018;8(10):e026080. doi: 10.1136/bmjopen-2018-024617 [published Online First: 2019/02/21]
20. Kyte D, Anderson N, Auti R, et al. Development of an electronic patient-reported outcome measure (ePROM) system to aid the management of patients with advanced chronic kidney disease. *Journal of patient-reported outcomes* 2020;4(1):1-9.
21. Tangri N, Stevens LA, Griffith J, et al. A predictive model for progression of chronic kidney disease to kidney failure. *Jama* 2011;305(15):1553-59.
22. Kellum JA, Lameire N, Aspelin P, et al. Kidney disease: improving global outcomes (KDIGO) acute kidney injury work group. KDIGO clinical practice guideline for acute kidney injury. *Kidney international supplements* 2012;2(1):1-138.
23. Devlin NJ, Krabbe PF. The development of new research methods for the valuation of EQ-5D-5L. *The European Journal of Health Economics* 2013;14(1):1-3.
24. Lancaster GA, Dodd S, Williamson PR. Design and analysis of pilot studies: recommendations for good practice. *Journal of evaluation in clinical practice* 2004;10(2):307-12.
25. Browne RH. On the use of a pilot sample for sample size determination. *Statistics in medicine* 1995;14(17):1933-40.
26. Hsieh HF, Shannon SE. Three approaches to qualitative content analysis. *Qualitative health research* 2005;15(9):1277-88. doi: 10.1177/1049732305276687 [published Online First: 2005/10/06]
27. Walters SJ, dos Anjos Henriques-Cadby IB, Bortolami O, et al. Recruitment and retention of participants in randomised controlled trials: a review of trials funded and published by the United Kingdom Health Technology Assessment Programme. *BMJ open* 2017;7(3):e015276.
28. Absolom K, Warrington L, Hudson E, et al. Phase III Randomised Controlled Trial of eRAPID: eHealth Intervention During Chemotherapy. *Journal of Clinical Oncology* 2021:JCO. 20.02015.
29. Schick-Makaroff K, Molzahn AE. Evaluation of real-time use of electronic patient-reported outcome data by nurses with patients in home dialysis clinics. *BMC health services research* 2017;17(1):439.
30. Pittman ZC, John SG, McIntyre CW. Collection of daily patient reported outcomes is feasible and demonstrates differential patient experience in chronic kidney disease. *Hemodialysis International* 2017;21(2):265-73.
31. Morton R, Jose M, Brown C, et al. The symptom monitoring with feedback trial (swift): a novel registry-based cluster randomised controlled trial among australian and new zealand adults with end-stage kidney disease managed on haemodialysis. *Nephrology Dialysis Transplantation* 2019;34(Supplement_1):gfz096. FO31.
32. Symptom Monitoring With Feedback Trial (SWIFT): and ANZDATA registry-based cluster randomised trial. Electronic Patient-Reported Outcomes (ePROs) for the kidney patient community; 2020. UK Renal Assciation.
33. Walters SJ, Brazier JE. Comparison of the minimally important difference for two health state utility measures: EQ-5D and SF-6D. *Qual Life Res* 2005;14(6):1523-32. doi: 10.1007/s11136-004-7713-0 [published Online First: 2005/08/23]
34. Baigent C, Landray MJ, Reith C, et al. The effects of lowering LDL cholesterol with simvastatin plus ezetimibe in patients with chronic kidney disease (Study of Heart and Renal Protection): a randomised placebo-controlled trial. *Lancet* 2011;377(9784):2181-92. doi: 10.1016/s0140-6736(11)60739-3 [published Online First: 2011/06/15]
35. Bhandari S, Ives N, Brettell EA, et al. Multicentre randomised controlled trial of angiotensin-converting enzyme inhibitor/angiotensin receptor blocker withdrawal in advanced renal

For peer review only

For peer review only - http://bmjopen.bmj.com/site/about/guidelines.xhtml

disease: the STOP-ACEi trial. *Nephrol Dial Transplant* 2016;31(2):255-61. doi: 10.1093/ndt/gfv346 [published Online First: 2015/10/03]

36. Electronic Patient-Reported Outcomes (ePROs) for the kidney patient community. Electronic Patient-Reported Outcomes (ePROs) for the kidney patient community Avaiable at: https://wwwyoutubecom/watch?v=JgG61Vouctk&feature=youtube and https://wwwyoutubecom/watch?v=iQ0mOVee_dg&feature=youtube[Accesed Jan 2021]; 2020. UK Renal Assciation.

For peer review only

FIGURE LEGENDS

Figure 1. Flow of participants through the trial

For peer review only

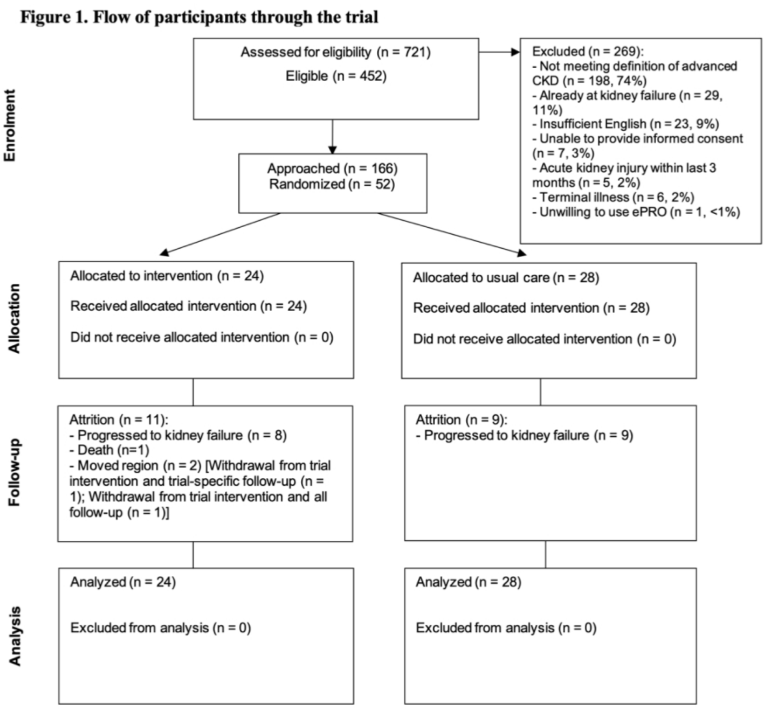

Figure 1, Flow of participants through the trial

142x100mm (300 x 300 DPI)

SUPPLEMENTARY APPENDIX

TITLE

Results of a pilot feasibility randomised controlled trial exploring the use of an electronic patient-reported outcome measure in the management of UK patients with advanced chronic kidney disease.

AUTHORSHIP

Derek Kyte[1,2] PhD, Nicola Anderson[2,3] MSc, Jon Bishop[4] PhD, Andrew Bissell[5], Elizabeth Brettell[4] BSc, Melanie Calvert[2,6-9,] PhD, Marie Chadburn[4] PhD, Paul Cockwell[3] PhD, Mary Dutton[3] RN, Helen Eddington[3] MB ChB, Elliot Forster[3] BSc, Gabby Hadley[3] MSc, Natalie J Ives[2,4] MSc, Louise Jackson[10] PhD, Sonia O'Brien[5], Gary Price[2,5], Keeley Sharpe[5], Stephanie Stringer[3] MB ChB, Rav Verdi[5], Judi Waters[5], Adrian Wilcockson[4].

AUTHOR AFFILIATIONS

[1] School of Applied Health & Community, University of Worcester, Worcester, UK
[2] Centre for Patient Reported Outcomes Research, Institute of Applied Health Research, College of Medical and Dental Sciences, University of Birmingham, UK.
[3] University Hospitals Birmingham NHS Foundation Trust, Birmingham, UK
[4] Birmingham Clinical Trials Unit (BCTU), Institute of Applied Health Research, University of Birmingham, Birmingham, UK
[5] Patient Advisory Group, Centre for Patient-Reported Outcomes Research, Institute of Applied Health Research, University of Birmingham, Birmingham, UK
[6] Birmingham Health Partners Centre for Regulatory Science and Innovation, University of Birmingham, Birmingham, UK
[7] National Institute for Health Research (NIHR) Birmingham Biomedical Research Centre, University of Birmingham, Birmingham, UK.
[8] National Institute for Health Research (NIHR) Applied Research Collaboration (ARC) West Midlands, University of Birmingham, Birmingham, UK
[9] National Institute for Health Research (NIHR) Surgical Reconstruction and Microbiology Research Centre University Hospitals Birmingham NHS Foundation Trust and University of Birmingham, UK
[10] Health Economics Unit, Institute of Applied Health Research, University of Birmingham, Birmingham, UK

CORRESPONDING AUTHOR

Dr Derek Kyte
Senior Lecturer
School of Allied Health and Community,
University of Worcester,
St John's Campus Henwick Grove,
Worcester,
WR2 6AJ

Email: d.kyte@worc.ac.uk

For peer review only

For peer review only - http://bmjopen.bmj.com/site/about/guidelines.xhtml

RePROM Participant Topic Guide v1.0 – 20/11/2017

UNIVERSITY OF BIRMINGHAM

Short project title: **RePROM**

Full project title: The use of an electronic Patient-Reported Outcome Measure in the Management of Patients with Advanced Chronic Kidney Disease – The RePROM Pilot Trial.

Participant Interview Topic Guide

Guidance notes to the interviewer

Note: *If the participant becomes distressed or unwell, the interviewer will adopt the following approaches, dependent upon the participant's wishes:*

1) If the participant wishes, the interviewer will suspend or terminate the interview, and will stay with the participant until they are feeling better.

2) If the participant has another person to provide care, at the request of the participant, the interviewer will either suspend the interview and leave the room, or will terminate the interview completely.

3) If the interviewer feels it is warranted, and if the participant agrees, he will put the participant in contact with an appropriate renal clinician.

4) If the interviewer feels that there is reason to be concerned for the physical/mental health of a participant, he will inform the participant of his intention to take the appropriate action, e.g. call the GP/Consultant.

Points to discuss with the participant prior to signing the consent form

- Recap on key information in the PIS
    - I will be recording this interview, so I have something to help me remember accurately what we talk about today, the only people who will hear the recording are myself and the person producing the transcript (who will sign a confidentiality aggrement), is this ok?
    - If there is anything you find you do not wish to talk about please let me know. I will aim to follow your lead in terms of what we discuss, but if we do stray on to a topic that you are not keen to talk about, tell me straight away and we can discuss something else.

For peer review only - http://bmjopen.bmj.com/site/about/guidelines.xhtml

- We can stop the interview whenever you like. If you would like to take a break, or feel upset or unwell, please let me know and we will suspend or stop the interview entirely.

Verbal consent will be taken if participant still wishes to take part. **Note:** written consent for the interview will have been taken at the outset of the participant's involvement in the RePROM study.

Introduction to Interview

Thank you for agreeing to take part in this interview. The aim of this interview is to discuss your experience of being involved in the RePROM study. There are no 'right' or 'wrong' answers, we are interested in *your* views based on your experience. I am now going to start the recording.

For peer review only

Begin Interview

Main body of Interview

1) Can you explain how you first heard about the RePROM study?

2) Could you tell us what you felt was good about the recruitment process and whether any aspect could be improved?

3) What made you decide to take part in the RePROM study?

4) Can you explain what happened on your first study visit? What was good about this and what could be improved?

5) For the rest of your study visits, can you outline what was good and what could be improved?

For participants randomized to the ePROM reporting group:

6) Could you tell us about your first experience using the ePROM system?

Prompts
- Ease of myHealth sign-up and system log-in?
- Mode of administration, location, duration?
- Any problems? Ease of use?

7) Could you tell us about your subsequent experiences using the ePROM system?

Prompts
- Ease of myHealth sign-up and system log-in?
- Mode of administration, location, duration?
- Any problems? Ease of use?

For peer review only - http://bmjopen.bmj.com/site/about/guidelines.xhtml

- Alert experiences?

8) Could you tell us about whether/how the ePROM information you provided was discussed in your clinic appointments?

9) Could you tell us what was good about the ePROM system and what could be improved?

Post Interview – Debrief

- I have no more questions, but I'd like to give you the opportunity to say anything else about the RePROM study, your experience of completing the ePROM, or anything else we've discussed today?

- *Outline what will happen next:* (1) the recording will be typed up and annonymised, then analysed alongside all the other interviews, (2) we will send you a summary of this interview (unless you would prefer that we didn't) and will invite your comments. You do not have to comment on these results if you do not wish to.

- Finally, if you decide that you do not want what you have said today to be included in my research, you will need to tell me this within 5 working days – so by [*insert an actual day, according to timing of interview*]. After this it will be too late to withdraw as I will not be able to untangle what you have told me from what other people have told me.

- Thank you for taking part in the interview today.

For peer review only

For peer review only - http://bmjopen.bmj.com/site/about/guidelines.xhtml

RePROM Clinician and Staff Topic Guide v1.0 – 20/11/2017

RePROM Renal Electronic Patient Reported Outcome Measures

UNIVERSITY OF BIRMINGHAM

Short project title: **RePROM**

Full project title: The use of an electronic Patient-Reported Outcome Measure in the Management of Patients with Advanced Chronic Kidney Disease – The RePROM Pilot Trial.

Clinician/Staff Interview Topic Guide

Guidance notes to the interviewer

Note: *If the participant becomes distressed or unwell, the interviewer will adopt the following approaches, dependent upon the participant's wishes:*

1) If the participant wishes, the interviewer will suspend or terminate the interview, and will stay with the participant until they are feeling better.

2) If the participant has another person to provide care, at the request of the participant, the interviewer will either suspend the interview and leave the room, or will terminate the interview completely.

3) If the interviewer feels it is warranted, and if the participant agrees, he will put the participant in contact with an appropriate renal clinician.

4) If the interviewer feels that there is reason to be concerned for the physical/mental health of a participant, he will inform the participant of his intention to take the appropriate action, e.g. call the GP/Consultant.

Points to discuss with the participant prior to signing the consent form

- Recap on key information in the PIS
    - I will be recording this interview, so I have something to help me remember accurately what we talk about today, the only people who will hear the recording are myself and the person producing the transcript (who will sign a confidentiality aggrement), is this ok?
    - If there is anything you find you do not wish to talk about please let me know. I will aim to follow your lead in terms of what we discuss, but if we do stray on to a topic that you are not keen to talk about, tell me straight away and we can discuss something else.

For peer review only - http://bmjopen.bmj.com/site/about/guidelines.xhtml

- We can stop the interview whenever you like. If you would like to take a break, or feel upset or unwell, please let me know and we will suspend or stop the interview entirely.

Written consent will be taken if participant still wishes to take part.

Introduction to Interview

Thank you for agreeing to take part in this interview. The aim of this interview is to discuss your experience of being involved in the RePROM study. There are no ‘right’ or ‘wrong’ answers, we are interested in *your* views based on your experience. I am now going to start the recording.

Begin Interview

Main body of Interview

1) Could you tell us what you felt was good about the recruitment process and whether any aspect could be improved?

Prompts

- Screening, eligibility check
- Approach, consent
- myHealth signup, ePROM training
- Baseline assessment

3) Could you tell us what you felt was good about the follow-up process and whether any aspect could be improved?

6) Could you tell us about your experience using the ePROM system?

Prompts

- Ease of use, usefulness of the data?
- Format of data presentation?
- Alert generation and management.
- What was good about the system and what could be improved?

7) Is there anything about the RePROM project design or implementation that we need to address/improve prior to conducting the planned RCT?

Post Interview – Debrief

- I have no more questions, but I’d like to give you the opportunity to say anything else about the RePROM study, your experience of using the ePROM system, or anything else we’ve discussed today?

For peer review only - http://bmjopen.bmj.com/site/about/guidelines.xhtml

For peer review only

- *Outline what will happen next:* (1) the recording will be typed up and annonymised, then analysed alongside all the other interviews, (2) we will send you a summary of this interview (unless you would prefer that we didn't) and will invite your comments. You do not have to comment on these results if you do not wish to.

- Finally, if you decide that you do not want what you have said today to be included in my research, you will need to tell me this within 5 working days – so by [*insert an actual day, according to timing of interview*]. After this it will be too late to withdraw as I will not be able to untangle what you have told me from what other people have told me.

- Thank you for taking part in the interview today.

For peer review only

For peer review only - http://bmjopen.bmj.com/site/about/guidelines.xhtml

Table S1. Case Report Form (CRF) returns.

Timepoint	CRF	Expected	Received (%)
Baseline	Consent	52	52 (100)
Baseline	CRF	52	52 (100)
Baseline	EQ5D-5L	52	52 (100)
3 Month	CRF	47	47 (100)
3 Month	EQ5D-5L	47	45 (96)
6 Month	CRF	41	41 (100)
6 Month	EQ5D-5L	41	41 (100)
9 Month	CRF	29	29 (100)
9 Month	EQ5D-5L	29	29 (100)
12 Month	CRF	18	18 (100)
12 Month	EQ5D-5L	18	18 (100)

EuroQol five-level five-dimension PROM, EQ5D-5L.

For peer review only

For peer review only - http://bmjopen.bmj.com/site/about/guidelines.xhtml

Table S2. ePROM intervention: overall symptom reporting, notifications and time taken to resolve.

Total number of participants randomised to ePROM intervention	Total number of symptoms reported	Total number of symptom notifications (%)	Total number of participants triggering notifications for severe and current symptoms (%)	Median time taken to resolve in minutes (IQR)
24	579	16 (3)	5 (25)	10 (6.5-22.5)

Electronic Patient-Reported Outcome, ePROM.

For peer review only

Table S3. ePROM intervention: notification pattern by symptom.

	Number of notifications triggered for severe + current symptoms (%)
Itchy/Dry skin	6 (37)
Fatigue	4 (25)
Shortness of breath	2 (13)
Pain	2 (13)
Difficulty sleeping	1 (6)
Ankle swelling	1 (6)
Lack of appetite	0 (0)
Nausea	0 (0)
Problems with fistula	0 (0)
Faintness/dizziness	0 (0)
Restless legs or difficulty keeping legs still	0 (0)
Diarrhoea	0 (0)

Electronic Patient-Reported Outcome, ePROM.

For peer review only - http://bmjopen.bmj.com/site/about/guidelines.xhtml

Table S4. ePROM intervention: staff response to notification.

Staff response to notification	Frequency (%)
Telephone counselling about symptom management	7 (78)
Brought clinic appointment forwards	2 (22)
Imaging/test orders	2 (22)
Medication initiation/change	1 (11)
Other	1 (11)
Referral to A&E	0 (0)
Referral to other NHS service	0 (0)

Electronic Patient-Reported Outcome, ePROM.

For peer review only

For peer review only - http://bmjopen.bmj.com/site/about/guidelines.xhtml

Table S5. Numeric outcome measures by trial arm and data collection point.

	Monthly ePROM reports (N = 24)		Usual care (N = 28)		
	No. (expected)	Mean (95% CI)	No. (expected)	Mean (95% CI)	Adjusted Mean Difference (95% CI)
Systolic BP (mmHg)					
Baseline	24 (24)	147.58 (139.12-156.05)	28 (28)	146.04 (139.94-152.13)	0.72 (-9.51 to 10.95)
3 months	21 (21)	145.14 (138.81-151.48)	26 (26)	140.46 (134.33-146.59)	0.13 (-7.50 to 7.76)
6 months	18 (18)	147.50 (141.92-153.08)	23 (23)	140.17 (132.33-148.02)	2.76 (-6.27 to 11.79)
9 months	11 (12)	141.91 (134.63-149.19)	16 (17)	142.19 (135.14-149.23)	-5.46 (-13.10 to 2.17)
12 months	7 (7)	148.71 (142.25-155.18)	10 (11)	137.70 (126.65-148.75)	7.87 (-5.47 to 21.20)
Diastolic BP (mmHg)					
Baseline	24 (24)	78.83 (75.22-82.45)	28 (28)	77.36 (72.94-81.77)	3.32 (-2.09 to 8.72)
3 months	21 (21)	78.81 (74.72-82.90)	26 (26)	72.85 (68.69-77.01)	4.38 (-0.40 to 9.16)
6 months	18 (18)	76.94 (70.94-82.95)	23 (23)	74.04 (69.66-78.43)	1.32 (-4.87 to 7.52)
9 months	11 (12)	78.00 (70.36-85.64)	16 (17)	78.44 (71.98-84.90)	-0.77 (-9.03 to 7.50)
12 months	7 (7)	79.00 (69.04-88.96)	10 (11)	76.90 (70.44-83.36)	0.24 (-8.92 to 9.40)
Health-Related Quality of Life (EQ-5D-5L index)					
Baseline	24 (24)	0.70 (0.60-0.80)	28 (28)	0.78 (0.71-0.85)	-0.06 (-0.17 to 0.06)
3 months	20 (21)	0.67 (0.53-0.80)	24 (26)	0.76 (0.69-0.84)	-0.03 (-0.13 to 0.07)
6 months	18 (18)	0.66 (0.52-0.80)	23 (23)	0.74 (0.65-0.82)	-0.00 (-0.11 to 0.10)
9 months	12 (12)	0.55 (0.33-0.78)	17 (17)	0.74 (0.66-0.82)	-0.07 (-0.24 to 0.09)

For peer review only - http://bmjopen.bmj.com/site/about/guidelines.xhtml

12 months	7 (7)	0.59 (0.34-0.85)	11 (11)	0.71 (0.61-0.82)	-0.04 (-0.17 to 0.09)
2-year Tangri[1] risk of progression to kidney failure (%)					
Baseline	24 (24)	0.48 (0.40-0.57)	28 (28)	0.43 (0.34-0.51)	0.06 (-0.01 to 0.14)
3 months	21 (21)	0.46 (0.38-0.54)	26 (26)	0.47 (0.38-0.55)	-0.01 (-0.10 to 0.08)
6 months	16 (18)	0.45 (0.34-0.57)	22 (23)	0.43 (0.35-0.52)	-0.01 (-0.12 to 0.10)
9 months	11 (12)	0.46 (0.34-0.58)	16 (17)	0.50 (0.41-0.58)	-0.04 (-0.16 to 0.08)
12 months	5 (7)	0.46 (0.29-0.63)	10 (11)	0.52 (0.38-0.66)	0.01 (-0.21 to 0.22)
eGFR (mL/min/1,73 m2)					
Baseline	24 (24)	14.03 (12.52-15.55)	28 (28)	15.70 (13.93-17.47)	-1.86 (-4.18 to 0.46)
3 months	21 (21)	13.51 (11.89-15.12)	26 (26)	14.07 (12.22-15.91)	0.94 (-0.73 to 2.61)
6 months	18 (18)	13.11 (10.93-15.29)	23 (23)	14.19 (12.49-15.89)	0.28 (-1.86 to 2.43)
9 months	11 (12)	14.54 (12.38-16.70)	16 (17)	13.13 (11.35-14.92)	2.46 (0.30 to 4.63)
12 months	7 (7)	14.13 (12.14-16.12)	10 (11)	12.71 (10.78-14.64)	1.72 (-0.96 to 4.40)
Creatinine (µmol/L)					
Baseline	24 (24)	384.00 (345.84-422.16)	28 (28)	357.54 (316.29-398.78)	39.42 (-9.71 to 88.54)
3 months	21 (21)	380.81 (346.19-415.43)	26 (26)	396.08 (342.23-449.92)	-34.81 (-66.83 to -2.79)
6 months	18 (18)	408.39 (359.35-457.43)	23 (23)	375.96 (334.91-417.00)	-17.82 (-57.55 to 21.92)
9 months	11 (12)	364.45 (305.24-423.67)	16 (17)	399.50 (347.47-451.53)	-41.90 (-88.94 to 5.13)
12 months	7 (7)	370.00 (306.19-433.81)	10 (11)	409.10 (337.29-480.91)	-47.60 (-131.55 to 36.36)
Calcium (µmol/L)					
Baseline	24 (24)	2.24 (2.19-2.29)	28 (28)	2.27 (2.25-2.30)	-0.03 (-0.09 to 0.02)
3 months	21 (21)	2.28 (2.22-2.35)	26 (26)	2.29 (2.24-2.34)	0.02 (-0.04 to 0.08)

For peer review only

For peer review only - http://bmjopen.bmj.com/site/about/guidelines.xhtml

6 months	18 (18)	2.30 (2.25-2.35)	23 (23)	2.34 (2.29-2.39)	-0.01 (-0.07 to 0.04)
9 months	11 (12)	2.37 (2.27-2.47)	16 (17)	2.40 (2.35-2.46)	-0.03 (-0.11 to 0.04)
12 months	6 (7)	2.40 (2.35-2.45)	10 (11)	2.40 (2.29-2.50)	0.01 (-0.08 to 0.10)
Bicarbonate (µmol/L)					
Baseline	24 (24)	20.83 (19.76-21.89)	28 (28)	21.25 (20.33-22.17)	-0.30 (-1.70 to 1.09)
3 months	21 (21)	21.36 (20.13-22.59)	25 (26)	21.30 (20.16-22.45)	0.19 (-1.26 to 1.64)
6 months	17 (18)	20.56 (19.14-21.99)	21 (23)	21.19 (19.97-22.41)	0.49 (-0.92 to 1.91)
9 months	11 (12)	21.82 (19.59-24.04)	15 (17)	20.73 (19.14-22.33)	1.13 (-1.32 to 3.59)
12 months	5 (7)	21.60 (18.93-24.27)	9 (11)	20.67 (17.76-23.57)	1.03 (-2.44 to 4.50)
Phosphate (µmol/L)					
Baseline	24 (24)	1.41 (1.31-1.52)	28 (28)	1.40 (1.30-1.51)	0.01 (-0.14 to 0.16)
3 months	21 (21)	1.47 (1.39-1.55)	25 (26)	1.60 (1.41-1.79)	-0.14 (-0.34 to 0.05)
6 months	17 (18)	1.52 (1.36-1.69)	21 (23)	1.38 (1.23-1.52)	0.06 (-0.12 to 0.25)
9 months	11 (12)	1.45 (1.27-1.62)	14 (17)	1.46 (1.30-1.61)	-0.03 (-0.26 to 0.21)
12 months	5 (7)	1.61 (1.28-1.93)	9 (11)	1.42 (1.25-1.60)	0.31 (0.02 to 0.59)
Albumin (g/L)					
Baseline	24 (24)	40.38 (38.20-42.55)	28 (28)	40.82 (38.98-42.66)	-0.52 (-3.34 to 2.30)
3 months	21 (21)	39.43 (37.50-41.36)	26 (26)	39.58 (37.80-41.36)	0.91 (-0.61 to 2.43)
6 months	18 (18)	37.39 (35.15-39.62)	23 (23)	37.65 (35.90-39.41)	0.24 (-1.56 to 2.04)
9 months	11 (12)	35.27 (33.12-37.42)	16 (17)	36.50 (34.52-38.48)	0.37 (-2.31 to 3.05)
12 months	7 (7)	36.86 (34.42-39.29)	10 (11)	35.10 (32.90-37.30)	1.63 (-1.38 to 4.64)
ACR (mg/mmol)					
Baseline	24 (24)	206.06 (126.92-285.20)	28 (28)	178.08 (109.73-246.43)	23.64 (-66.09 to 113.37)

For peer review only - http://bmjopen.bmj.com/site/about/guidelines.xhtml

3 months	21 (21)	167.31 (101.53-233.09)	26 (26)	149.25 (108.39-190.11)	-19.60 (-63.75 to 24.56)
6 months	16 (18)	182.24 (95.65-268.83)	22 (23)	135.88 (88.78-182.98)	-3.73 (-72.53 to 65.07)
9 months	11 (12)	227.58 (117.37-337.79)	16 (17)	148.23 (97.56-198.90)	0.20 (-84.56 to 84.96)
12 months	5 (7)	175.74 (97.71-253.77)	10 (11)	161.51 (74.67-248.35)	-14.40 (-138.43 to 109.63)
Blood Glucose (mmol/L)					
Baseline	8 (9)	8.36 (6.82-9.90)	11 (12)	6.97 (5.58-8.36)	1.48 (-0.57 to 3.52)
3 months	7 (9)	9.36 (5.39-13.33)	8 (11)	8.74 (5.80-11.68)	-2.18 (-6.22 to 1.87)
6 months	5 (8)	15.88 (3.47-28.29)	5 (10)	7.22 (5.14-9.30)	-2.58 (-13.52 to 8.36)
9 months	4 (6)	8.93 (5.36-12.49)	3 (8)	6.30 (3.84-8.76)	2.12 (-1.40 to 5.64)
12 months	1 (4)	10.70#	2 (5)	5.10 (1.57-8.63)	-
HbA1c (mmol/mol)					
Baseline	5 (9)	57.20 (42.83-71.57)	9 (12)	53.22 (43.98-62.46)	3.18 (-12.52 to 18.87)
3 months	7 (9)	53.29 (43.78-62.79)	7 (11)	46.14 (38.80-53.48)	2.36 (-4.61 to 9.33)
6 months	7 (8)	51.14 (44.40-57.88)	8 (10)	50.63 (40.45-60.80)	-6.00 (-14.06 to 2.05)
9 months	2 (6)	59.50 (52.64-66.36)	3 (8)	52.67 (43.04-62.29)	-
12 months	2 (4)	57.00 (51.12-62.88)	3 (5)	49.33 (36.87-61.80)	-6.58 (-9.21 to -3.96)

#Insufficient data to calculate 95% CI. [1] Tangri N, Stevens LA, Griffith J, et al. A predictive model for progression of chronic kidney disease to kidney failure. Jama. 2011;305(15):1553-1559.[21] Electronic Patient-Reported Outcome Measure, ePROM; blood pressure, BP; EuroQol five-level five-dimension PRO, EQ5D-5L; Estimated Glomerular Filtration Rate, eGFR; Albumin Creatinine Ratio, ACR; glycated haemoglobin, HbA1c.

For peer review only - http://bmjopen.bmj.com/site/about/guidelines.xhtml

Table S6. Binary outcome measures by trial arm and data collection point.

	Monthly ePROM reports (N = 24)		Usual care (N = 28)		
	N[a]	Events (%, 95% CI)	N[a]	Events (%, 95% CI)	Risk Ratio (95% CI)[b]
**Death**					
Baseline to 3 months	24	0 (0, 0-14)	28	0 (0, 0-12)	-
3 to 6 months	21	1 (5, 0-24)	26	0 (0, 0-13)	-
6 to 9 months	18	0 (0, 0-19)	23	0 (0, 0-15)	-
9 to 12 months	12	0 (0, 0-26)	17	0 (0, 0-20)	-
Total		1 (4, 0-21)		0 (0, 0-12)	-
**Kidney failure**					
Baseline to 3 months	24	1 (4, 0-21)	28	4 (14, 4-33)	0.29 (0.30 to 2.44)
3 to 6 months	21	3 (14, 3-36)	26	2 (8, 1-25)	1.86 (0.34 to 10.11)
6 to 9 months	18	3 (17, 4-41)	23	0 (0, 0-15)	-
9 to 12 months	12	1 (8, 0-38)	17	3 (18, 4-43)	0.47 (0.06, 4.01)
Total		8 (33, 16-55)		9 (32, 16-52)	1.04 (0.47 to 2.26)
**Hospitalisation**					
Baseline to 3 months	24	1 (4, 0-12)	28	1 (4, 0-18)	1.17 (0.08 to 17.67)
3 to 6 months	21	2 (10, 1-30)	26	3 (6, 2-30)	0.83 (0.15 to 4.49)
6 to 9 months	19	2 (11, 1-33)	23	2 (9, 1-28)	1.21 (0.19 to 7.80)
9 to 12 months	12	0 (0, 0-26)	17	0 (0, 0-20)	-
Total		5 (21, 7-42)		5[c] (18, 6-37)	1.17 (0.38 to 3.55)

Electronic patient-reported outcome, ePROM. [a]Number of participants in the study at start of timepoint.
[b]unadjusted risk ratios are reported due to the low frequencies of events. [c]This figure denotes the number of unique individuals with at least one hospital stay during the study. Individuals can have more than one hospital stay.

For peer review only - http://bmjopen.bmj.com/site/about/guidelines.xhtml

For peer review only

For peer review only - http://bmjopen.bmj.com/site/about/guidelines.xhtml

Table S7. Protocol deviations.

	Allocation	
Protocol deviation	Monthly ePROM reports (N = 24)	Usual care (N = 28)
Software error 19-Jun-2019 [resolved]	1	0
Informed Consent Form error	0	1

Electronic Patient-Reported Outcome, ePROM.

For peer review only

For peer review only - http://bmjopen.bmj.com/site/about/guidelines.xhtml

Table S8. Free text comments.

If you have had any other symptoms or problems that you would like the kidney team to be aware of please outline below:
A stomach upset overnight one evening. with indigestion. Resolved by taking a couple of Bisodol tablets
Anal fistulas
Ankle and lower leg swelling since [Date Redacted]. New symptom. Goes away overnight. No new shortness of breath.
Arthritis
Arthritis. psoriasis. diabetes. high blood pressure
Arthritis/psoriasis
been very pale and colleagues have commented on a "yellow" tinge
Blocked sinus's
Breathlessness increasing. Clinic [Date Redacted] - fluid at base of right lung
constipation
constipation. which is improving
Cough productive of clear mucus
Difficulty concentrating
Difficulty concentrating and feeling cold
Difficulty concentrating. Night swears.
Dry mouth. husky voice.
During last night's sleep. I woke up in the middle of the night [Date Redacted] and found that my pyjama top was soaked in sweat. Otherwise. felt OK?
During my last visit to the Renal team. Quinine Sulfate tablets were proscribed to assist with random over night leg cramps. Just to confirm that this medication has dramatically reduced the incidence of cramps. thank you.
Excessive mucus. no cold symptoms. but caused me to vomit and retch. Slight nosebleeds. Very poor appetite. UTI. Antibiotics prescribed by renal vascular team [Date Redacted] when doing first stage fistula. Ciprox
Feel a bit light headed this afternoon
Feeling cold
Feeling cold.
Felt very tired on [Dates Redacted] plus a stomach upset. probably as a result of the proceedure carried out [Date Redacted]?

For peer review only - http://bmjopen.bmj.com/site/about/guidelines.xhtml

For the last two nights I have had difficulty in sleeping after the first three hours or so. Additionally last night when I awoke in the middle of the night for a toilet break I had been sweating a very great deal. which is unusual for me.
Headaches. painful feet. like electric shocks
Increasing sleepiness. eg nodding off after meals
Inpatient [Dates redacted]
Joint swelling...pain in joints...headaches
Loss of taste
More sleepy' Prone to nod off
My bladder control is proving difficult. especially if I travel any distance. After two hours traveling. I often need to stop to empty my bladder and don't get much warning. This means I have to always be on the look out for a toilet where ever I go.
no
No
Not that I'm aware of.
No.
None
none
None
None at this time.
none known
None known
None.
Not that I am aware of
Not that I know of.
Pedal oedema - This was the presenting symptom to the team
Productive cough
Rash over upper body in small patches
Really bad cold
Severe and constant gout inflammatory knee joint

For peer review only - http://bmjopen.bmj.com/site/about/guidelines.xhtml

Severe headaches
Since [Date Redacted] I have had swollen ankles and legs. This goes away overnight. This is a new symptom. I have not been SOB.
Sleepiness previously reported has improved
Some nights I have been getting up three times to pass urine. However. I have just been given compression stockings by a Lymphoedema clinic to help with my swollen legs caused by taking Felodipine (mostly). This might help the problem...
Swelling in ankles due to hospitalisation. diarrhoea due to IV antibiotics for eye infection
Tending to drift off to sleep during the day more often
The Kidney team is aware and treatment is ongoing
Wheezy cough

For peer review only

For peer review only - http://bmjopen.bmj.com/site/about/guidelines.xhtml

Table S9. Summary of qualitative findings regarding intervention positives/negatives and suggested system changes based on 1 HCP interview.

Theme	Subtheme	Illustrative quote
Intervention positives	Questionnaire data picked up by care team and acted upon	"I would always start the consultation with thank you for taking part, I've been looking at this, shall we look at it together, I see that here you reported this, would you like to tell me a bit about that… patients… seemed really pleased that we were looking at it and using it and it was meaningful. Because clearly it was something that they were taking time and trouble to do. And so, for them knowing that we were using it and taking it seriously was probably a really good thing." [HCP 01]
	Used free-text comments to communicate with nursing staff	"Initially I was filling the form in and putting very little additional information on. Latterly I was putting a lot more information on and I was very pleased on two occasions that when I went for my renal check-up, the points that I'd made had been noticed and were brought up… it was an additional form of communication in that if I'd got a concern or something was happening, I could put it on the form… and you could use it to answer questions then as to how you were coping, what you were doing and how you were feeling." [Patient 01]; "I think that was the good thing about the free text because it did allow people to tell us things that we hadn't particularly asked about." [HCP 01]
	Useful tool to guide consultation	"It was a nice tool to guide consultation. So normally you've just got your clinic letter from your previous visit, and that gives you a fair idea of the kind of things that you're going to talk to the patient about based on the things that you've talked to them about before and the active medicine which you've identified. But having the RePROM as well often highlighted things that were completely off the radar. And I think it's perfectly likely the patient would have mentioned it themselves anyway, it meant that you knew in advance and you were able to get straight into it, rather than it being the kind of thing that they casually mention as they're leaving the room. So, you have a bit more time to explore things in a bit more detail I think." [HCP 01]
	Would allow remote follow up post-COVD	"…now our capacity to see patients face-to-face has reduced by about 75% because of the need for social distancing. So actually, now that they're almost all phone and video consultations something like RePROM is more important than ever because that does give patients a bit more of an ability to… to contact us and tell us things that they were worried about in between their reviews." [HCP 01]

For peer review only - http://bmjopen.bmj.com/site/about/guidelines.xhtml

Intervention negatives	Need to open up a different system precluded use in Multidisciplinary Team (MDT) meetings	"We had lots of great ideas at the beginning about how we'd look at it and the MDT when we looked forward to the next clinic but actually the MDT's are so busy and there were so many people to get through that it just a quick, look at the blurb, what are the outstanding issues, move on. And so, we didn't use it because that would have meant getting the Portal up rather than just PICS and waiting for it to load and so no, we didn't use it in the MDT." [HCP 01]
Intervention acceptability	Patient acceptance of remote follow up/ability to engage with technology	"I guess COVID has taught us a couple of things. The first thing is that we've all said, a lot of people have said, oh patients won't cope with phone consultations, and they certainly won't cope with video consultations. Patients are not very tech savvy, they won't be able to do it, they're all very elderly, a lot of them don't speak any English and it would be a complete disaster. And that's not completely been our experience, people seem to have adapted to phone consultations and video consultations really quite well." [HCP 01]
	Enhance/simplify interface	"The only thing I can think of as far as improving the system is to make it more user-friendly basically… navigating your way through the electronic system… could be made a bit easier." [Patient 08]; "I think the practical obstacle… was that patients find the interface difficult." [HCP 01]
	Incorporate questions around psychological wellbeing/mood	"I think just having that questionnaire to see how your mood is and how you can look back on it and see where, like, how you can improve and how you can change it slightly and try and move on from there…" [Patient 10]; "I'm not particularly surprised that people mentioned that [anxiety & depression], and I think that's reasonable. I think in a future iteration we probably should try and capture that." [HCP 01]
	Consider expanding use of the system to dialysis populations	"I definitely think that doing something like this in terms of the dialysis population would be massively useful… Compared to the very close supervision that they had in the year, six months before they started dialysis. A year to six months after they've started dialysis that is an entirely different experience… anecdotally a lot of patients say, oh gosh I used to come to clinic and see doctors and nurses and dieticians and now I'm at my satellite unit I see the nurses all the time and I occasionally see a dietician but it doesn't feel the same… I think they find that quite a worrying time, and maybe having something like this to support them particularly in that transition would be really useful." [HCP 01]
	Consider use of a central platform to aid roll out to other centres	"I think the difficulty when we think about rolling it out to other places is that everywhere will have a different electronic patient record type system… we'll have to think about how the IT works in each of those places..." [HCP 01]

For peer review only - http://bmjopen.bmj.com/site/about/guidelines.xhtml

CONSORT 2010 checklist of information to include when reporting a pilot or feasibility trial*

Section/Topic	Item No	Checklist item	Reported on page No
**Title and abstract**			
	1a	Identification as a pilot or feasibility randomised trial in the title	1
	1b	Structured summary of pilot trial design, methods, results, and conclusions (for specific guidance see CONSORT abstract extension for pilot trials)	2
**Introduction**			
Background and objectives	2a	Scientific background and explanation of rationale for future definitive trial, and reasons for randomised pilot trial	4
	2b	Specific objectives or research questions for pilot trial	4
**Methods**			
Trial design	3a	Description of pilot trial design (such as parallel, factorial) including allocation ratio	5-7
	3b	Important changes to methods after pilot trial commencement (such as eligibility criteria), with reasons	5
Participants	4a	Eligibility criteria for participants	5
	4b	Settings and locations where the data were collected	5
	4c	How participants were identified and consented	6
Interventions	5	The interventions for each group with sufficient details to allow replication, including how and when they were actually administered	6
Outcomes	6a	Completely defined prespecified assessments or measurements to address each pilot trial objective specified in 2b, including how and when they were assessed	6-7
	6b	Any changes to pilot trial assessments or measurements after the pilot trial commenced, with reasons	n/a
	6c	If applicable, prespecified criteria used to judge whether, or how, to proceed with future definitive trial	n/a
Sample size	7a	Rationale for numbers in the pilot trial	7
	7b	When applicable, explanation of any interim analyses and stopping guidelines	n/a
Randomisation:			
Sequence generation	8a	Method used to generate the random allocation sequence	6
	8b	Type of randomisation(s); details of any restriction (such as blocking and block size)	6
Allocation concealment mechanism	9	Mechanism used to implement the random allocation sequence (such as sequentially numbered containers), describing any steps taken to conceal the sequence until interventions were assigned	6

For peer review only

Implementation	10	Who generated the random allocation sequence, who enrolled participants, and who assigned participants to interventions	6
Blinding	11a	If done, who was blinded after assignment to interventions (for example, participants, care providers, those assessing outcomes) and how	6
	11b	If relevant, description of the similarity of interventions	n/a
Statistical methods	12	Methods used to address each pilot trial objective whether qualitative or quantitative	7
**Results**			
Participant flow (a diagram is strongly recommended)	13a	For each group, the numbers of participants who were approached and/or assessed for eligibility, randomly assigned, received intended treatment, and were assessed for each objective	8, Fig. 1
	13b	For each group, losses and exclusions after randomisation, together with reasons	8, Fig. 1
Recruitment	14a	Dates defining the periods of recruitment and follow-up	8
	14b	Why the pilot trial ended or was stopped	8
Baseline data	15	A table showing baseline demographic and clinical characteristics for each group	Table 1
Numbers analysed	16	For each objective, number of participants (denominator) included in each analysis. If relevant, these numbers should be by randomised group	Tables 2-4, and supplementary appendix
Outcomes and estimation	17	For each objective, results including expressions of uncertainty (such as 95% confidence interval) for any estimates. If relevant, these results should be by randomised group	8-23, Tables 2-4 and supplementary appendix
Ancillary analyses	18	Results of any other analyses performed that could be used to inform the future definitive trial	8-23
Harms	19	All important harms or unintended effects in each group (for specific guidance see CONSORT for harms)	8
	19a	If relevant, other important unintended consequences	n/a
**Discussion**			
Limitations	20	Pilot trial limitations, addressing sources of potential bias and remaining uncertainty about feasibility	24-26
Generalisability	21	Generalisability (applicability) of pilot trial methods and findings to future definitive trial and other studies	24-26
Interpretation	22	Interpretation consistent with pilot trial objectives and findings, balancing potential benefits and harms, and considering other relevant evidence	24-26
	22a	Implications for progression from pilot to future definitive trial, including any proposed amendments	24-26
**Other information**			
Registration	23	Registration number for pilot trial and name of trial registry	5

For peer review only

Protocol	24	Where the pilot trial protocol can be accessed, if available	5
Funding	25	Sources of funding and other support (such as supply of drugs), role of funders	27
	26	Ethical approval or approval by research review committee, confirmed with reference number	5

Citation: Eldridge SM, Chan CL, Campbell MJ, Bond CM, Hopewell S, Thabane L, et al. CONSORT 2010 statement: extension to randomised pilot and feasibility trials. BMJ. 2016;355.
*We strongly recommend reading this statement in conjunction with the CONSORT 2010, extension to randomised pilot and feasibility trials, Explanation and Elaboration for important clarifications on all the items. If relevant, we also recommend reading CONSORT extensions for cluster randomised trials, non-inferiority and equivalence trials, non-pharmacological treatments, herbal interventions, and pragmatic trials. Additional extensions are forthcoming: for those and for up to date references relevant to this checklist, see www.consort-statement.org.

For peer review only
